# Supplementary material for: Implementing a Screening, Brief Intervention, and Referral to Treatment Curriculum for Medical Students on their Emergency Department Rotation
Source: MedEdPORTAL. 2026 Jan 13;22:11569. doi: 10.15766/mep_2374-8265.11569 (PMC12796009; doi:10.15766/mep_2374-8265.11569)
Supplement: Supplementary file 1 — Medical Student MI-SBIRT Curriculum.pptxAlcohol Use Disorder Identification Test.docxDrug Abuse Screening Test (DAST-10).docxSBIRT Algorithm.docxSP Case Descriptions.docxSP Case.docxStudent OSCE Instructions.docxSubstance Use Facts Sheet.docxSBIRT Brief Intervention Card.docxSample OSCE Schedule.xlsxPatient Follow-Up Guide.docxStudent SBIRT Patient Follow-Up Survey.docxMI-SBIRT Attitudes and Preparedness Survey.docxPre- and Postcurriculum Assessment.docxStudent-Administered SBIRT Form.docxPost-SBIRT Patient Feedback Form.docxOSCE Score Sheet.docxExceeds Criteria.docxStudent Workflow and Protocol.docx [file mep_2374-8265.11569-s001.zip › A. Medical Student MI-SBIRT Curriculum.pptx]

## Slide 1
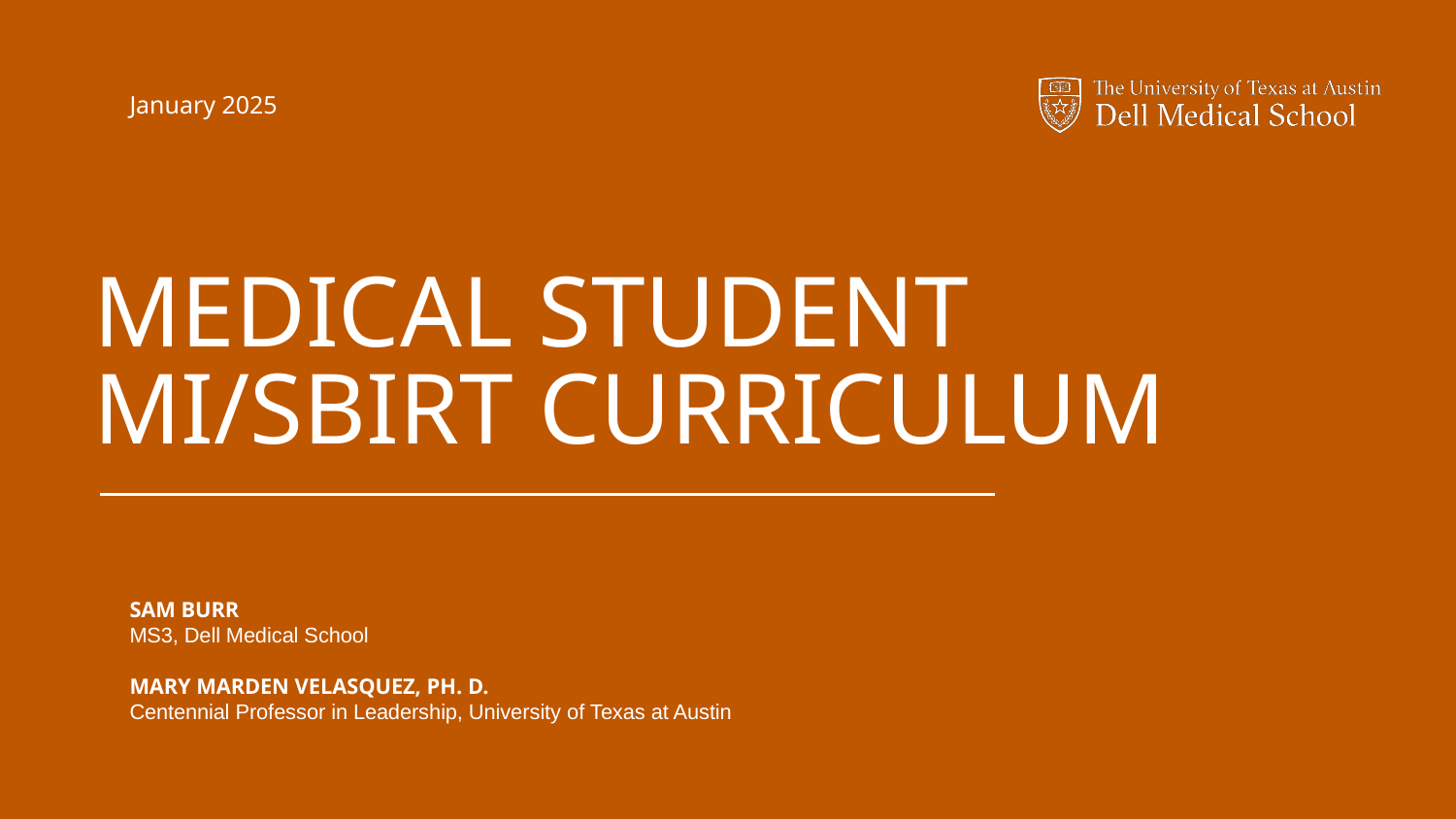

January 2025
# MEDICAL STUDENT MI/SBIRT CURRICULUM
SAM BURR
MS3, Dell Medical School
MARY MARDEN VELASQUEZ, PH. D.
Centennial Professor in Leadership, University of Texas at Austin

## Slide 2
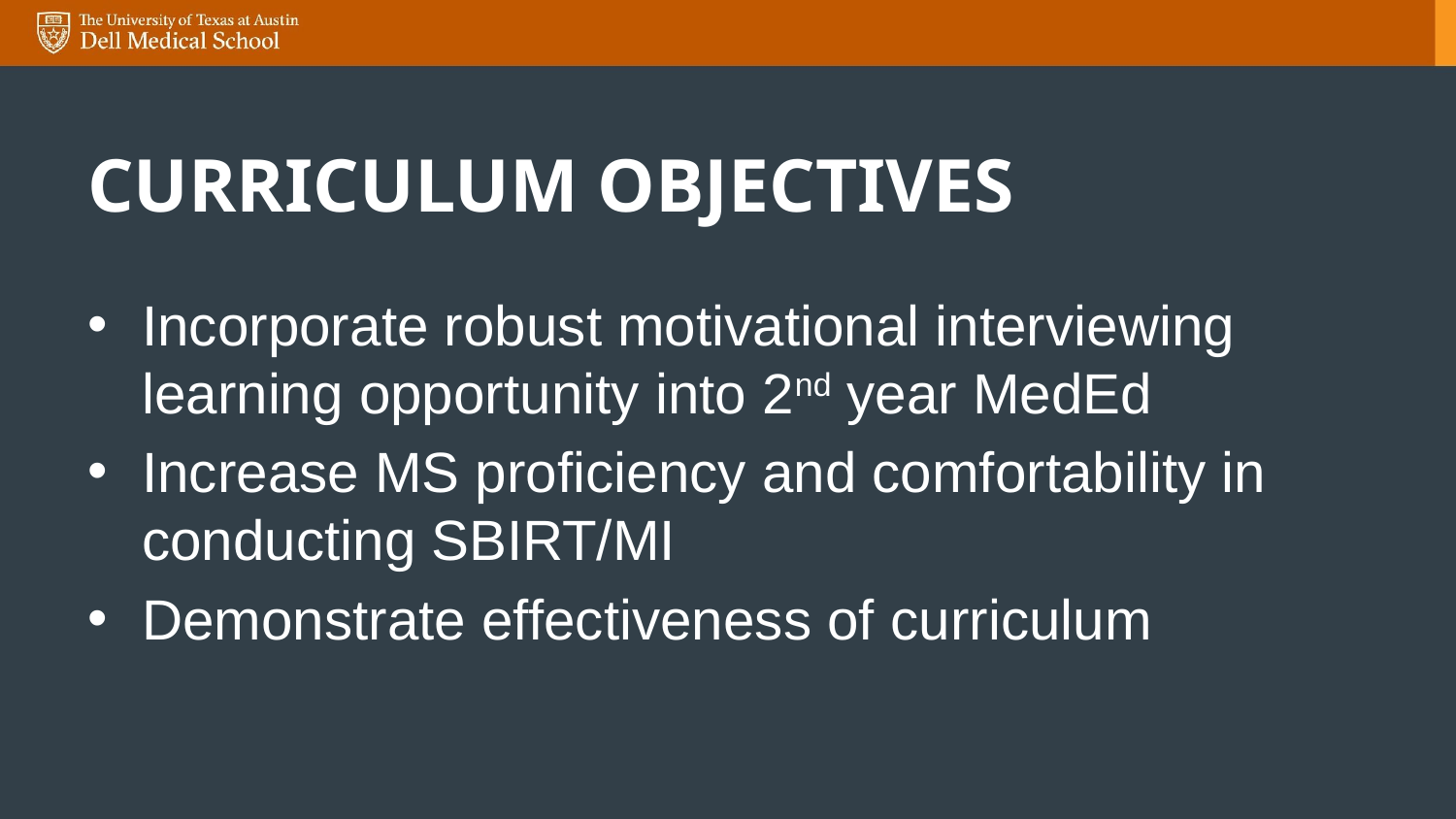

# CURRICULUM OBJECTIVES
Incorporate robust motivational interviewing learning opportunity into 2nd year MedEd
Increase MS proficiency and comfortability in conducting SBIRT/MI
Demonstrate effectiveness of curriculum

## Slide 3
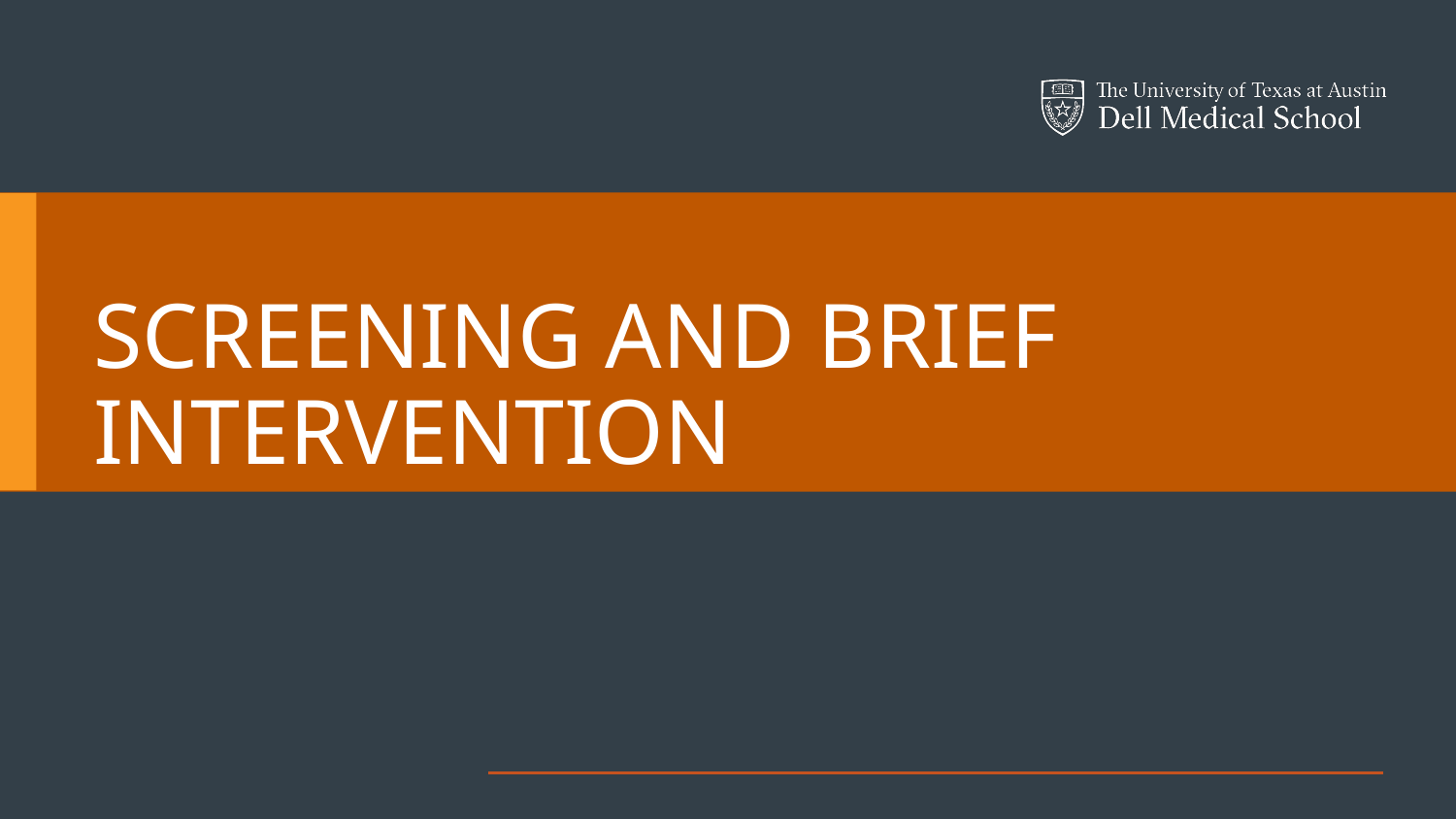

# SCREENING AND BRIEF INTERVENTION

## Slide 4
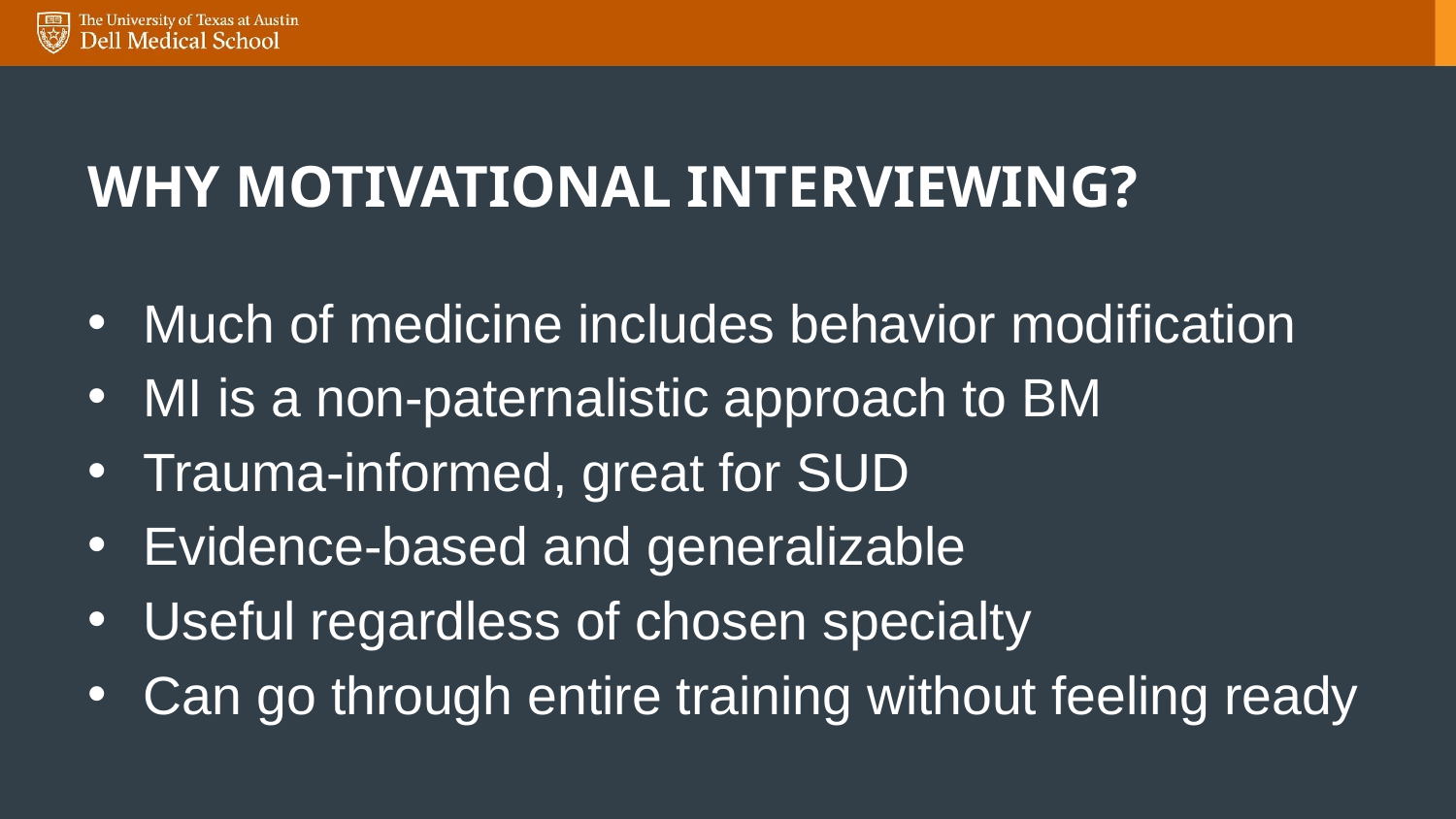

# WHY MOTIVATIONAL INTERVIEWING?
Much of medicine includes behavior modification
MI is a non-paternalistic approach to BM
Trauma-informed, great for SUD
Evidence-based and generalizable
Useful regardless of chosen specialty
Can go through entire training without feeling ready

## Slide 5
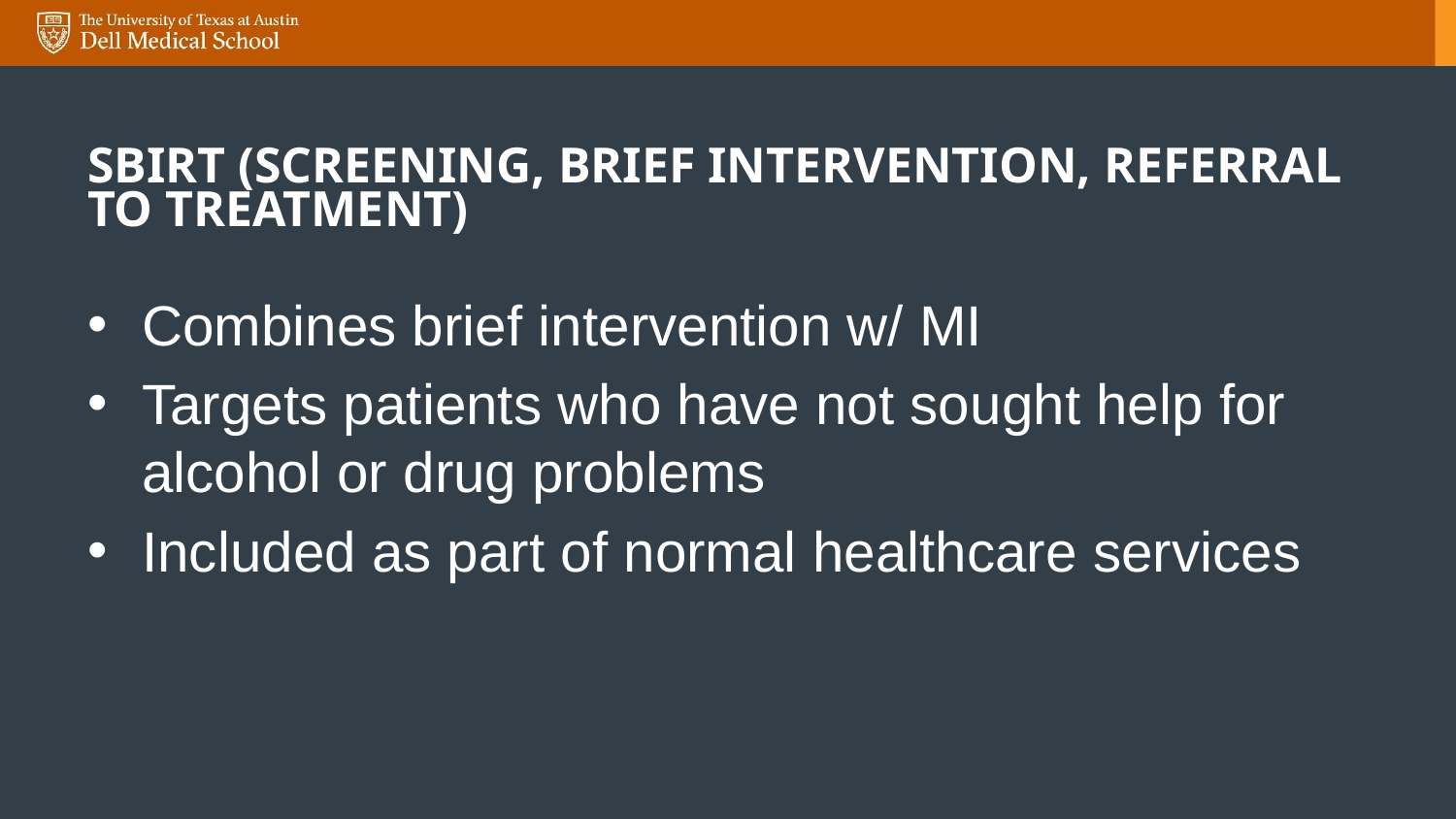

# SBIRT (SCREENING, BRIEF INTERVENTION, REFERRAL TO TREATMENT)
Combines brief intervention w/ MI
Targets patients who have not sought help for alcohol or drug problems
Included as part of normal healthcare services

## Slide 6
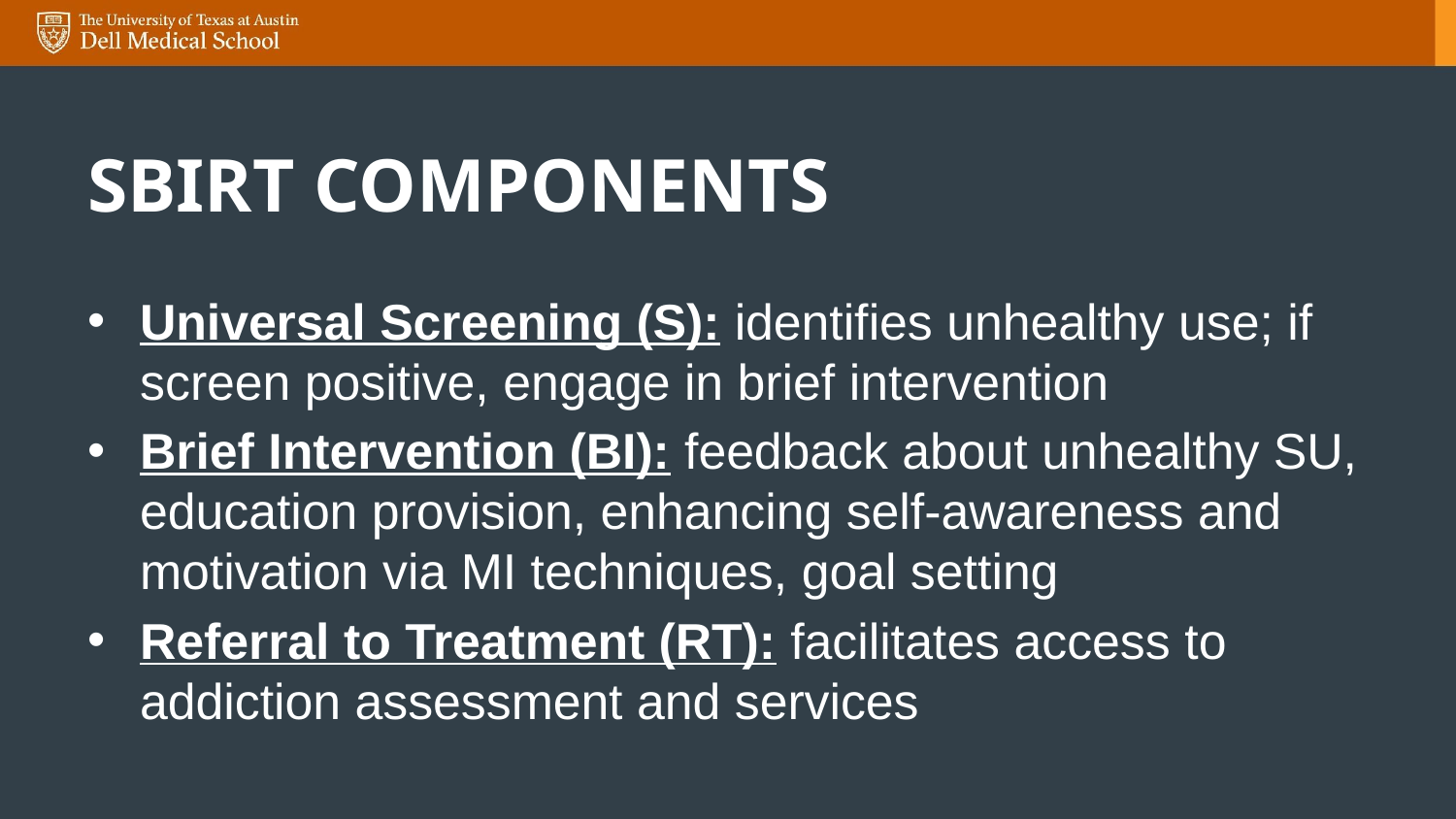

# SBIRT COMPONENTS
Universal Screening (S): identifies unhealthy use; if screen positive, engage in brief intervention
Brief Intervention (BI): feedback about unhealthy SU, education provision, enhancing self-awareness and motivation via MI techniques, goal setting
Referral to Treatment (RT): facilitates access to addiction assessment and services

## Slide 7
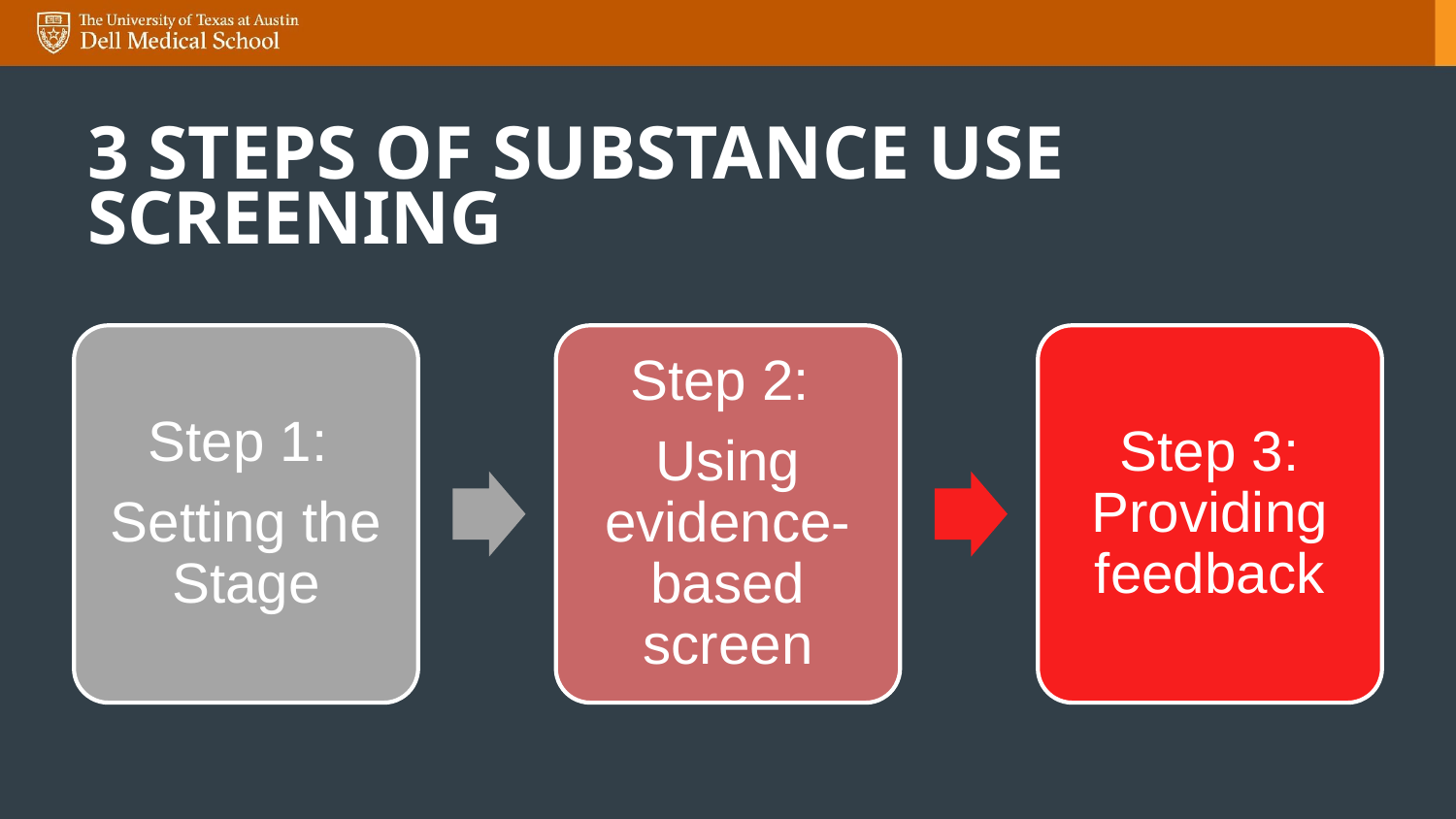

# 3 STEPS OF SUBSTANCE USE SCREENING
Step 1:
Setting the Stage
Step 2:
Using evidence- based screen
Step 3: Providing feedback

## Slide 8
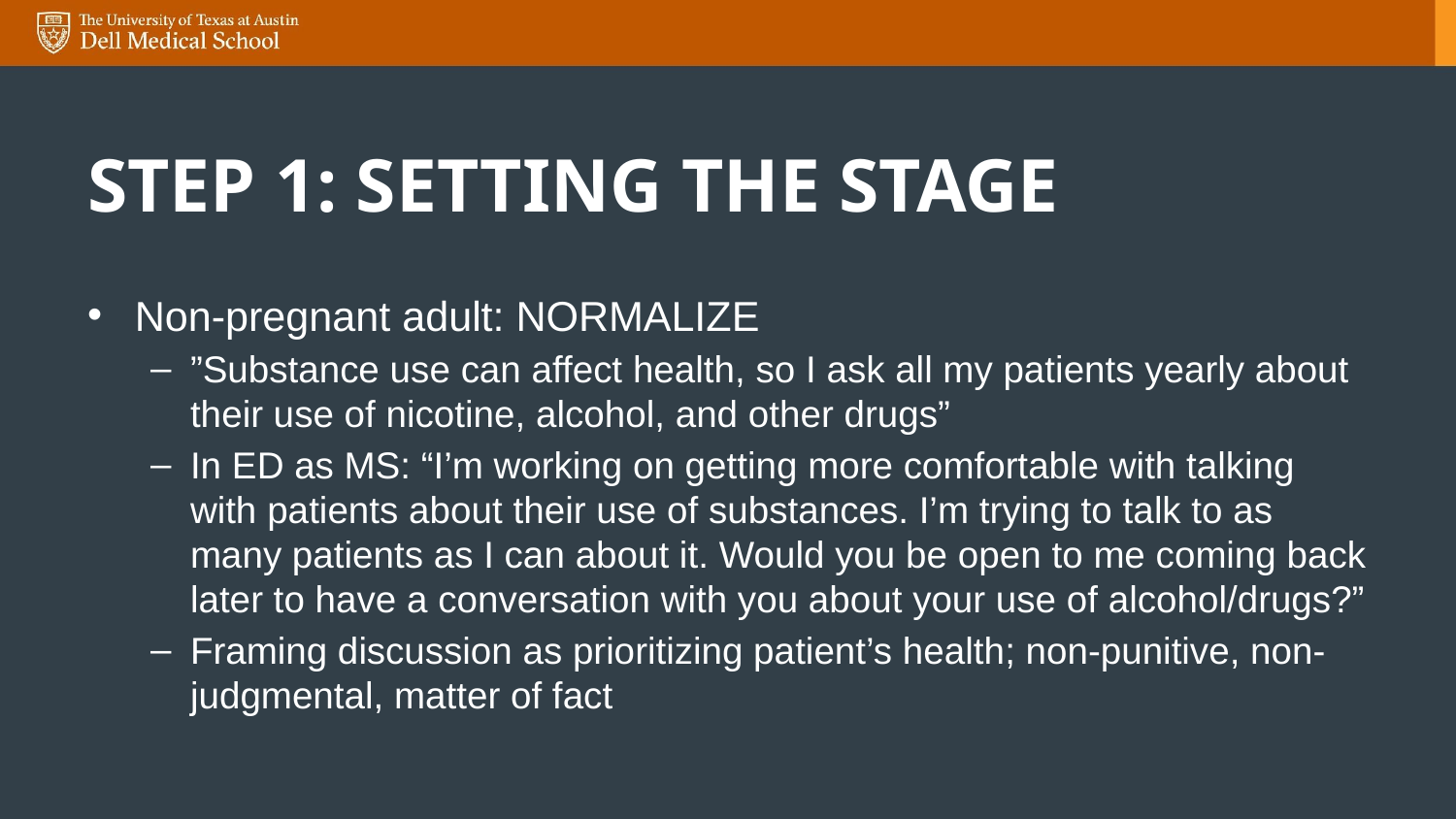

# STEP 1: SETTING THE STAGE
Non-pregnant adult: NORMALIZE
”Substance use can affect health, so I ask all my patients yearly about their use of nicotine, alcohol, and other drugs”
In ED as MS: “I’m working on getting more comfortable with talking with patients about their use of substances. I’m trying to talk to as many patients as I can about it. Would you be open to me coming back later to have a conversation with you about your use of alcohol/drugs?”
Framing discussion as prioritizing patient’s health; non-punitive, non-judgmental, matter of fact

## Slide 9
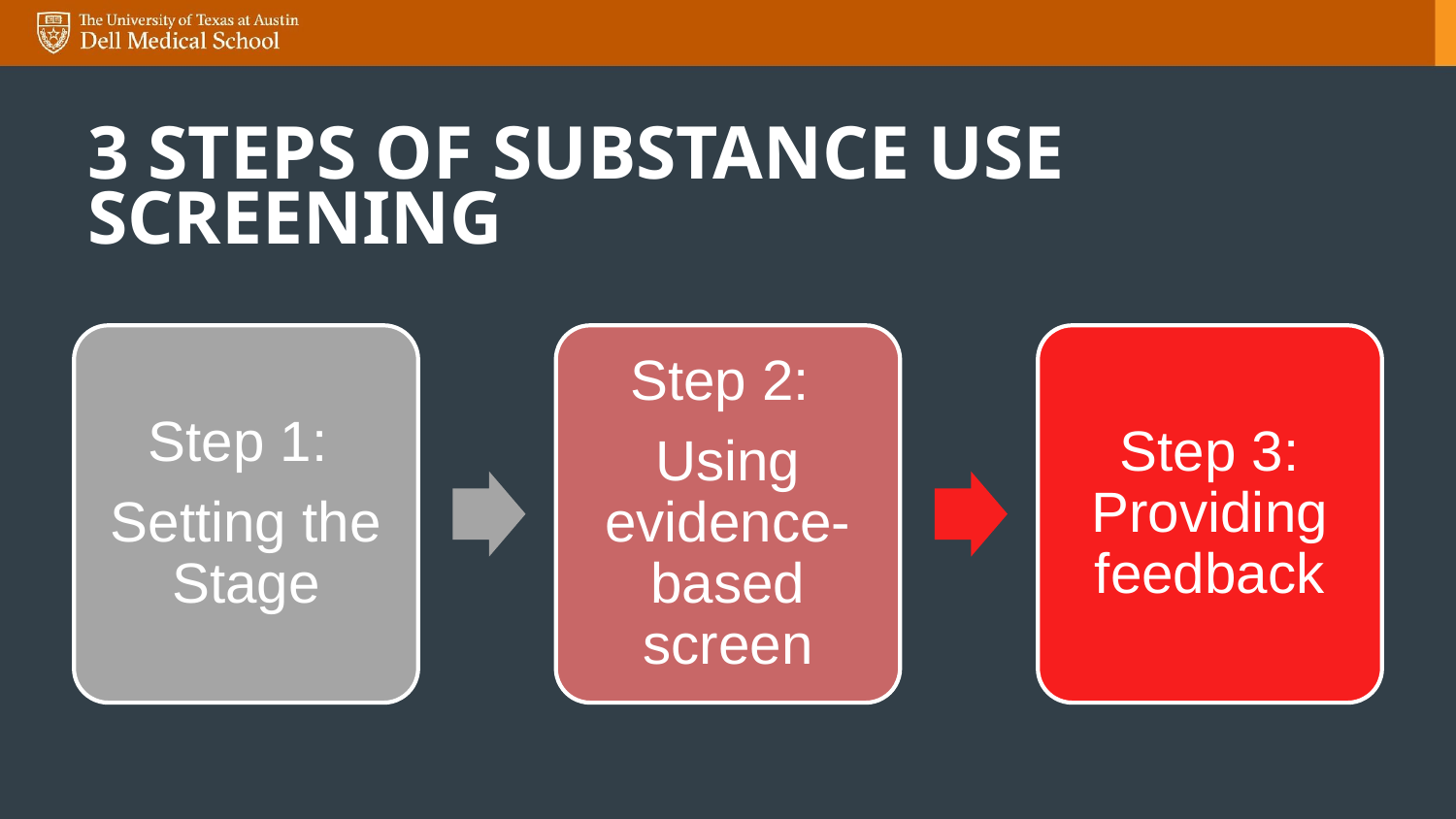

# 3 STEPS OF SUBSTANCE USE SCREENING
Step 1:
Setting the Stage
Step 2:
Using evidence- based screen
Step 3: Providing feedback

## Slide 10
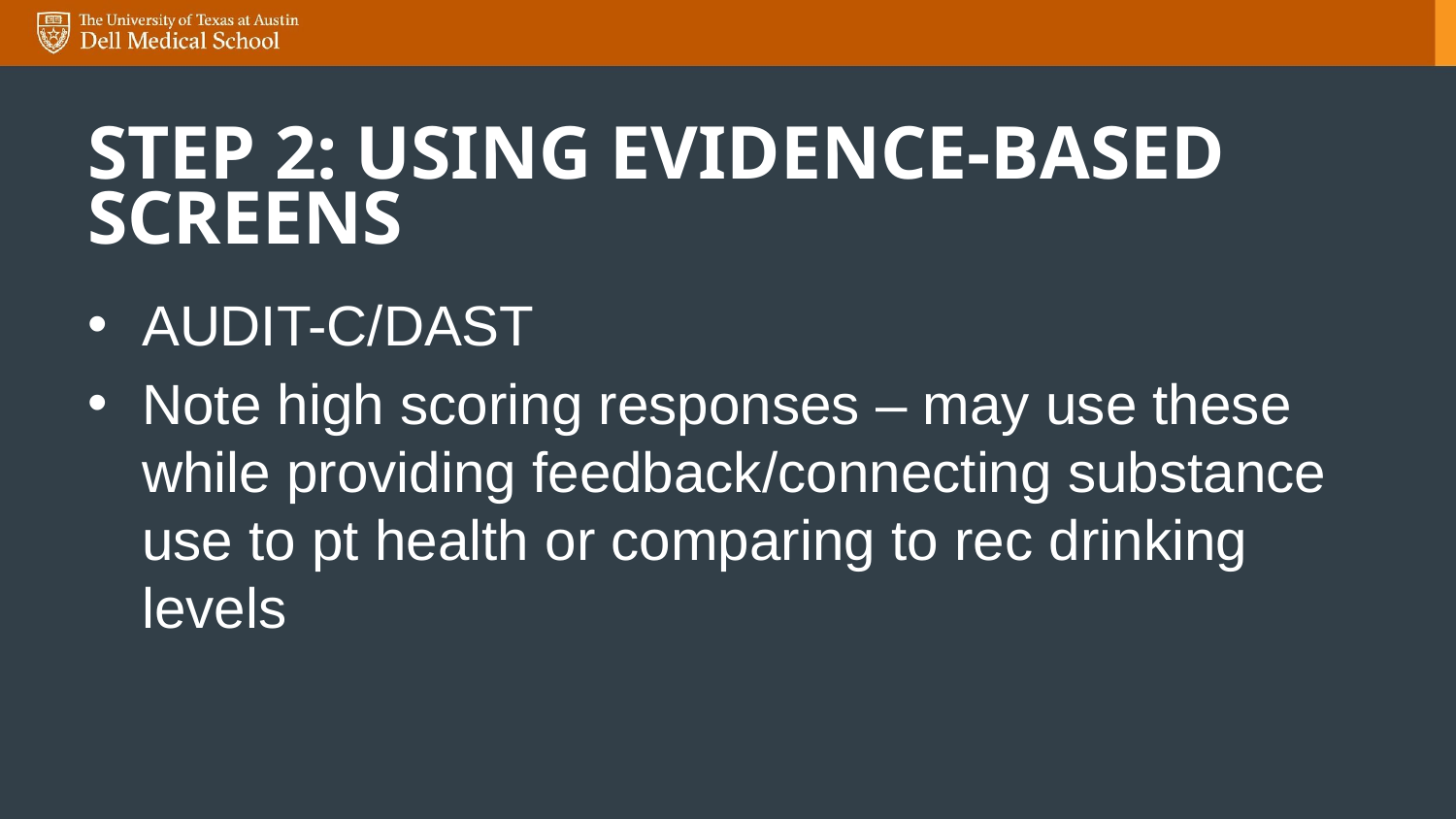

# STEP 2: USING EVIDENCE-BASED SCREENS
AUDIT-C/DAST
Note high scoring responses – may use these while providing feedback/connecting substance use to pt health or comparing to rec drinking levels

## Slide 11
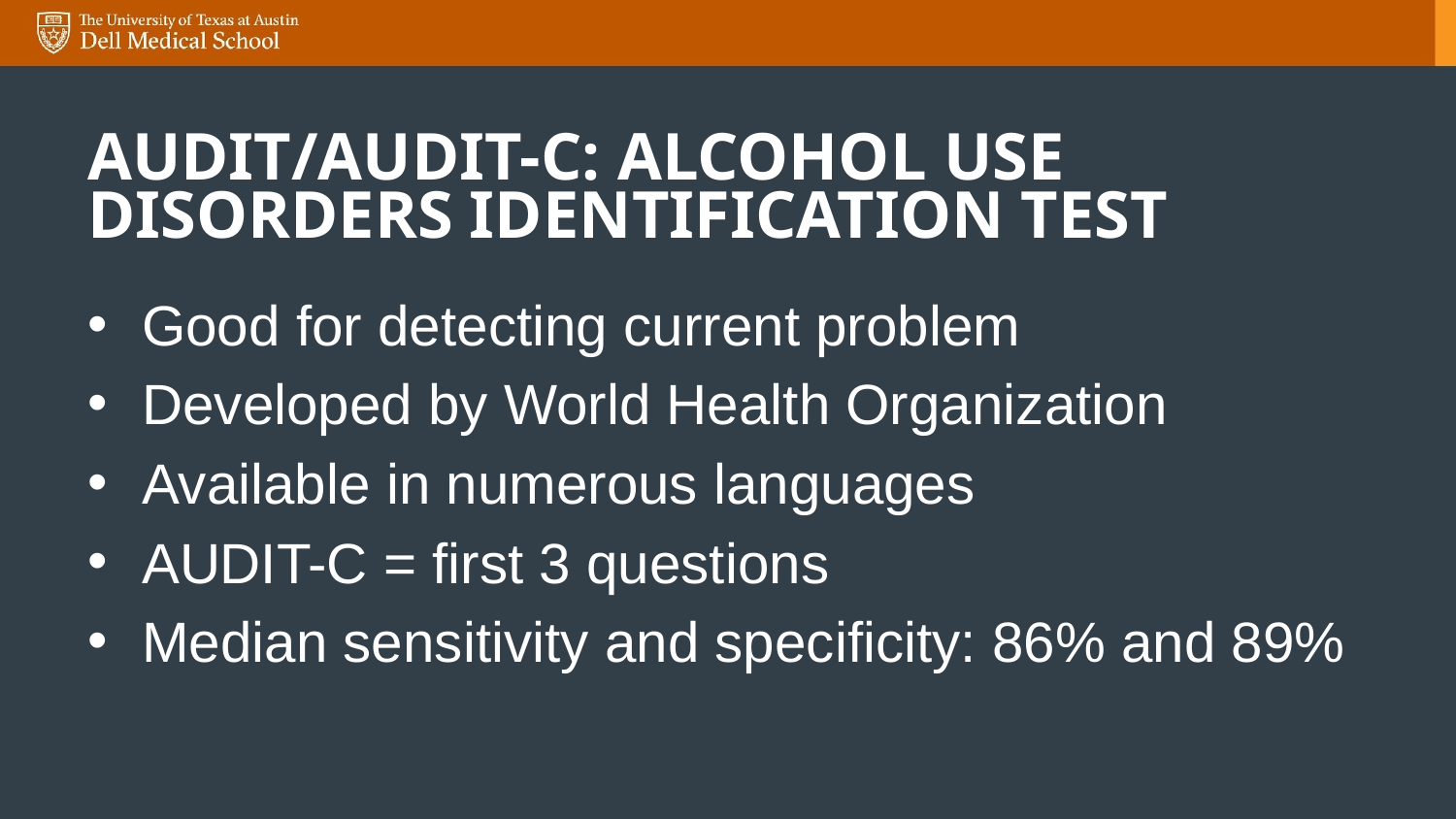

# AUDIT/AUDIT-C: ALCOHOL USE DISORDERS IDENTIFICATION TEST
Good for detecting current problem
Developed by World Health Organization
Available in numerous languages
AUDIT-C = first 3 questions
Median sensitivity and specificity: 86% and 89%

## Slide 12
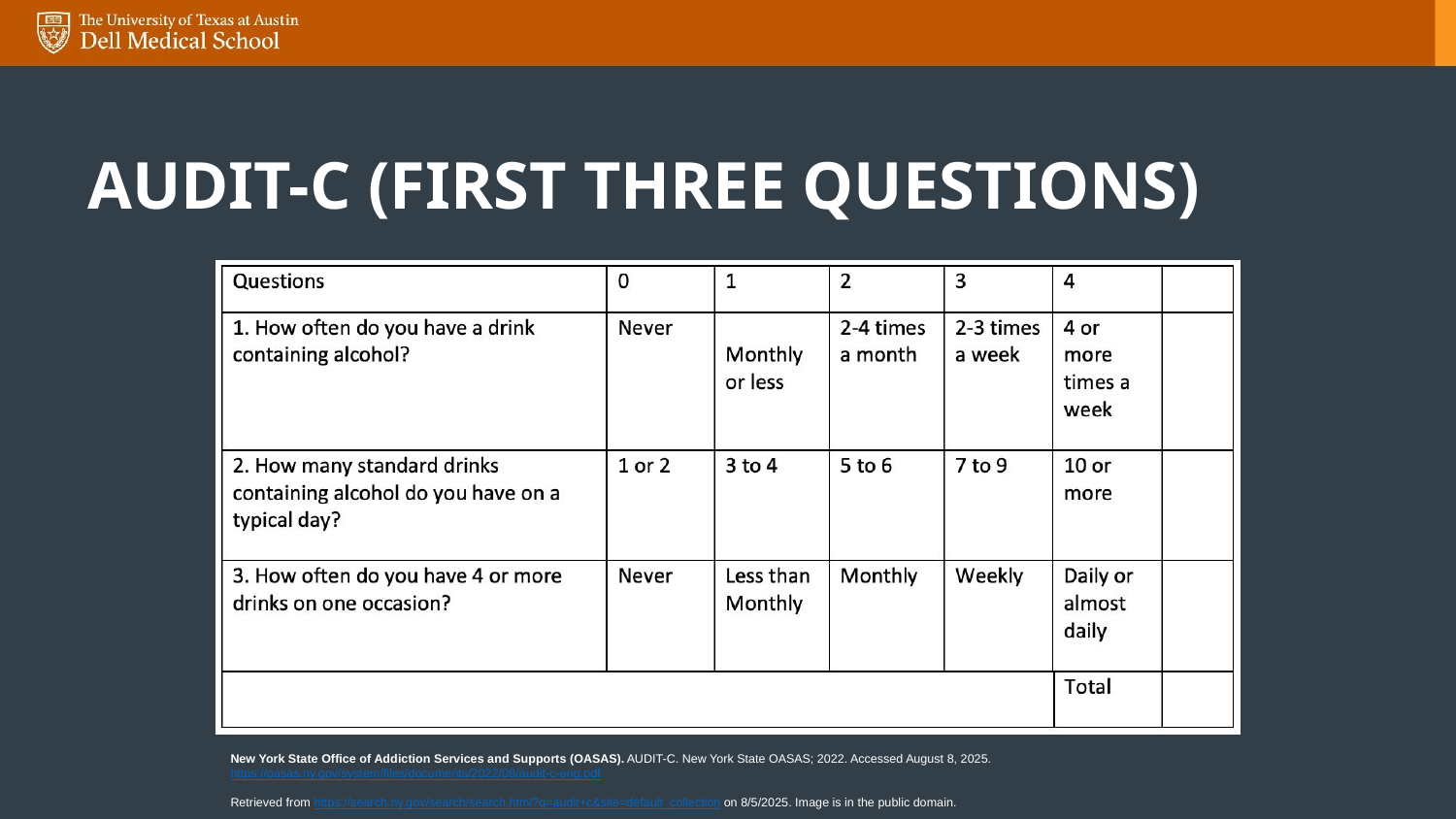

# AUDIT-C (FIRST THREE QUESTIONS)
New York State Office of Addiction Services and Supports (OASAS). AUDIT-C. New York State OASAS; 2022. Accessed August 8, 2025. https://oasas.ny.gov/system/files/documents/2022/08/audit-c-eng.pdf
Retrieved from https://search.ny.gov/search/search.html?q=audit+c&site=default_collection on 8/5/2025. Image is in the public domain.

## Slide 13
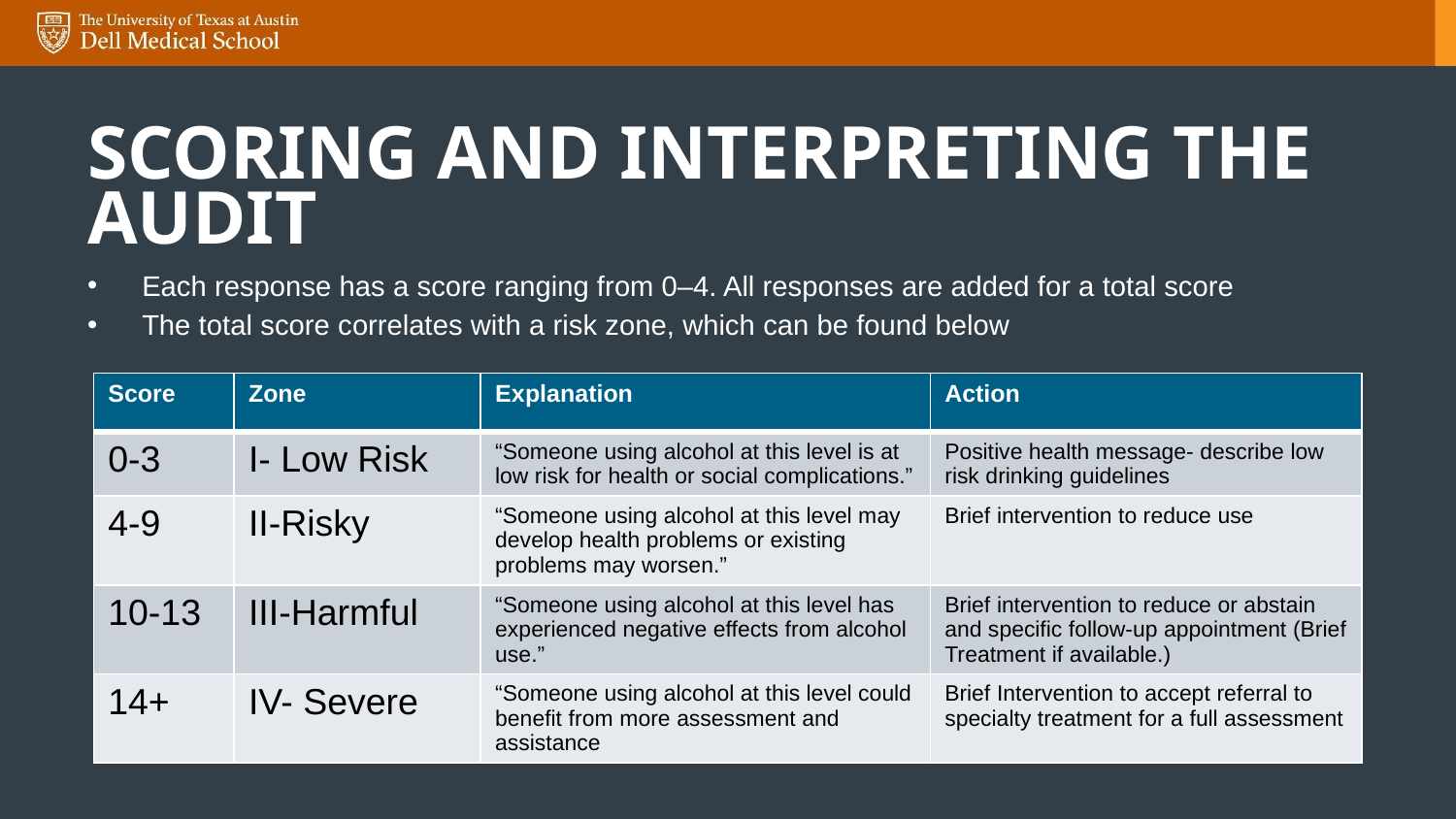

# SCORING AND INTERPRETING THE AUDIT
Each response has a score ranging from 0–4. All responses are added for a total score
The total score correlates with a risk zone, which can be found below
| Score | Zone | Explanation | Action |
| --- | --- | --- | --- |
| 0-3 | I- Low Risk | “Someone using alcohol at this level is at low risk for health or social complications.” | Positive health message- describe low risk drinking guidelines |
| 4-9 | II-Risky | “Someone using alcohol at this level may develop health problems or existing problems may worsen.” | Brief intervention to reduce use |
| 10-13 | III-Harmful | “Someone using alcohol at this level has experienced negative effects from alcohol use.” | Brief intervention to reduce or abstain and specific follow-up appointment (Brief Treatment if available.) |
| 14+ | IV- Severe | “Someone using alcohol at this level could benefit from more assessment and assistance | Brief Intervention to accept referral to specialty treatment for a full assessment |

## Slide 14
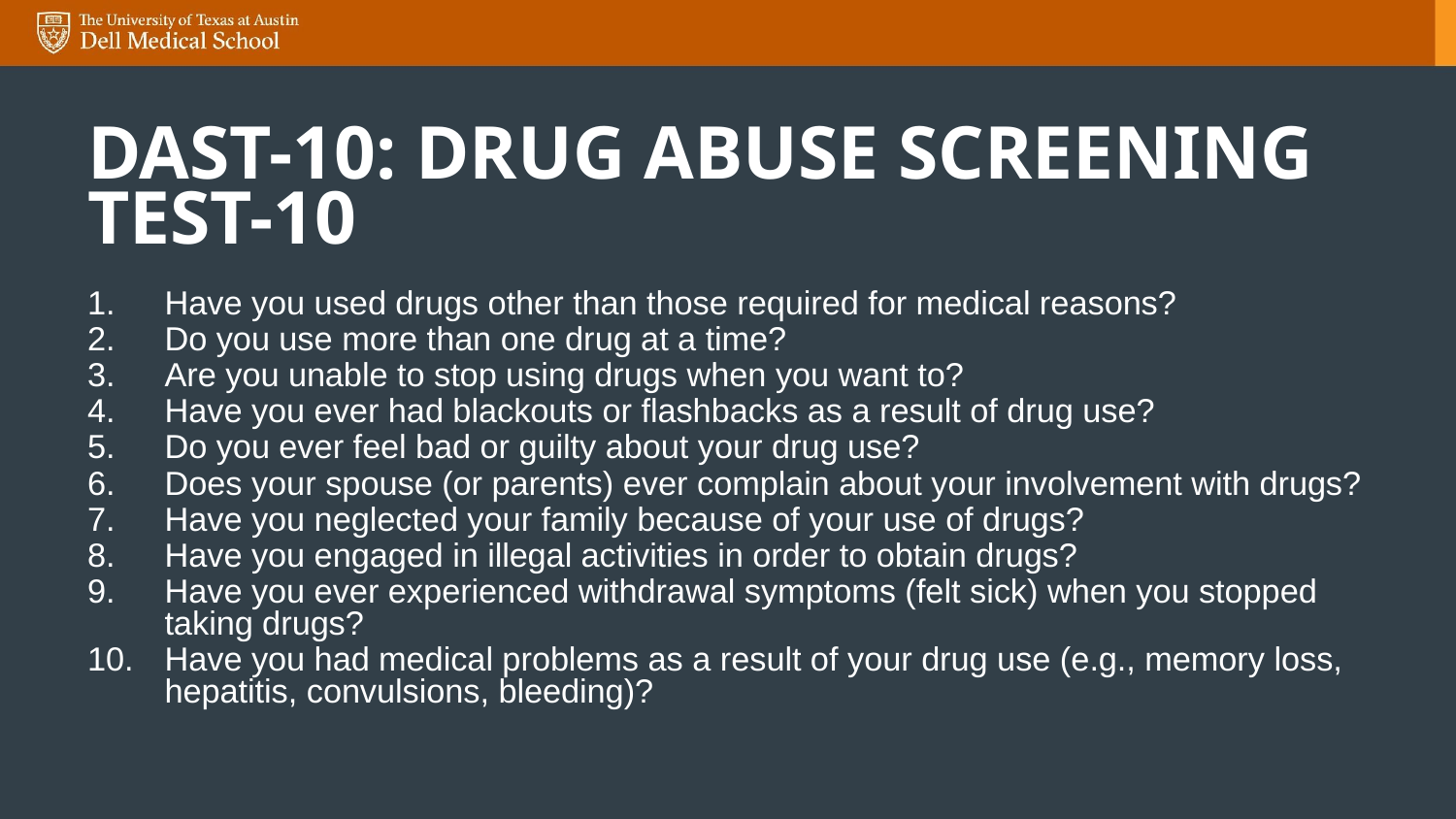

# DAST-10: DRUG ABUSE SCREENING TEST-10
Have you used drugs other than those required for medical reasons?
Do you use more than one drug at a time?
Are you unable to stop using drugs when you want to?
Have you ever had blackouts or flashbacks as a result of drug use?
Do you ever feel bad or guilty about your drug use?
Does your spouse (or parents) ever complain about your involvement with drugs?
Have you neglected your family because of your use of drugs?
Have you engaged in illegal activities in order to obtain drugs?
Have you ever experienced withdrawal symptoms (felt sick) when you stopped taking drugs?
Have you had medical problems as a result of your drug use (e.g., memory loss, hepatitis, convulsions, bleeding)?

## Slide 15
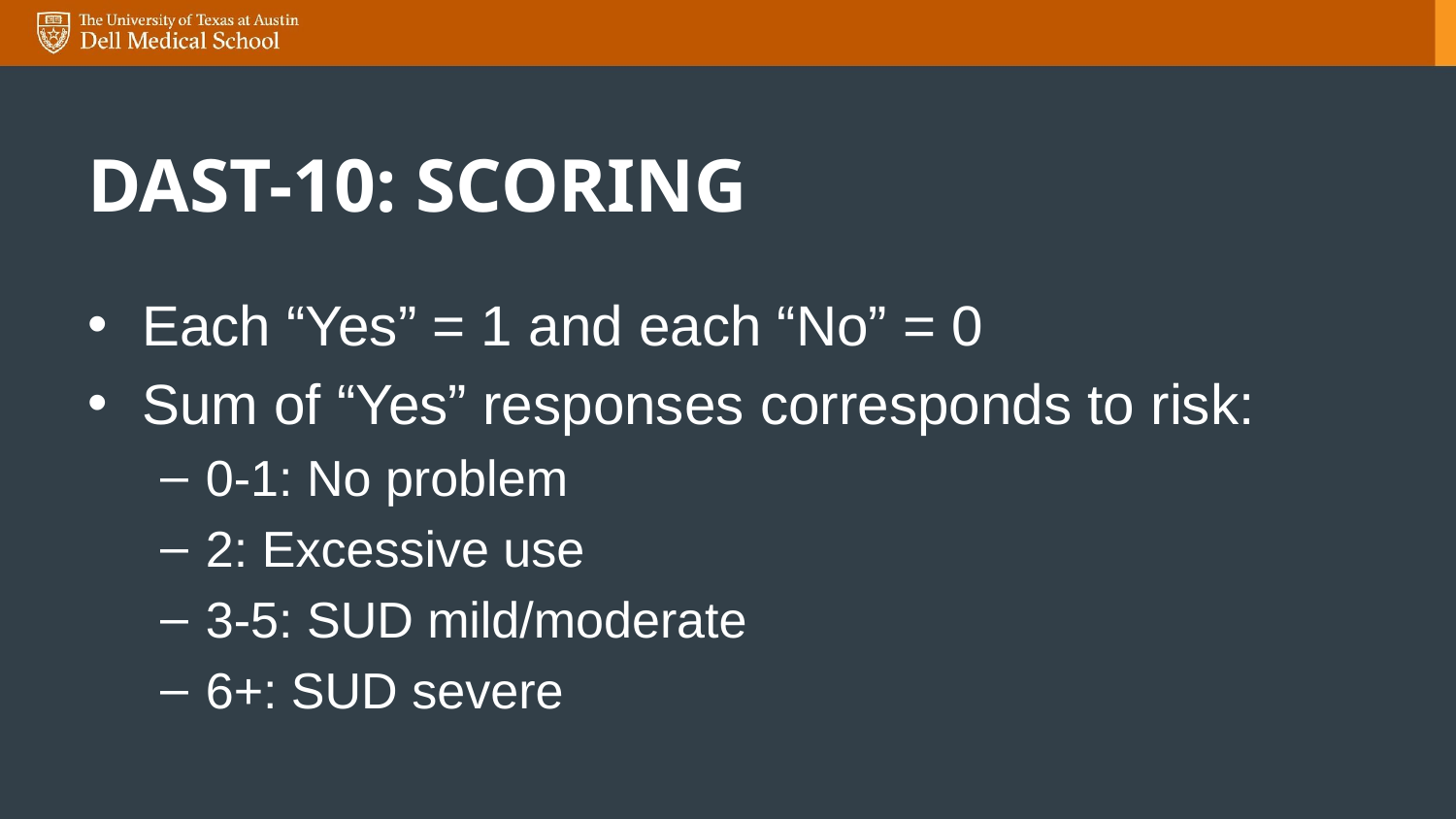

# DAST-10: SCORING
Each “Yes” = 1 and each “No” = 0
Sum of “Yes” responses corresponds to risk:
0-1: No problem
2: Excessive use
3-5: SUD mild/moderate
6+: SUD severe

## Slide 16
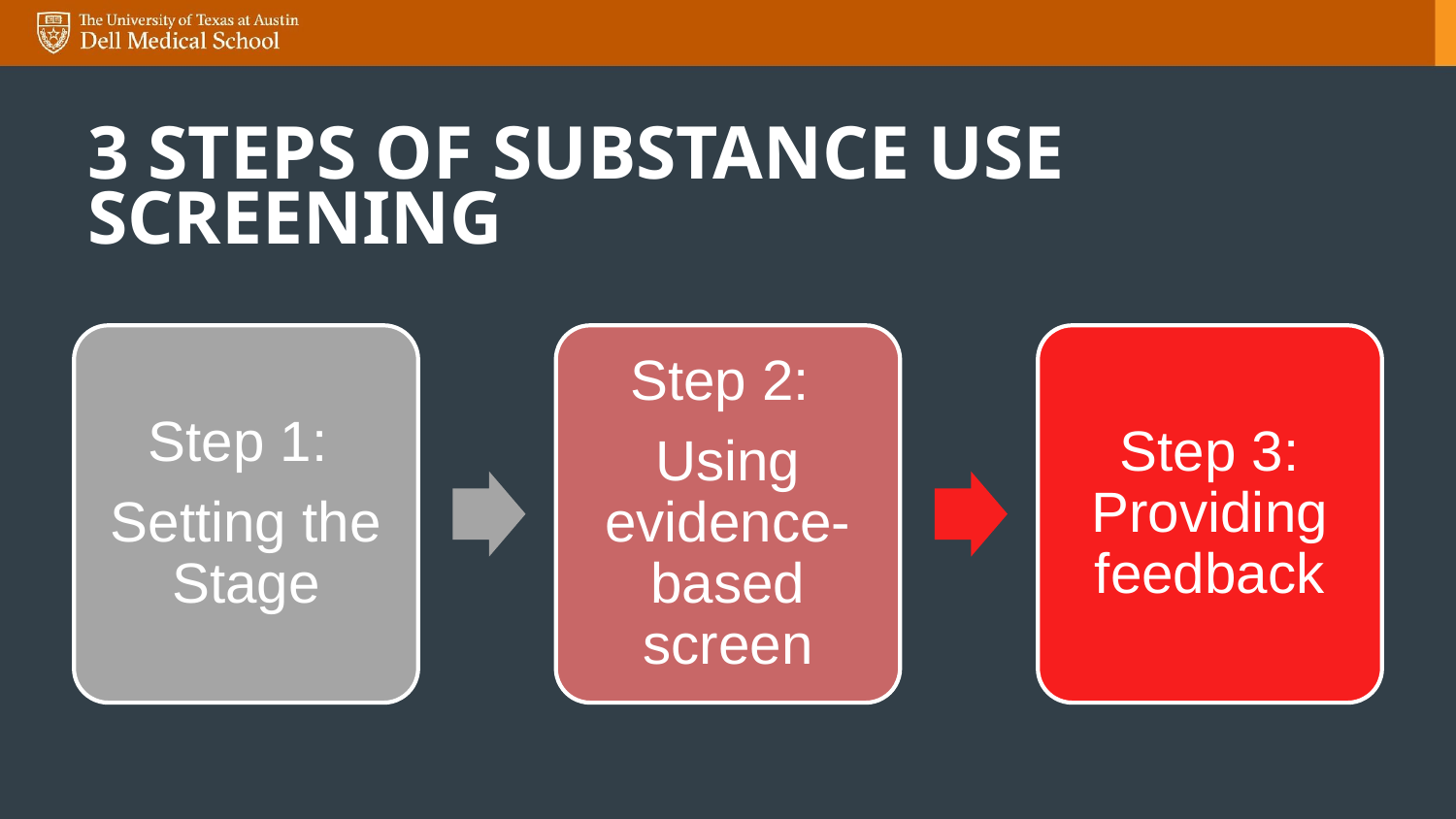

# 3 STEPS OF SUBSTANCE USE SCREENING
Step 1:
Setting the Stage
Step 2:
Using evidence- based screen
Step 3: Providing feedback

## Slide 17
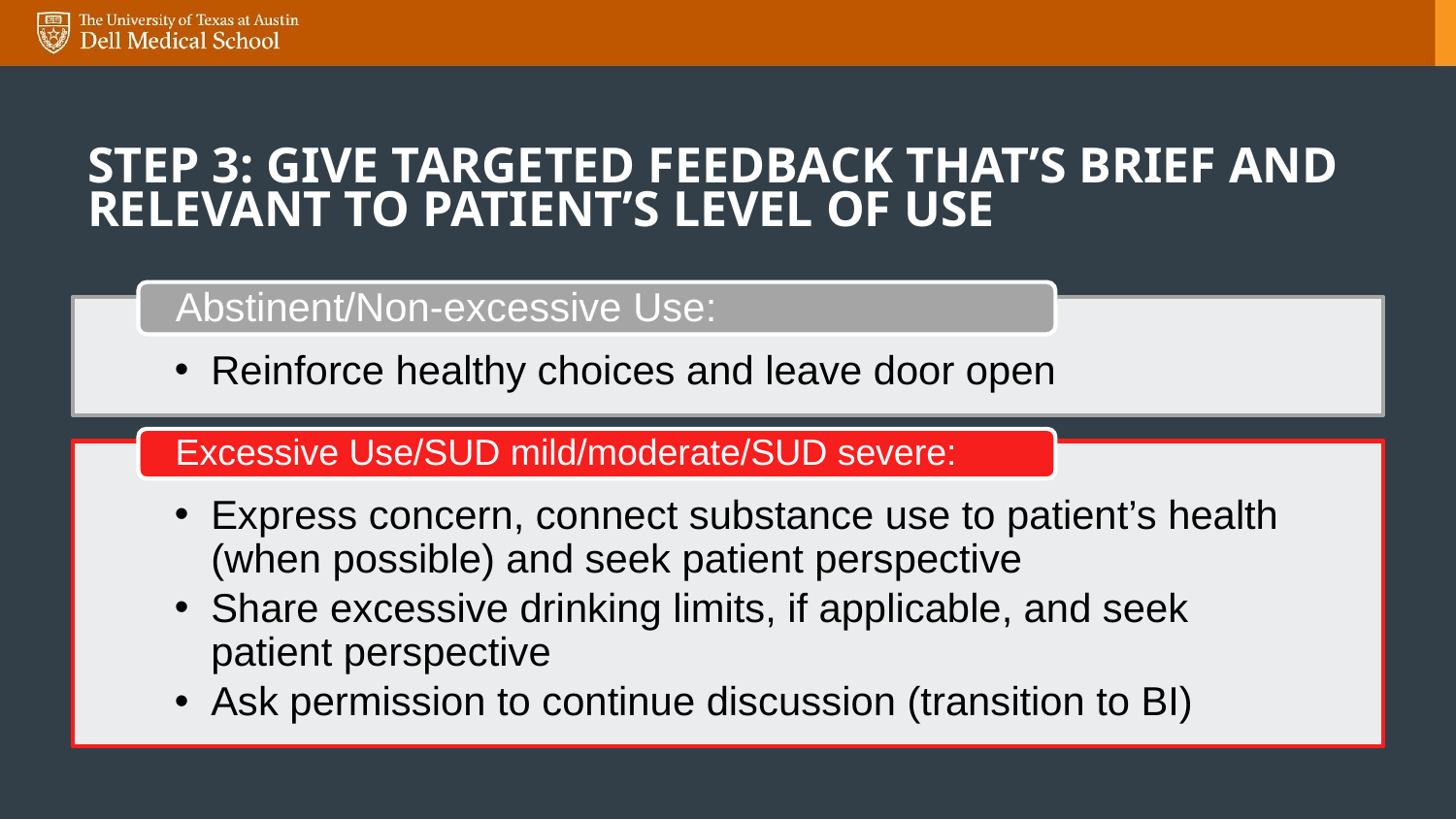

# STEP 3: GIVE TARGETED FEEDBACK THAT’S BRIEF AND RELEVANT TO PATIENT’S LEVEL OF USE
Abstinent/Non-excessive Use:
Reinforce healthy choices and leave door open
Excessive Use/SUD mild/moderate/SUD severe:
Express concern, connect substance use to patient’s health (when possible) and seek patient perspective
Share excessive drinking limits, if applicable, and seek patient perspective
Ask permission to continue discussion (transition to BI)

## Slide 18
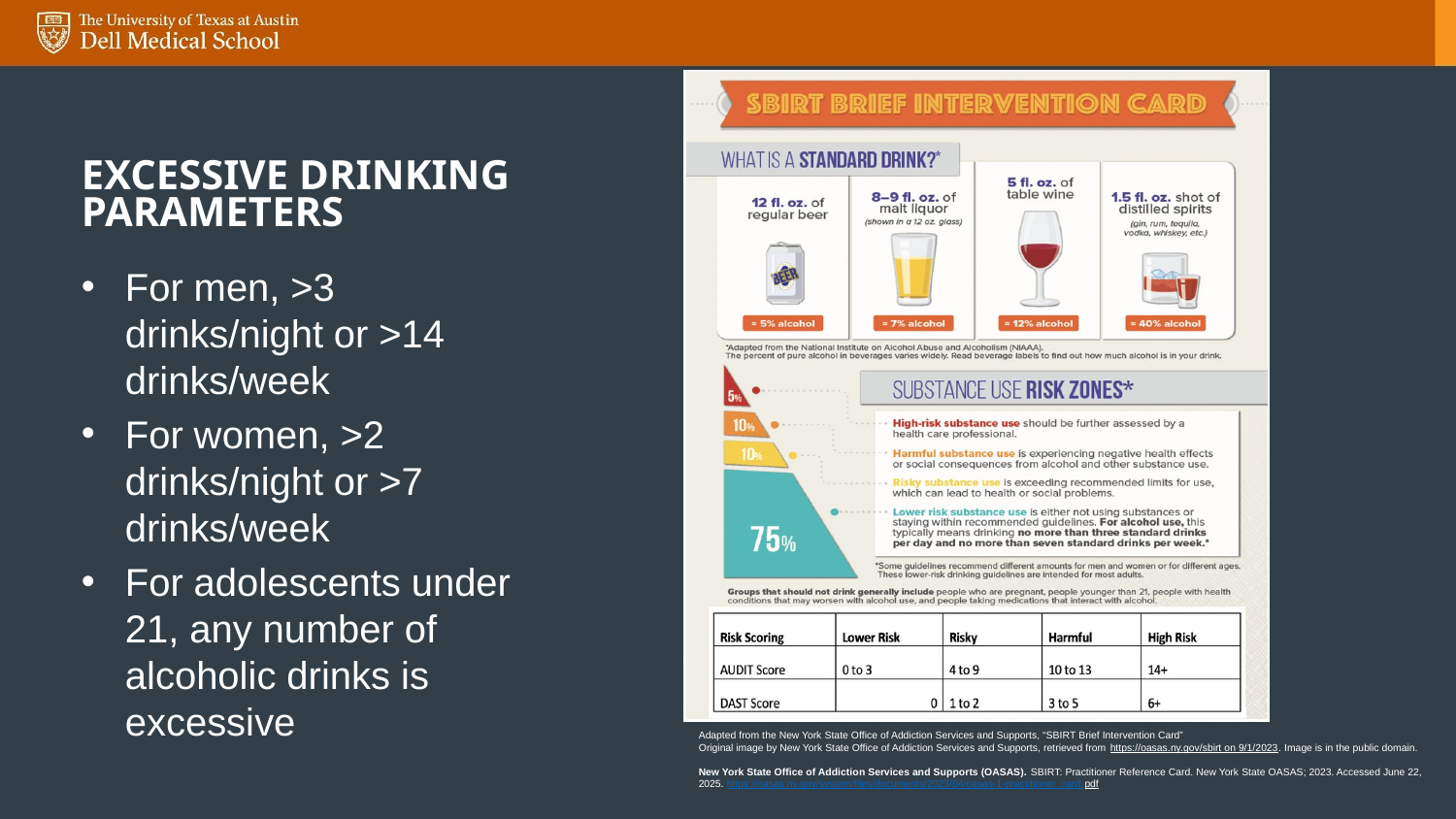

# EXCESSIVE DRINKING PARAMETERS
For men, >3 drinks/night or >14 drinks/week
For women, >2 drinks/night or >7 drinks/week
For adolescents under 21, any number of alcoholic drinks is excessive
Adapted from the New York State Office of Addiction Services and Supports, “SBIRT Brief Intervention Card”
Original image by New York State Office of Addiction Services and Supports, retrieved from https://oasas.ny.gov/sbirt on 9/1/2023. Image is in the public domain.
New York State Office of Addiction Services and Supports (OASAS). SBIRT: Practitioner Reference Card. New York State OASAS; 2023. Accessed June 22, 2025. https://oasas.ny.gov/system/files/documents/2023/04/oasas-1-practitioner_card.pdf

## Slide 19
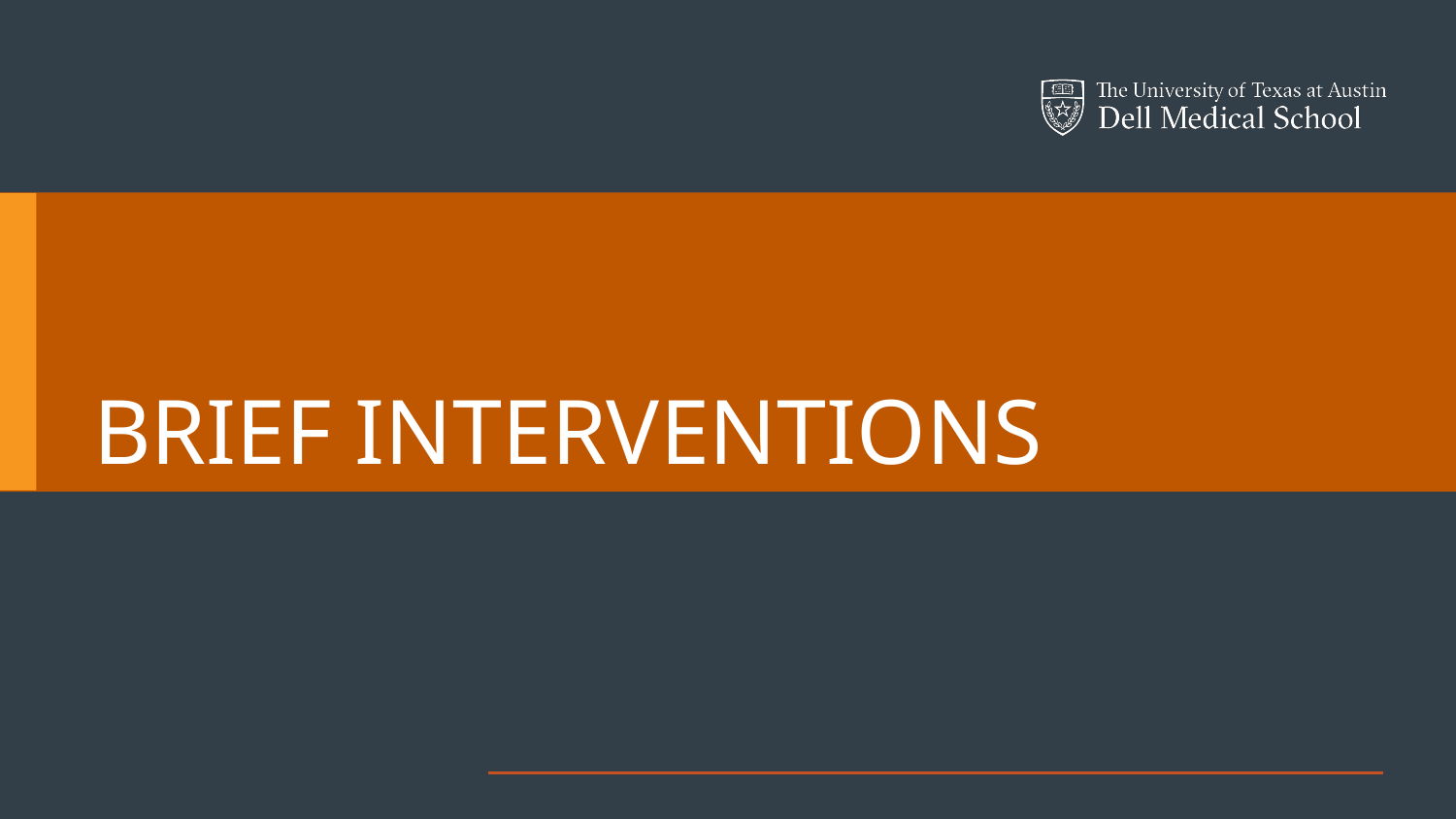

# BRIEF INTERVENTIONS

## Slide 20
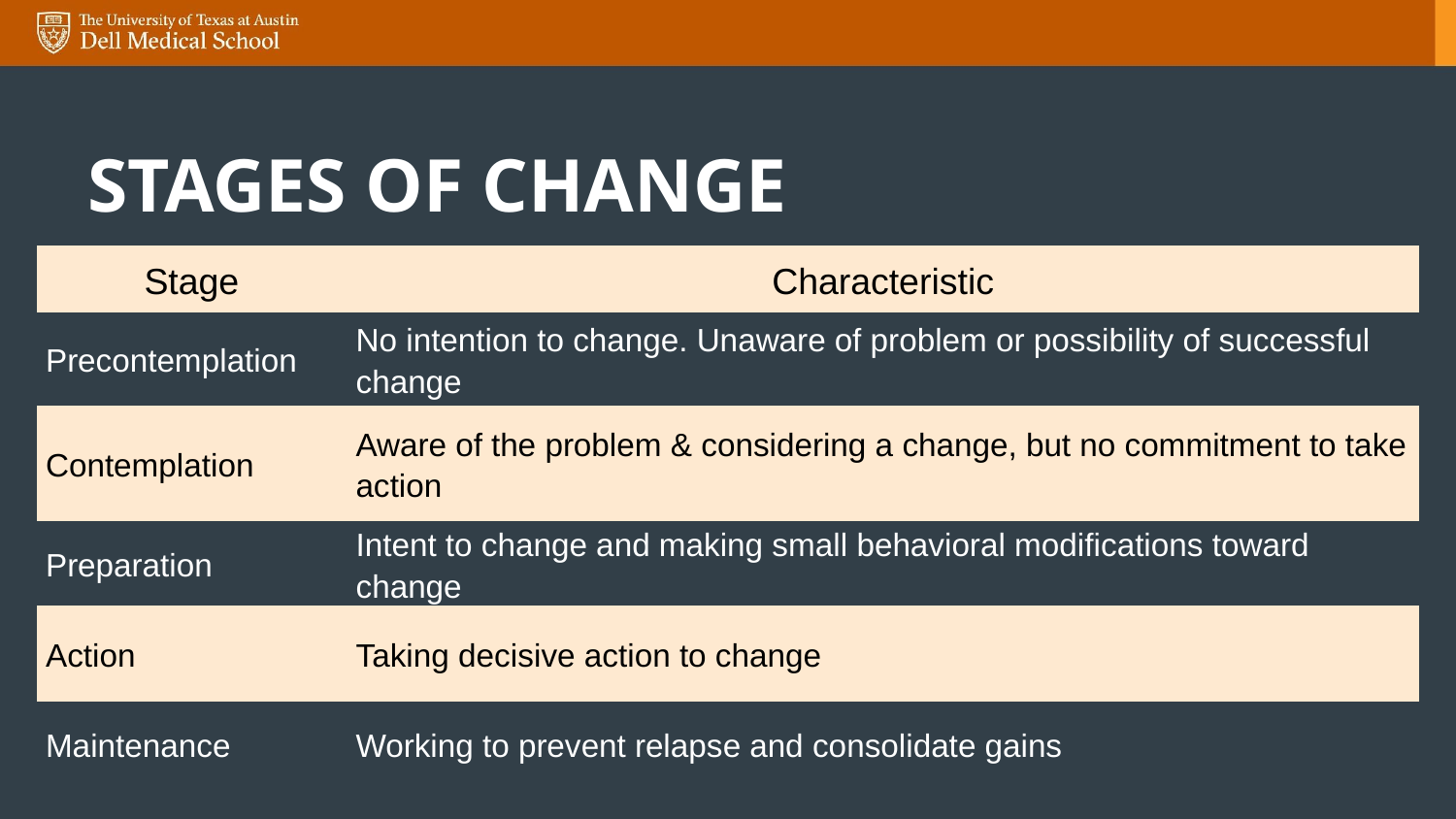

# STAGES OF CHANGE
| Stage | Characteristic |
| --- | --- |
| Precontemplation | No intention to change. Unaware of problem or possibility of successful change |
| Contemplation | Aware of the problem & considering a change, but no commitment to take action |
| Preparation | Intent to change and making small behavioral modifications toward change |
| Action | Taking decisive action to change |
| Maintenance | Working to prevent relapse and consolidate gains |

## Slide 21
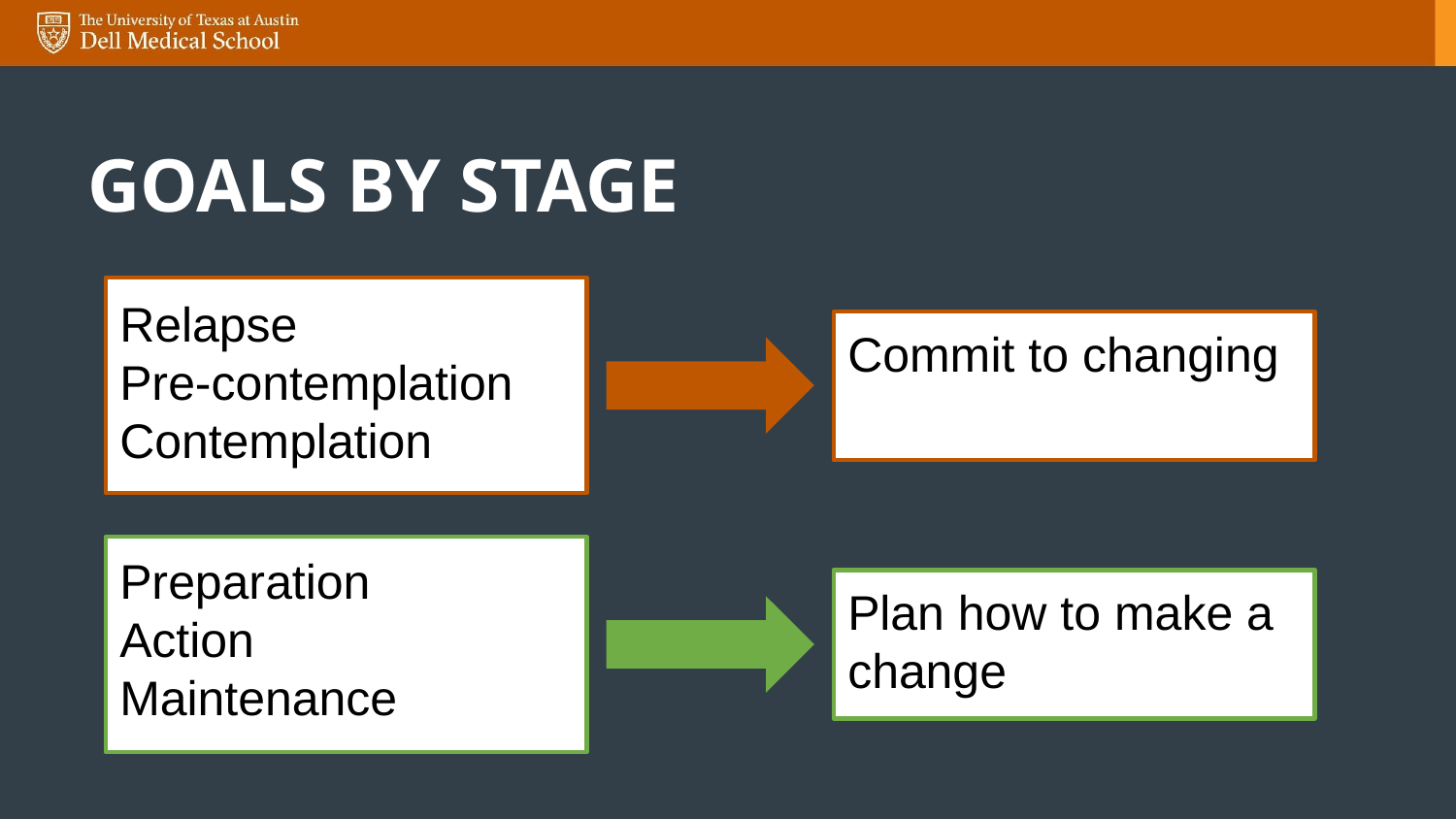

# GOALS BY STAGE
Relapse
Pre-contemplation
Contemplation
Commit to changing
Preparation
Action
Maintenance
Plan how to make a change

## Slide 22
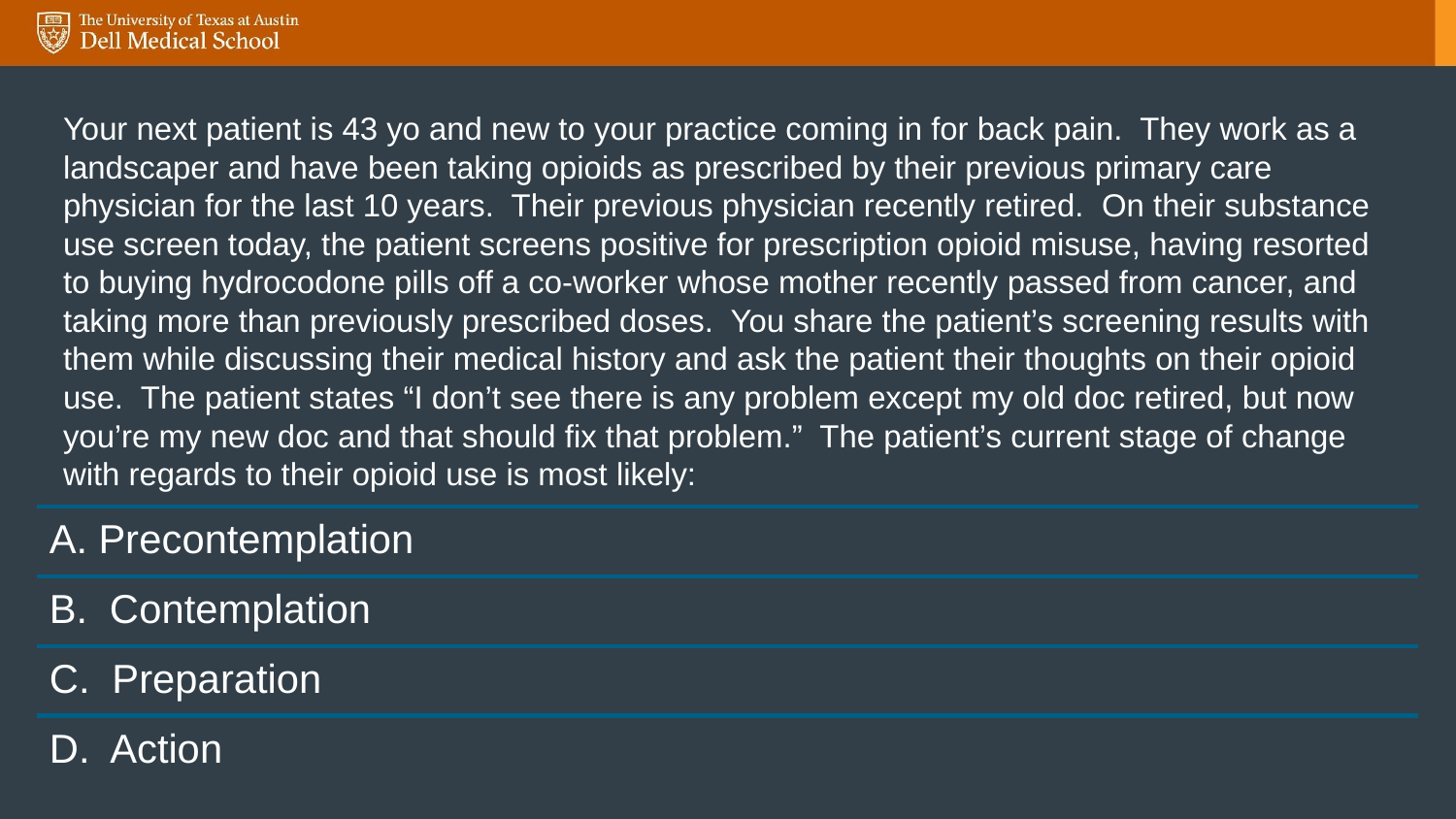

Your next patient is 43 yo and new to your practice coming in for back pain. They work as a landscaper and have been taking opioids as prescribed by their previous primary care physician for the last 10 years. Their previous physician recently retired. On their substance use screen today, the patient screens positive for prescription opioid misuse, having resorted to buying hydrocodone pills off a co-worker whose mother recently passed from cancer, and taking more than previously prescribed doses. You share the patient’s screening results with them while discussing their medical history and ask the patient their thoughts on their opioid use. The patient states “I don’t see there is any problem except my old doc retired, but now you’re my new doc and that should fix that problem.” The patient’s current stage of change with regards to their opioid use is most likely:
A. Precontemplation
B. Contemplation
C. Preparation
D. Action

## Slide 23
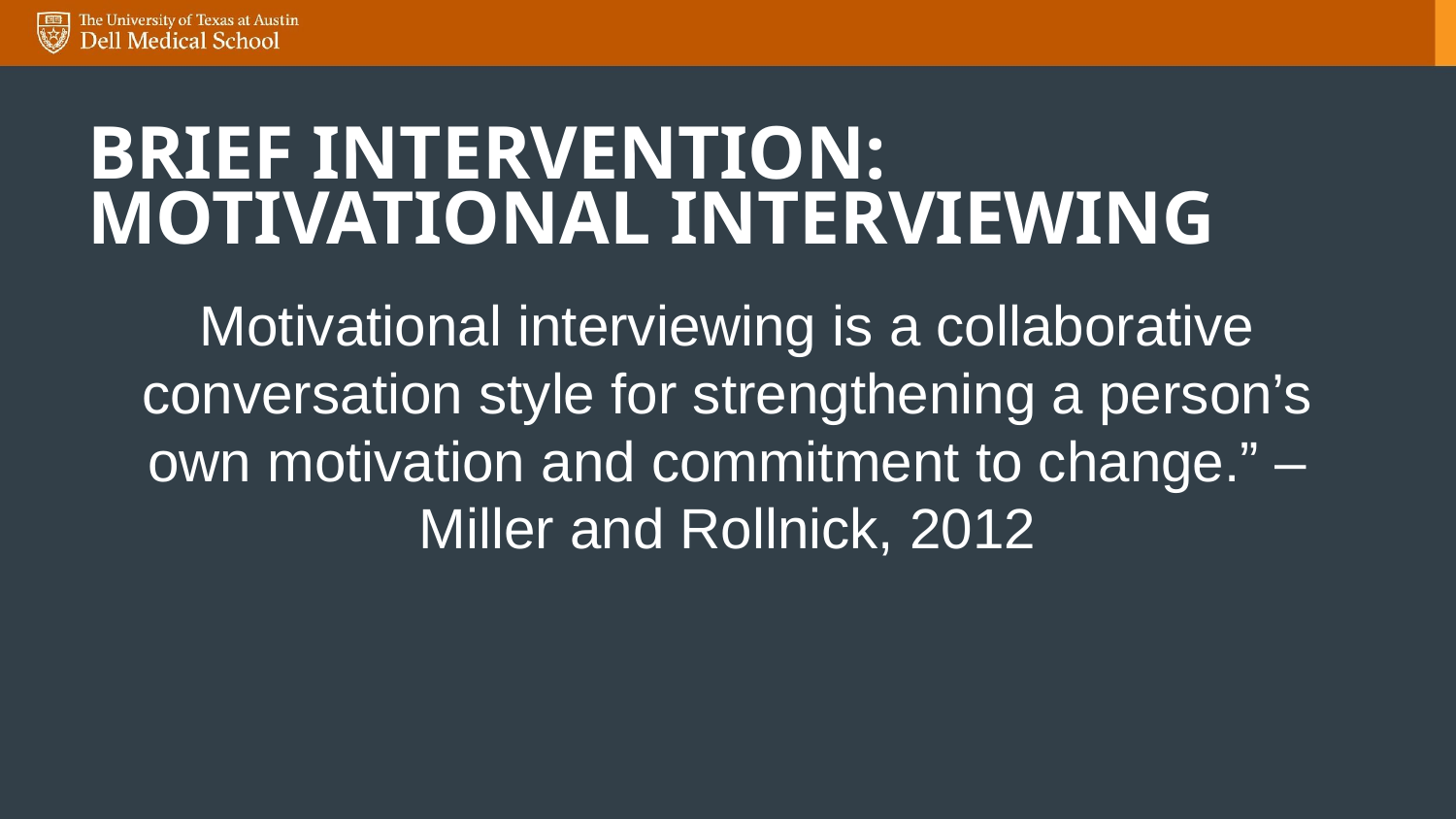

# BRIEF INTERVENTION: MOTIVATIONAL INTERVIEWING
Motivational interviewing is a collaborative conversation style for strengthening a person’s own motivation and commitment to change.” – Miller and Rollnick, 2012

## Slide 24
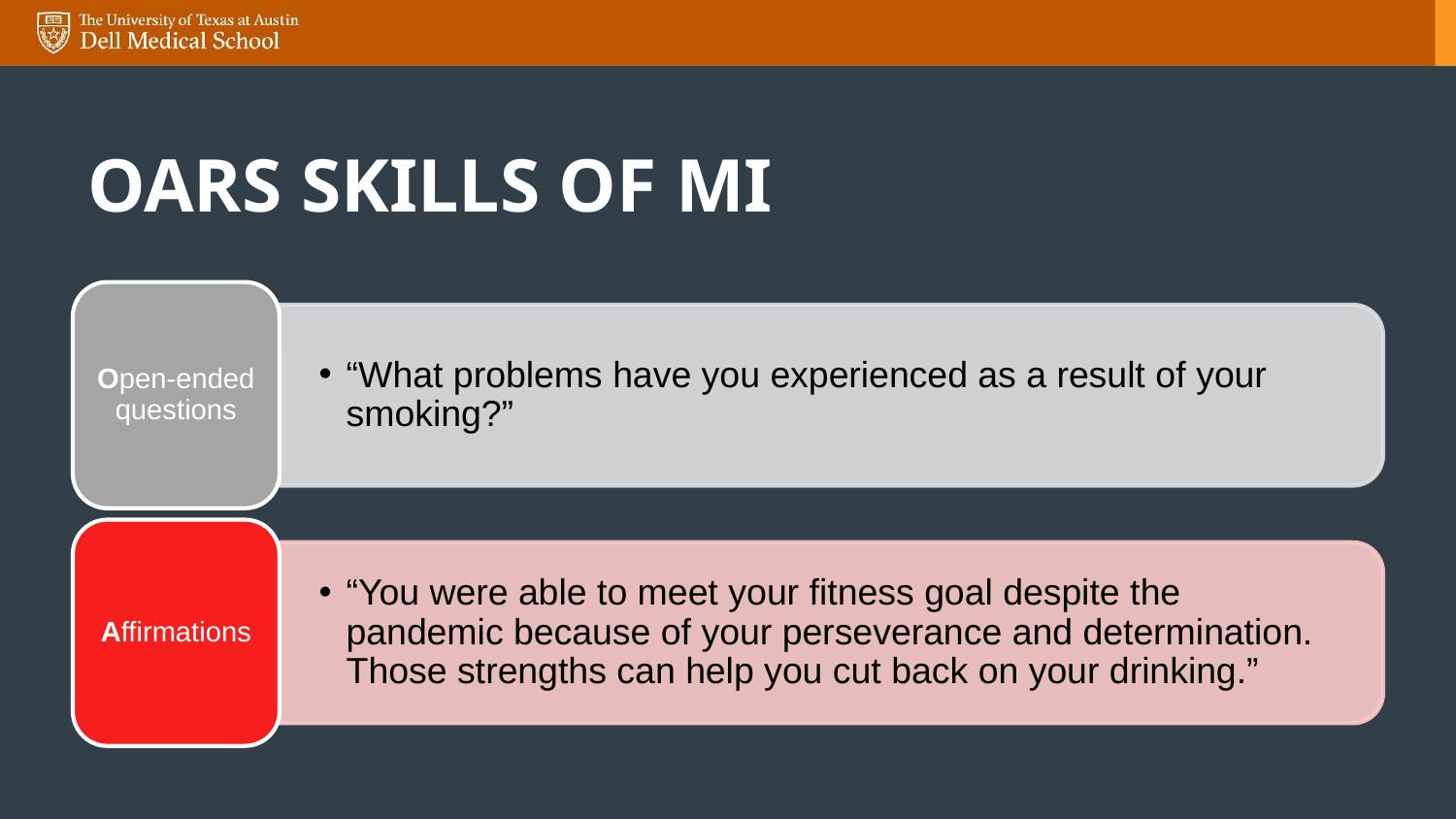

# OARS SKILLS OF MI
Open-ended questions
“What problems have you experienced as a result of your smoking?”
Affirmations
“You were able to meet your fitness goal despite the pandemic because of your perseverance and determination. Those strengths can help you cut back on your drinking.”

## Slide 25
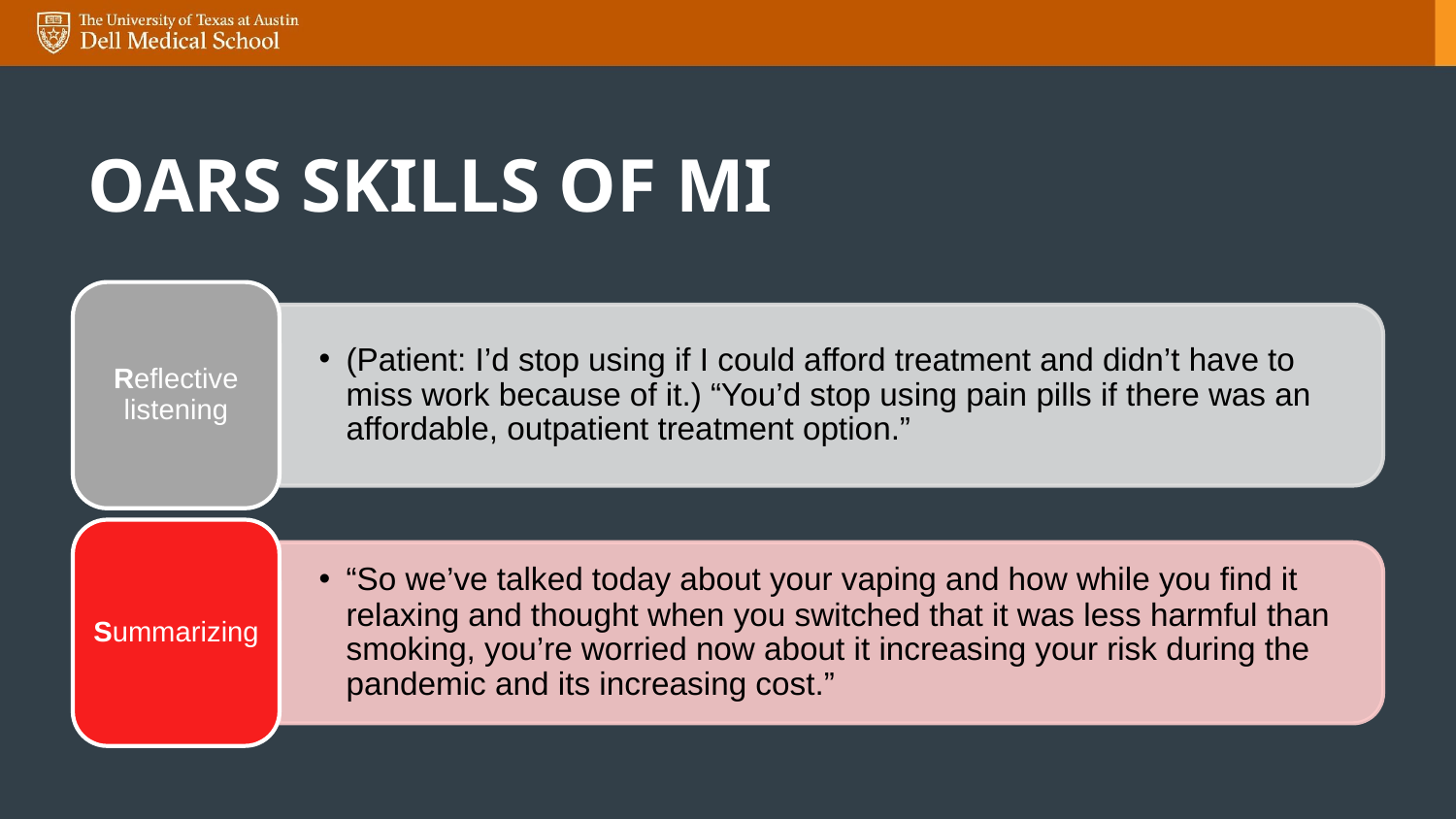

# OARS SKILLS OF MI
Reflective listening
(Patient: I’d stop using if I could afford treatment and didn’t have to miss work because of it.) “You’d stop using pain pills if there was an affordable, outpatient treatment option.”
Summarizing
“So we’ve talked today about your vaping and how while you find it relaxing and thought when you switched that it was less harmful than smoking, you’re worried now about it increasing your risk during the pandemic and its increasing cost.”

## Slide 26
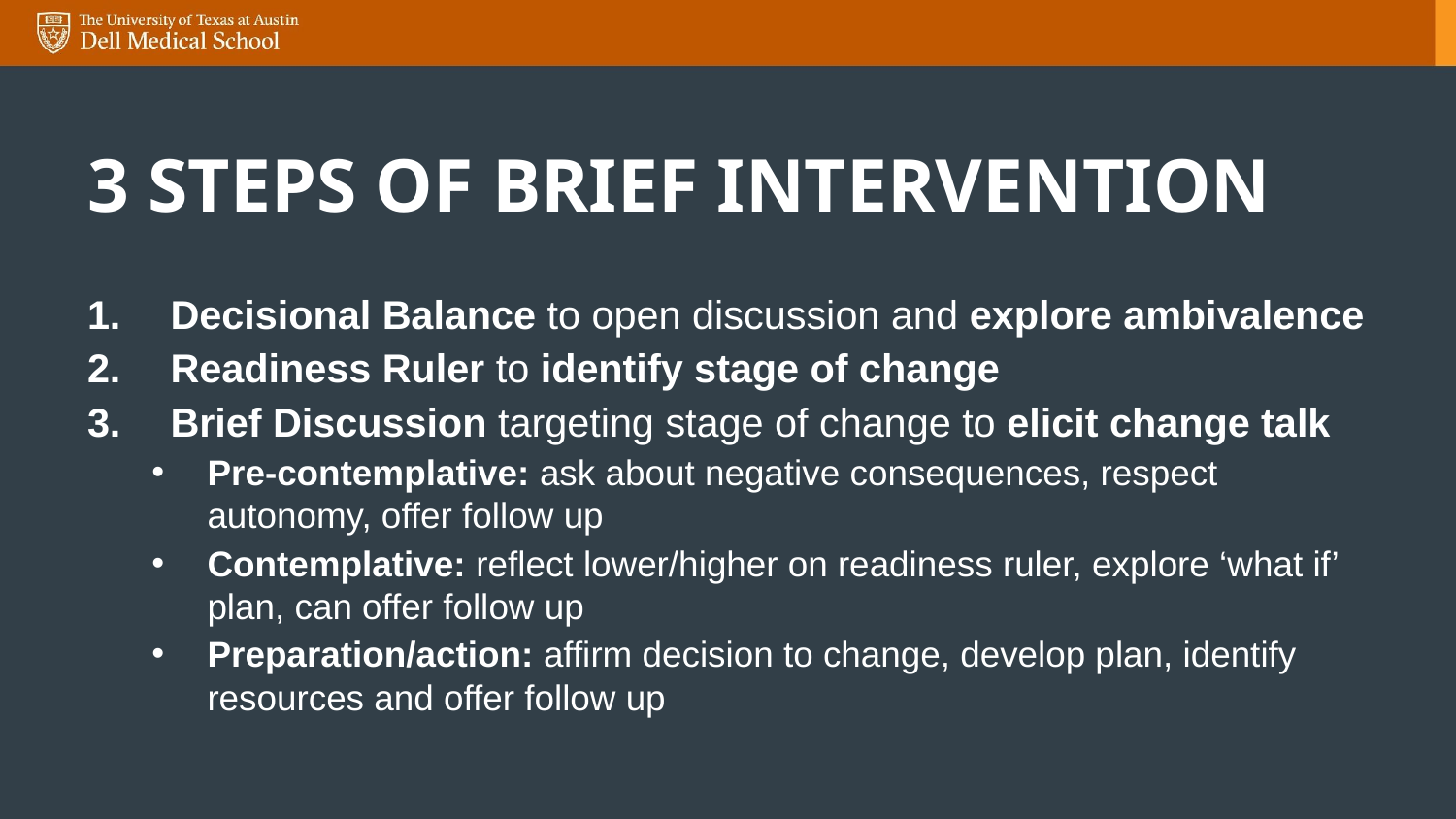

# 3 STEPS OF BRIEF INTERVENTION
Decisional Balance to open discussion and explore ambivalence
Readiness Ruler to identify stage of change
Brief Discussion targeting stage of change to elicit change talk
Pre-contemplative: ask about negative consequences, respect autonomy, offer follow up
Contemplative: reflect lower/higher on readiness ruler, explore ‘what if’ plan, can offer follow up
Preparation/action: affirm decision to change, develop plan, identify resources and offer follow up

## Slide 27
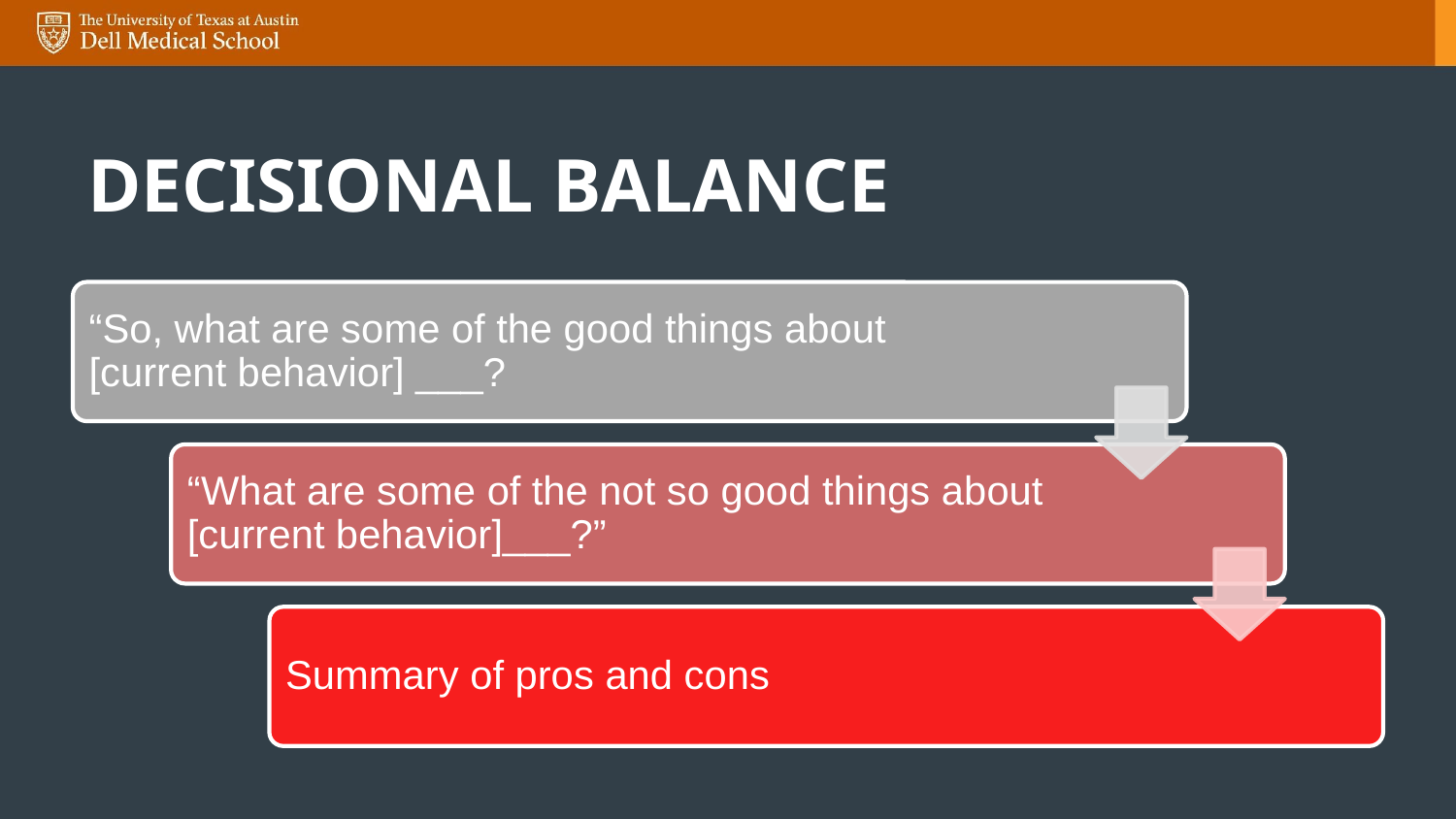

# DECISIONAL BALANCE
“So, what are some of the good things about [current behavior] ___?
“What are some of the not so good things about [current behavior]___?”
Summary of pros and cons

## Slide 28
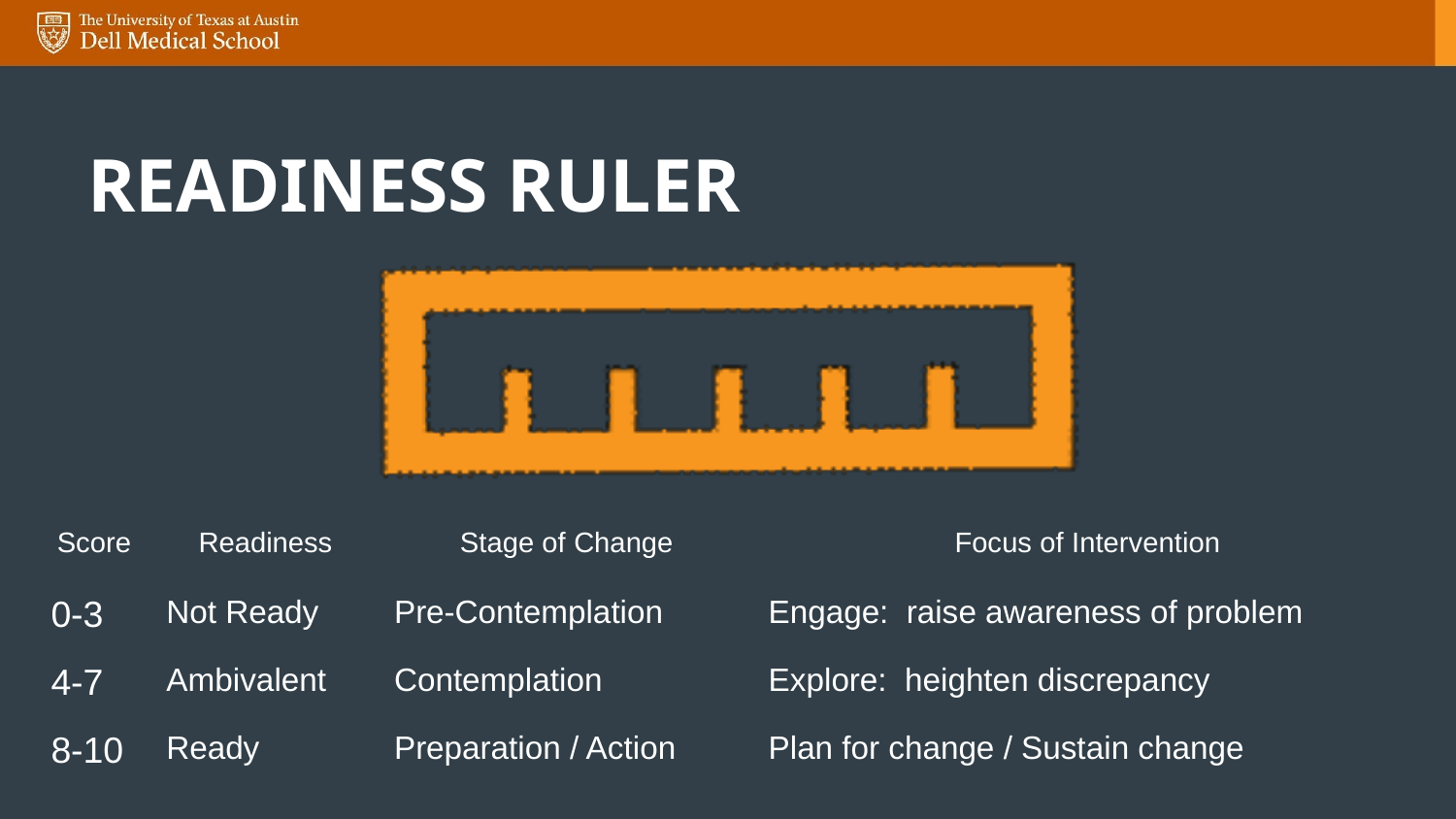

# READINESS RULER
| Score | Readiness | Stage of Change | Focus of Intervention |
| --- | --- | --- | --- |
| 0-3 | Not Ready | Pre-Contemplation | Engage: raise awareness of problem |
| 4-7 | Ambivalent | Contemplation | Explore: heighten discrepancy |
| 8-10 | Ready | Preparation / Action | Plan for change / Sustain change |

## Slide 29
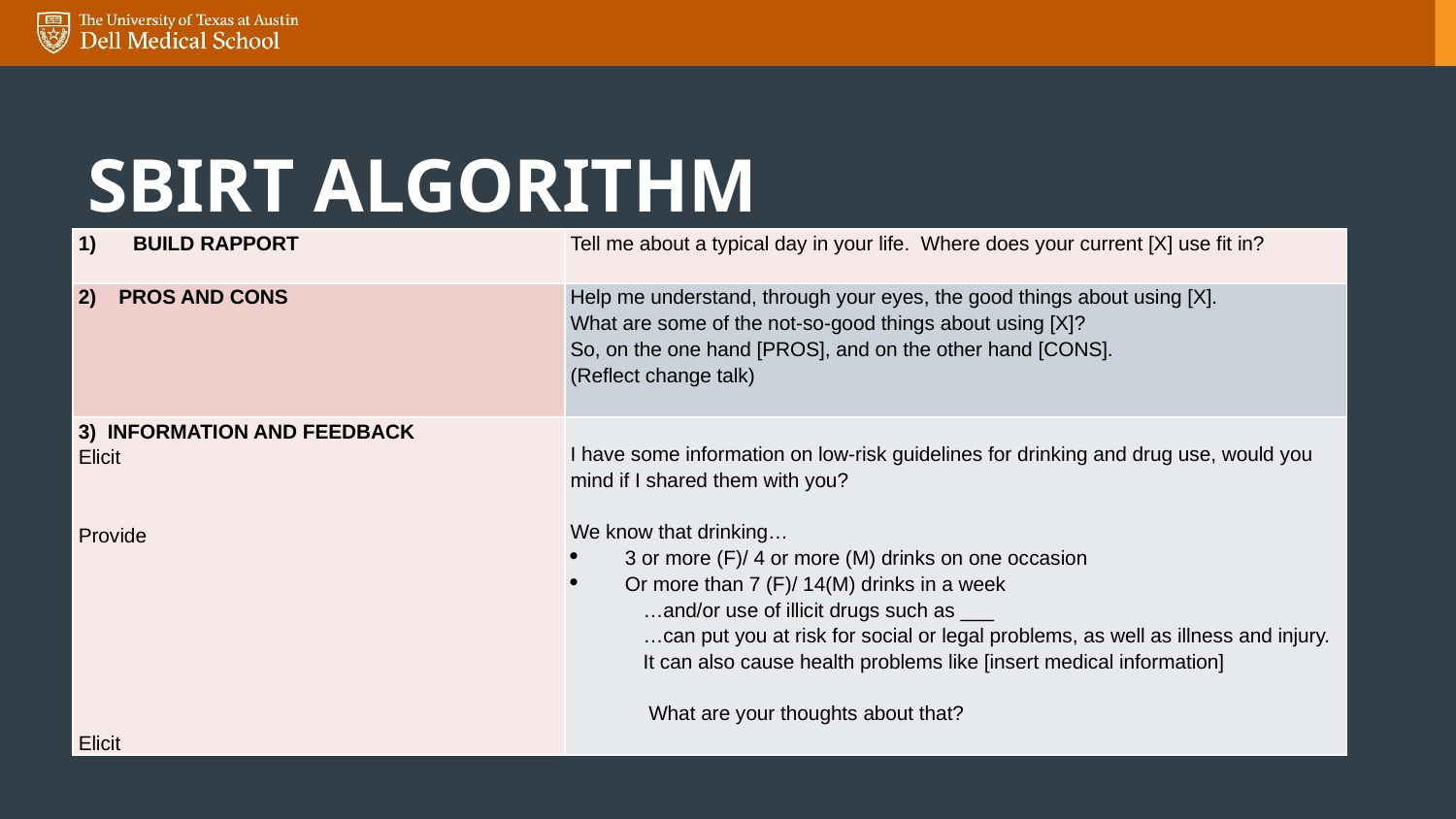

# SBIRT ALGORITHM
| BUILD RAPPORT | Tell me about a typical day in your life. Where does your current [X] use fit in? |
| --- | --- |
| 2) PROS AND CONS | Help me understand, through your eyes, the good things about using [X]. What are some of the not-so-good things about using [X]? So, on the one hand [PROS], and on the other hand [CONS]. (Reflect change talk) |
| 3) INFORMATION AND FEEDBACK Elicit     Provide           Elicit | I have some information on low-risk guidelines for drinking and drug use, would you mind if I shared them with you?   We know that drinking… 3 or more (F)/ 4 or more (M) drinks on one occasion Or more than 7 (F)/ 14(M) drinks in a week …and/or use of illicit drugs such as \_\_\_ …can put you at risk for social or legal problems, as well as illness and injury. It can also cause health problems like [insert medical information]  What are your thoughts about that? |

## Slide 30
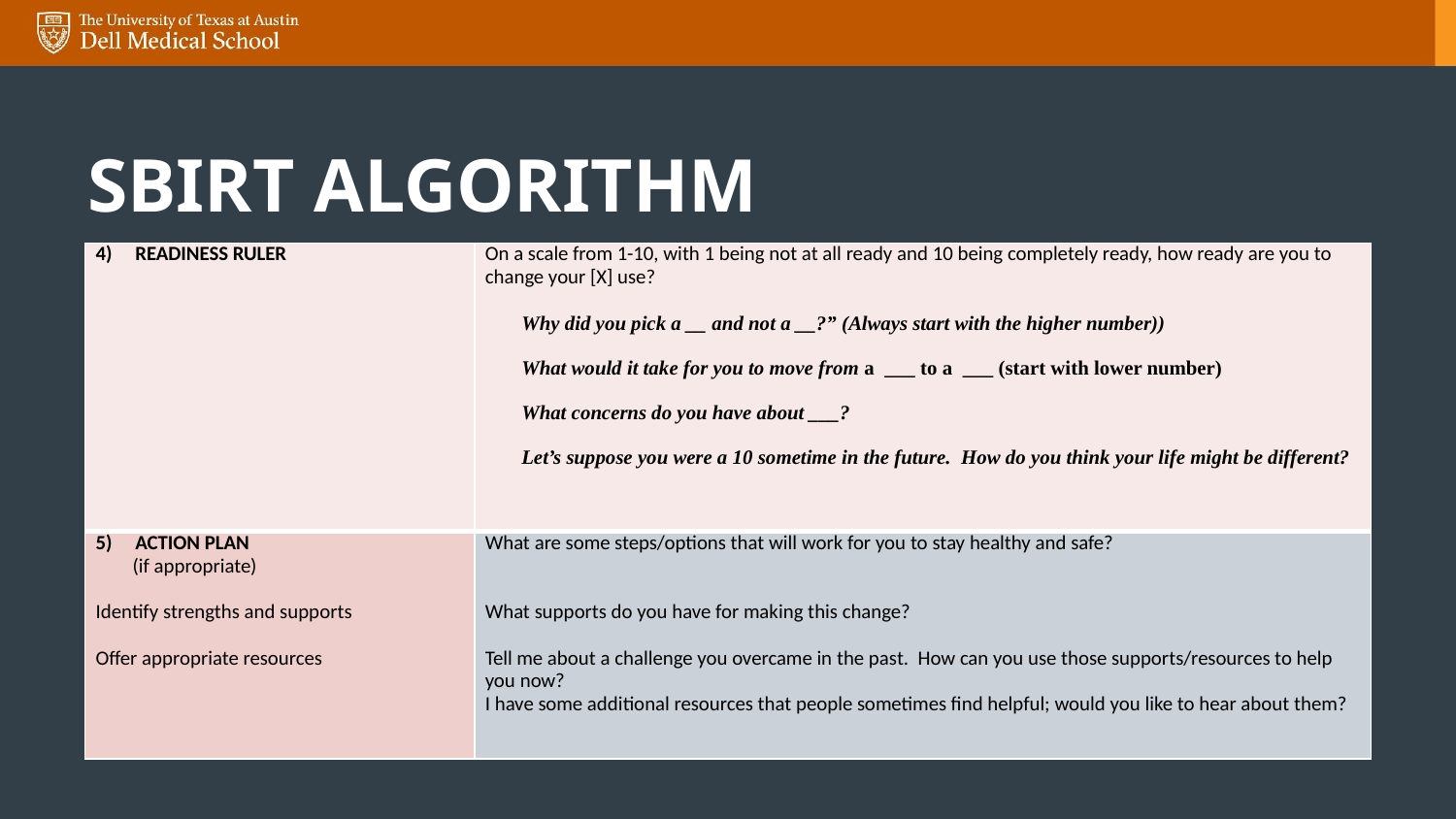

# SBIRT ALGORITHM
| 4) READINESS RULER | On a scale from 1-10, with 1 being not at all ready and 10 being completely ready, how ready are you to change your [X] use?   Why did you pick a \_\_ and not a \_\_?” (Always start with the higher number))   What would it take for you to move from a \_\_\_ to a \_\_\_ (start with lower number)   What concerns do you have about \_\_\_?   Let’s suppose you were a 10 sometime in the future. How do you think your life might be different? |
| --- | --- |
| 5) ACTION PLAN (if appropriate)   Identify strengths and supports   Offer appropriate resources | What are some steps/options that will work for you to stay healthy and safe?   What supports do you have for making this change?   Tell me about a challenge you overcame in the past. How can you use those supports/resources to help you now? I have some additional resources that people sometimes find helpful; would you like to hear about them? |

## Slide 31
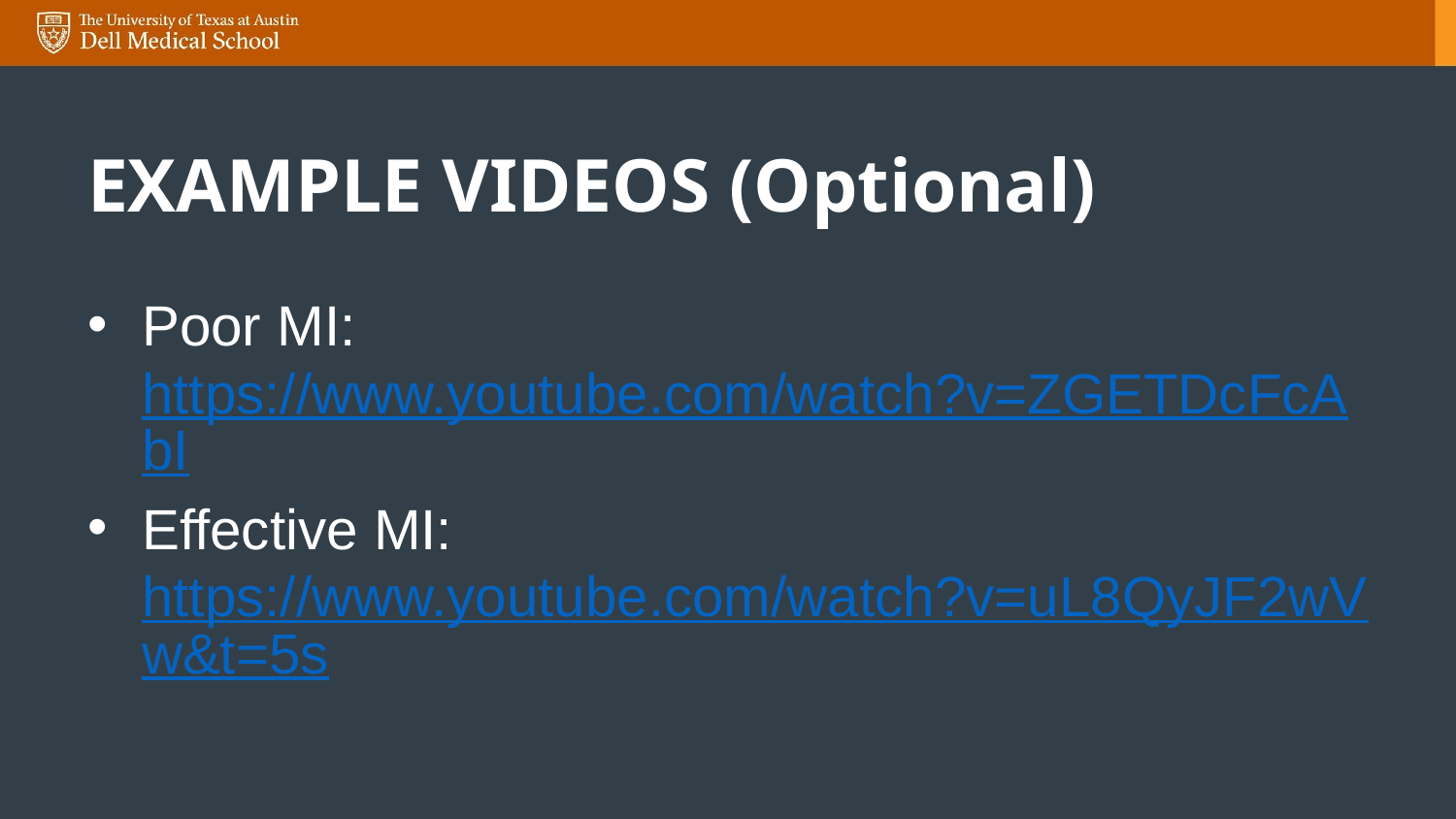

# EXAMPLE VIDEOS (Optional)
Poor MI: https://www.youtube.com/watch?v=ZGETDcFcAbI
Effective MI: https://www.youtube.com/watch?v=uL8QyJF2wVw&t=5s

## Slide 32
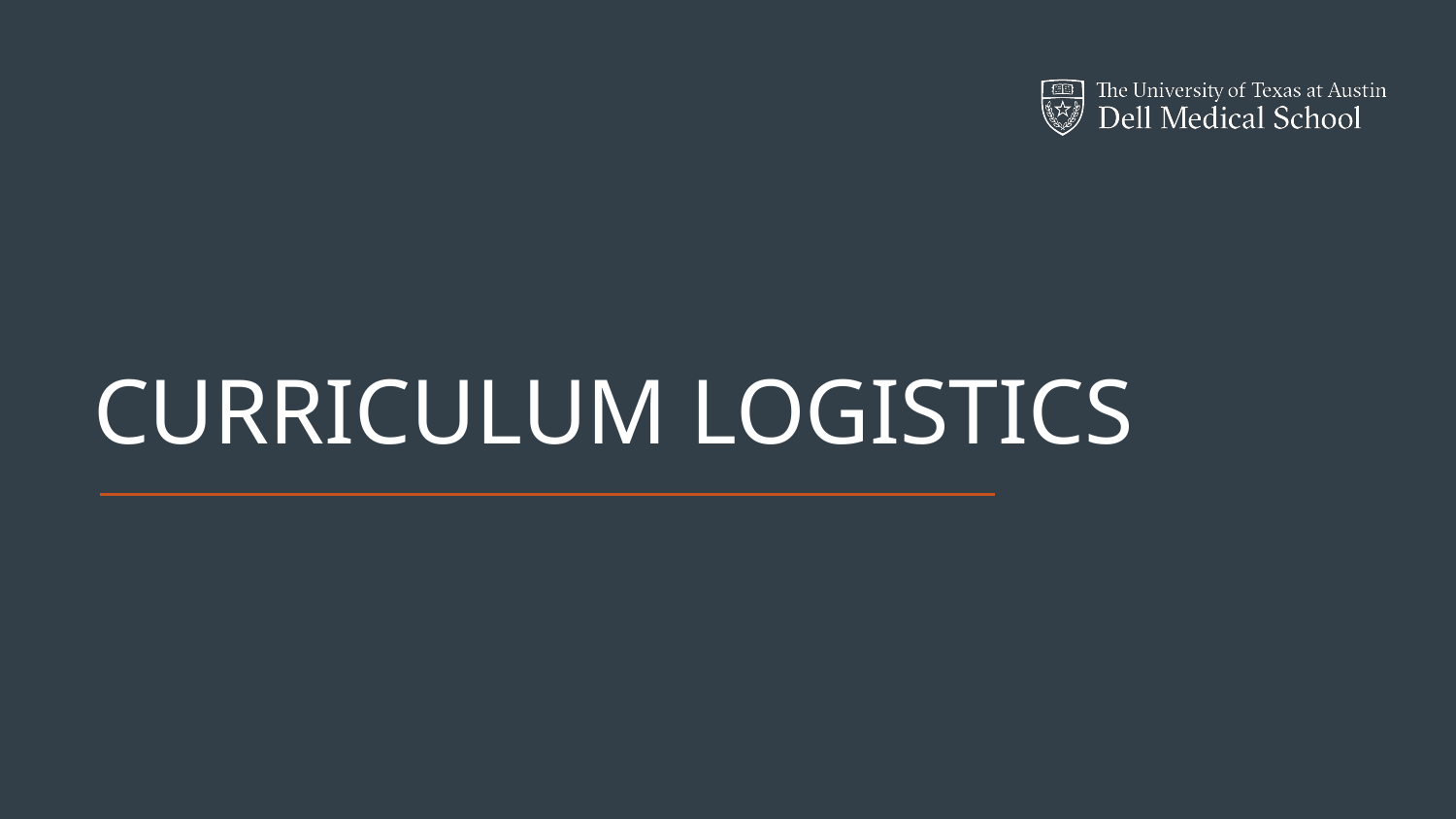

# CURRICULUM LOGISTICS

## Slide 33
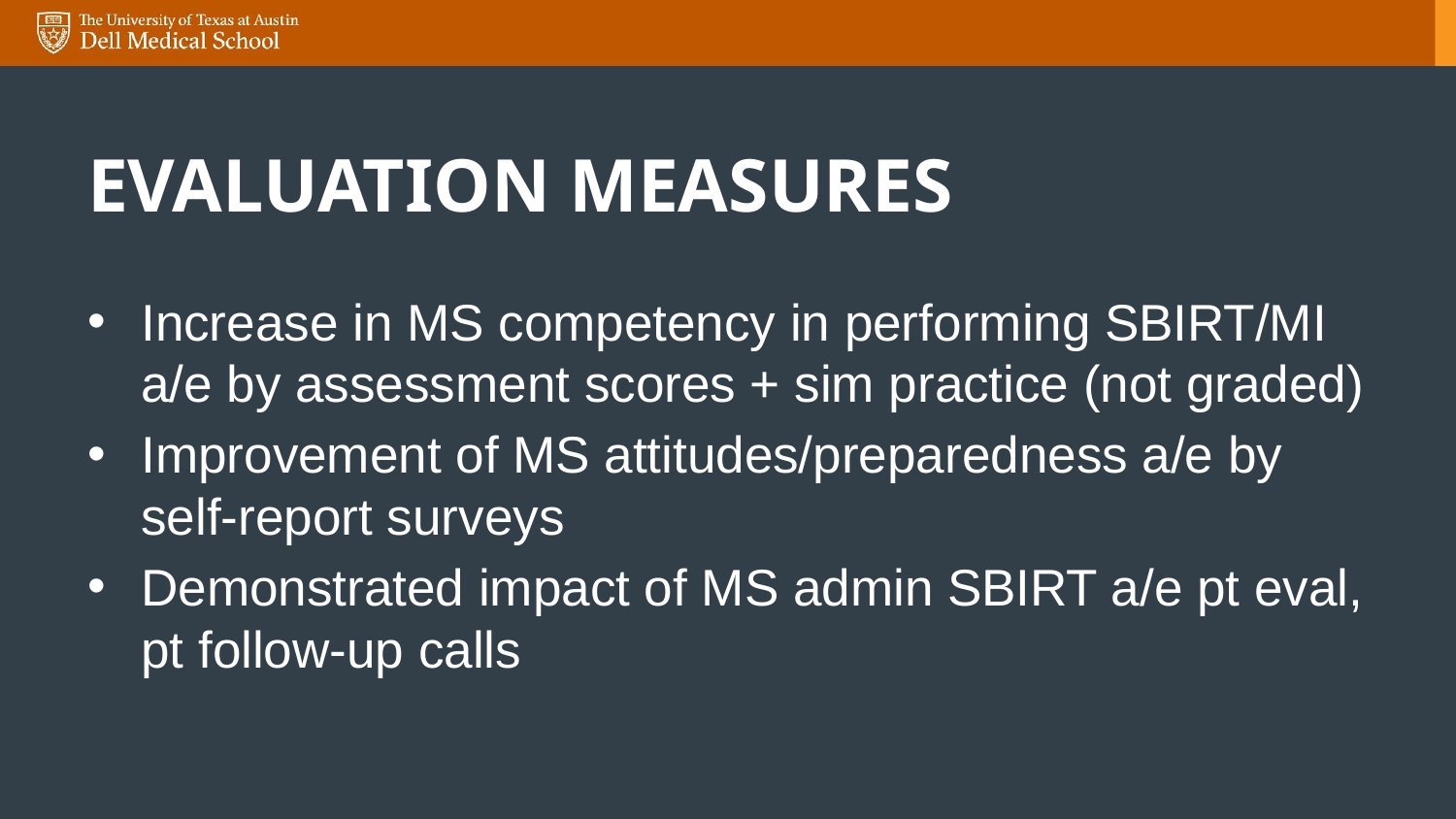

# EVALUATION MEASURES
Increase in MS competency in performing SBIRT/MI a/e by assessment scores + sim practice (not graded)
Improvement of MS attitudes/preparedness a/e by self-report surveys
Demonstrated impact of MS admin SBIRT a/e pt eval, pt follow-up calls

## Slide 34
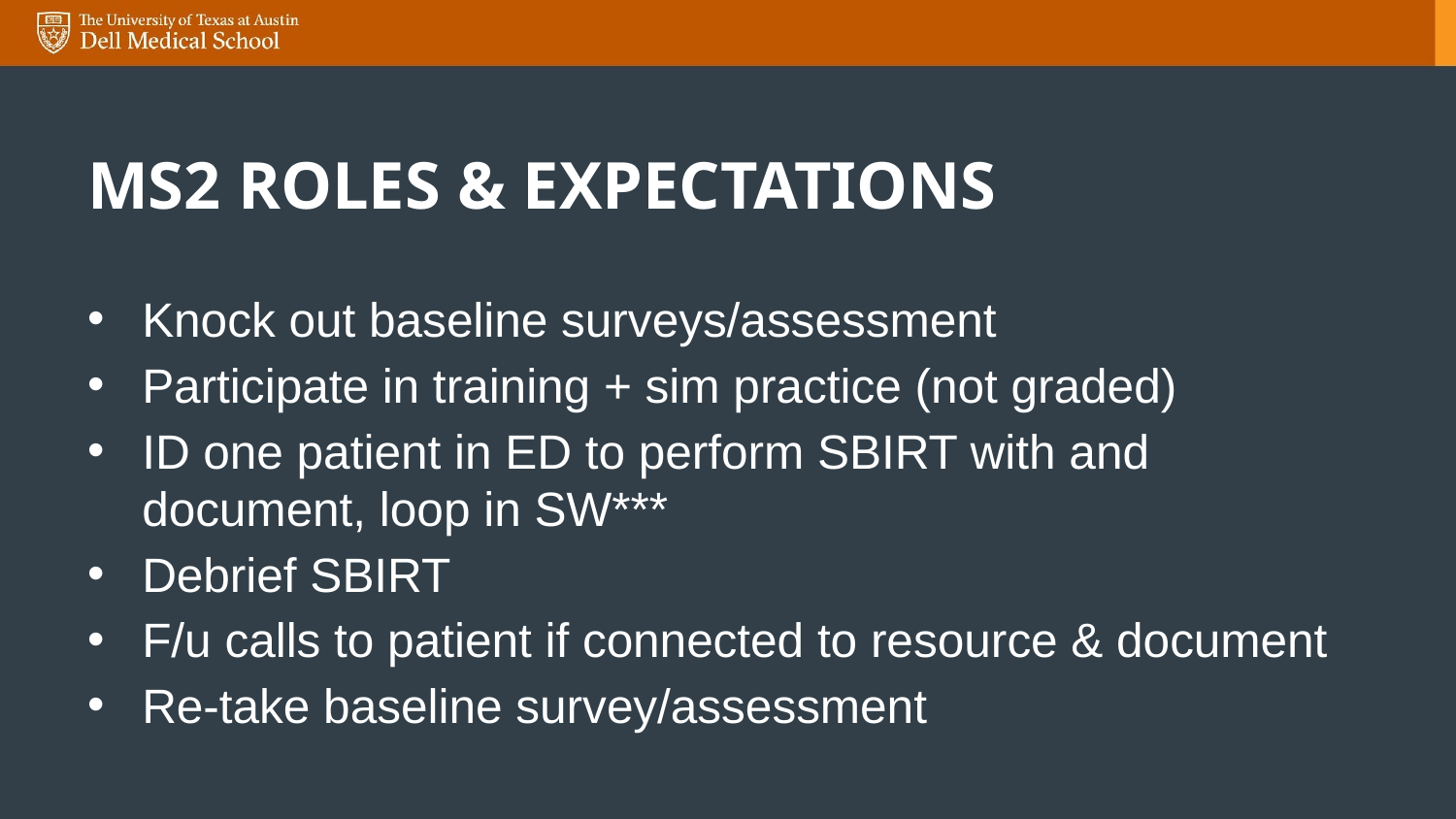

# MS2 ROLES & EXPECTATIONS
Knock out baseline surveys/assessment
Participate in training + sim practice (not graded)
ID one patient in ED to perform SBIRT with and document, loop in SW***
Debrief SBIRT
F/u calls to patient if connected to resource & document
Re-take baseline survey/assessment

## Slide 35
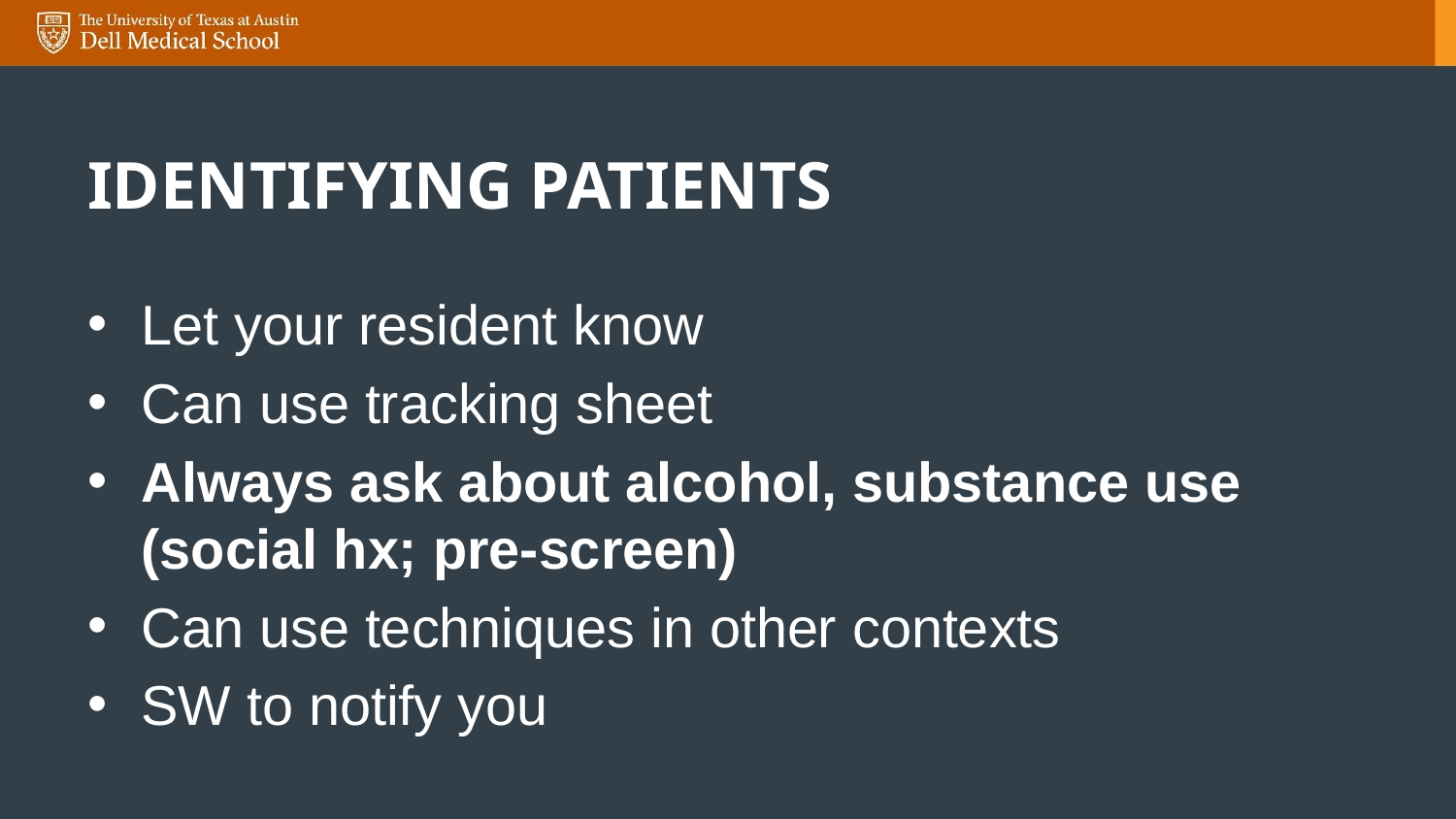

# IDENTIFYING PATIENTS
Let your resident know
Can use tracking sheet
Always ask about alcohol, substance use (social hx; pre-screen)
Can use techniques in other contexts
SW to notify you

## Slide 36
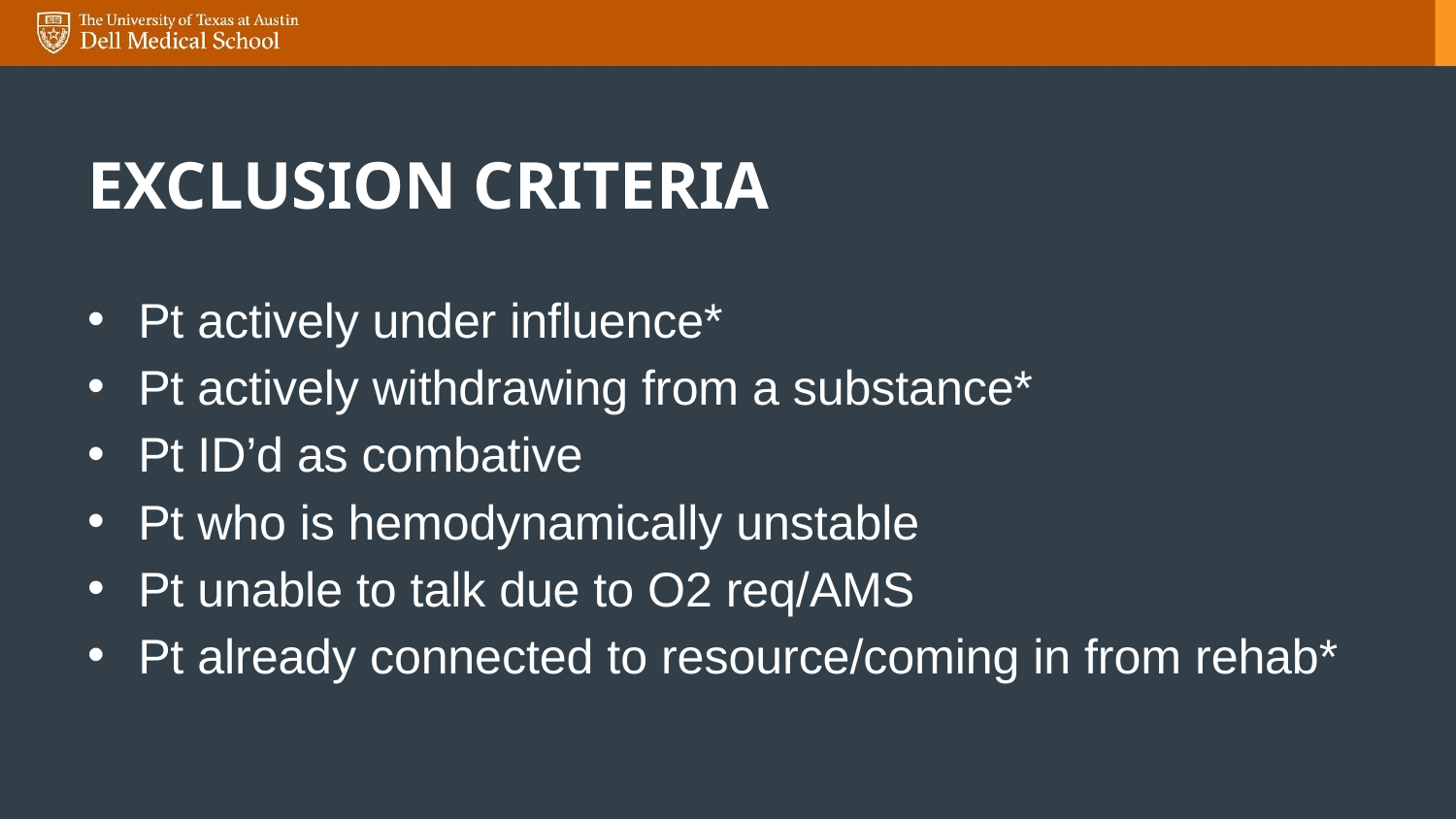

# EXCLUSION CRITERIA
Pt actively under influence*
Pt actively withdrawing from a substance*
Pt ID’d as combative
Pt who is hemodynamically unstable
Pt unable to talk due to O2 req/AMS
Pt already connected to resource/coming in from rehab*

## Slide 37
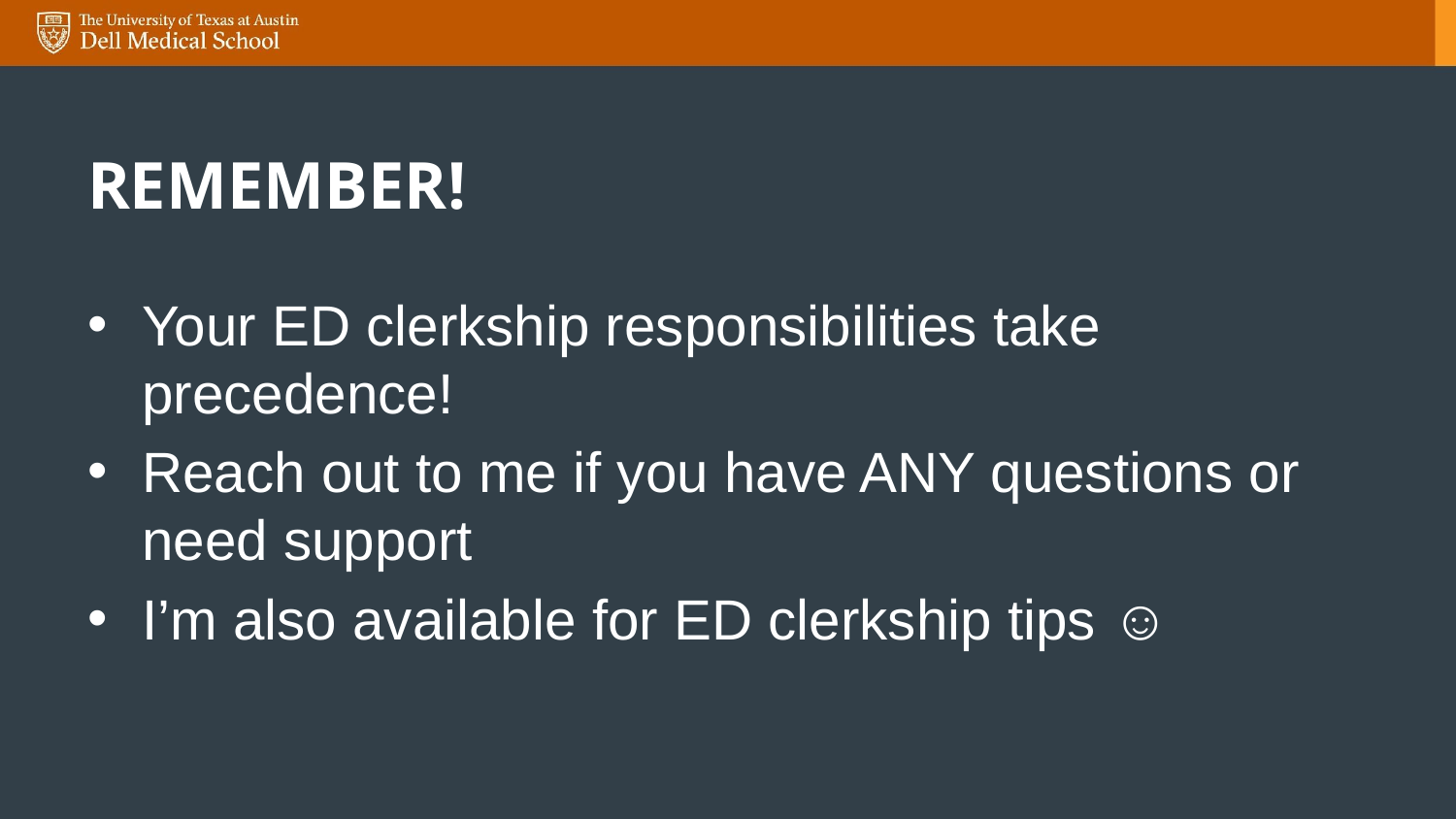

# REMEMBER!
Your ED clerkship responsibilities take precedence!
Reach out to me if you have ANY questions or need support
I’m also available for ED clerkship tips ☺

## Slide 38
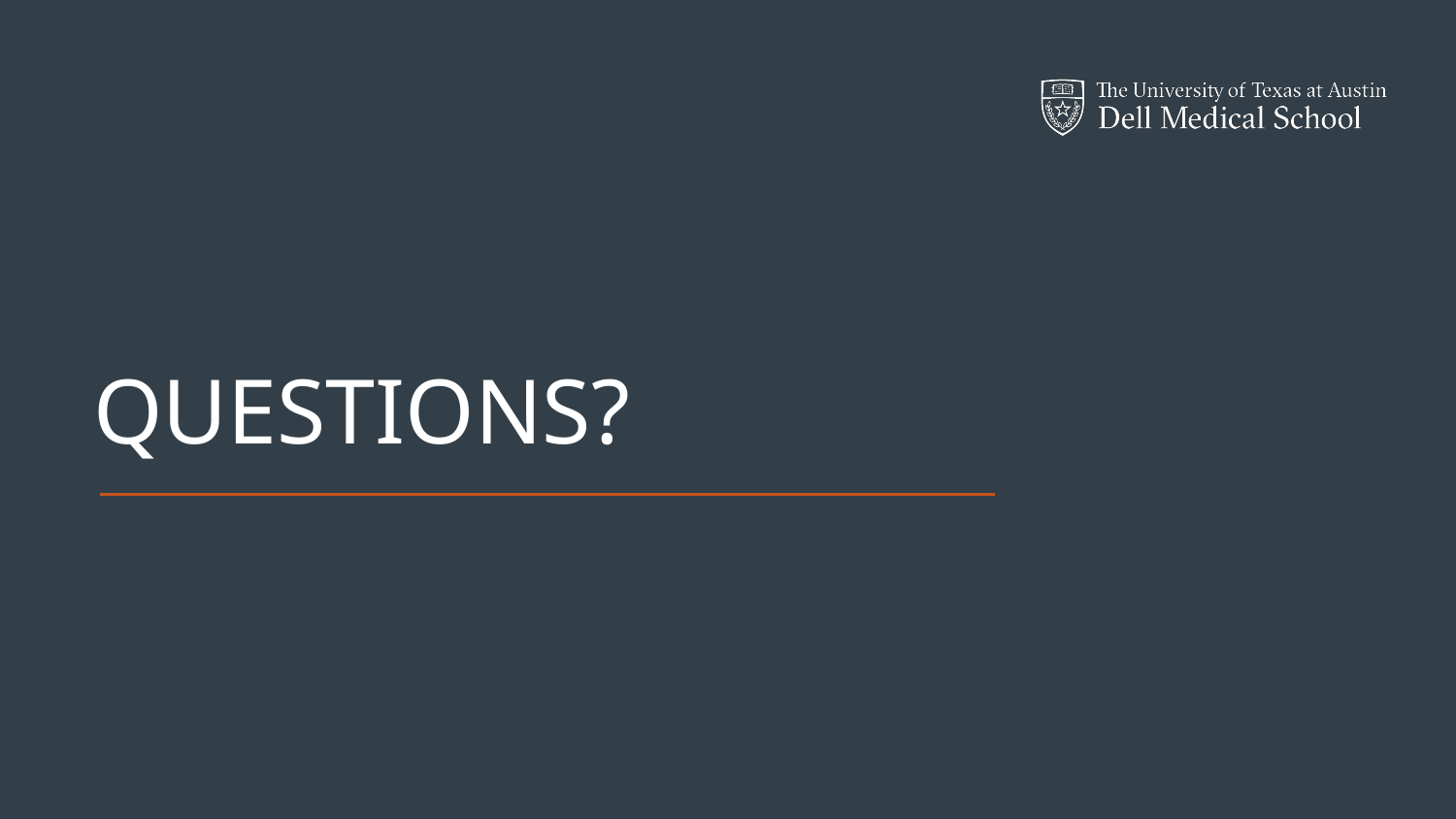

# QUESTIONS?
