# Supplementary material for: Implementing a Screening, Brief Intervention, and Referral to Treatment Curriculum for Medical Students on their Emergency Department Rotation
Source: MedEdPORTAL. 2026 Jan 13;22:11569. doi: 10.15766/mep_2374-8265.11569 (PMC12796009; doi:10.15766/mep_2374-8265.11569)
Supplement: Supplementary file 1 — Medical Student MI-SBIRT Curriculum.pptxAlcohol Use Disorder Identification Test.docxDrug Abuse Screening Test (DAST-10).docxSBIRT Algorithm.docxSP Case Descriptions.docxSP Case.docxStudent OSCE Instructions.docxSubstance Use Facts Sheet.docxSBIRT Brief Intervention Card.docxSample OSCE Schedule.xlsxPatient Follow-Up Guide.docxStudent SBIRT Patient Follow-Up Survey.docxMI-SBIRT Attitudes and Preparedness Survey.docxPre- and Postcurriculum Assessment.docxStudent-Administered SBIRT Form.docxPost-SBIRT Patient Feedback Form.docxOSCE Score Sheet.docxExceeds Criteria.docxStudent Workflow and Protocol.docx [file mep_2374-8265.11569-s001.zip › C. Drug Abuse Screening Test (DAST-10).docx]

**Appendix C: Drug Abuse Screening Test (DAST-10)**

To be reviewed during the didactics portion and utilized during student OSCE and real patient encounters

**
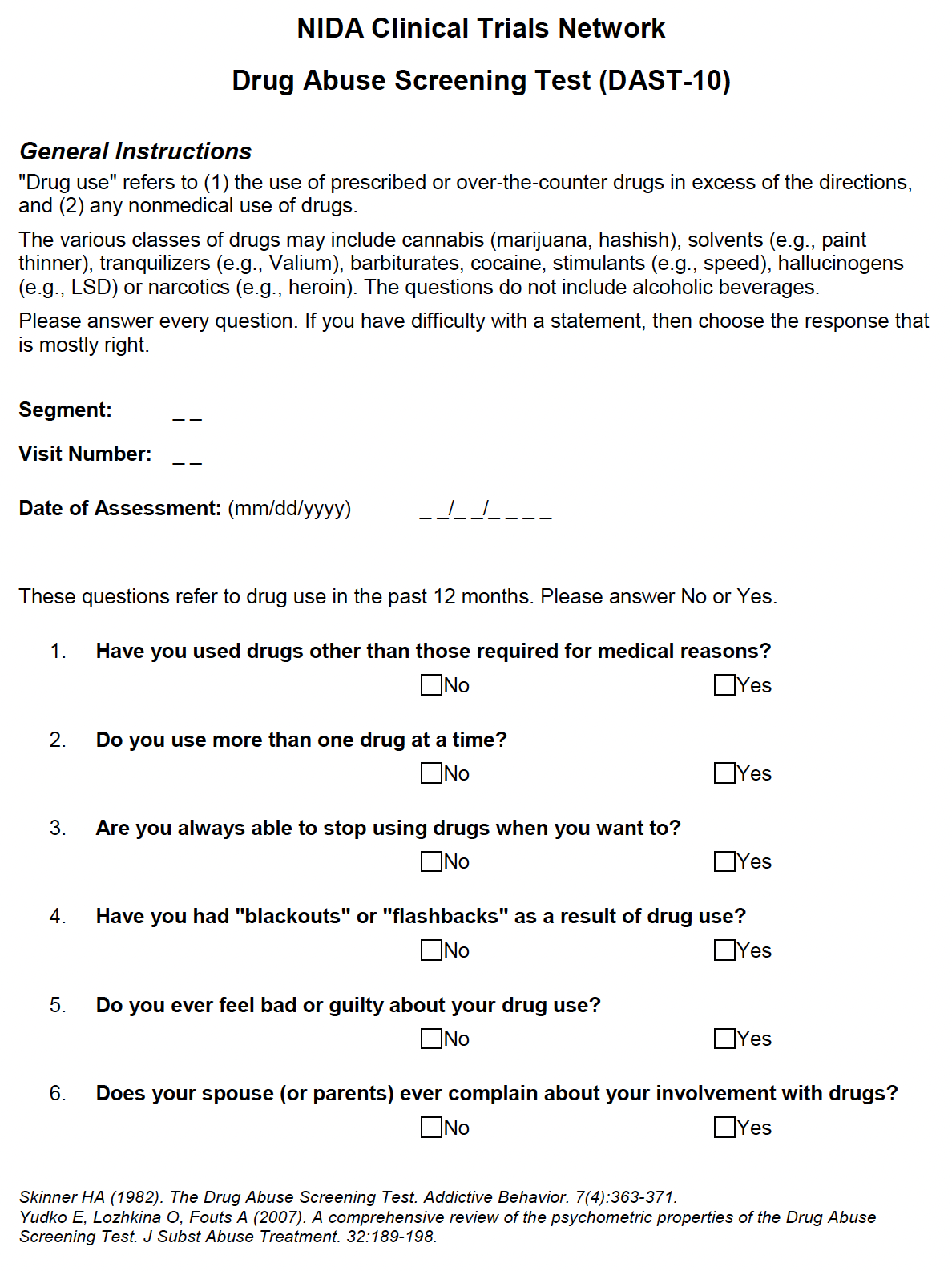
**

**
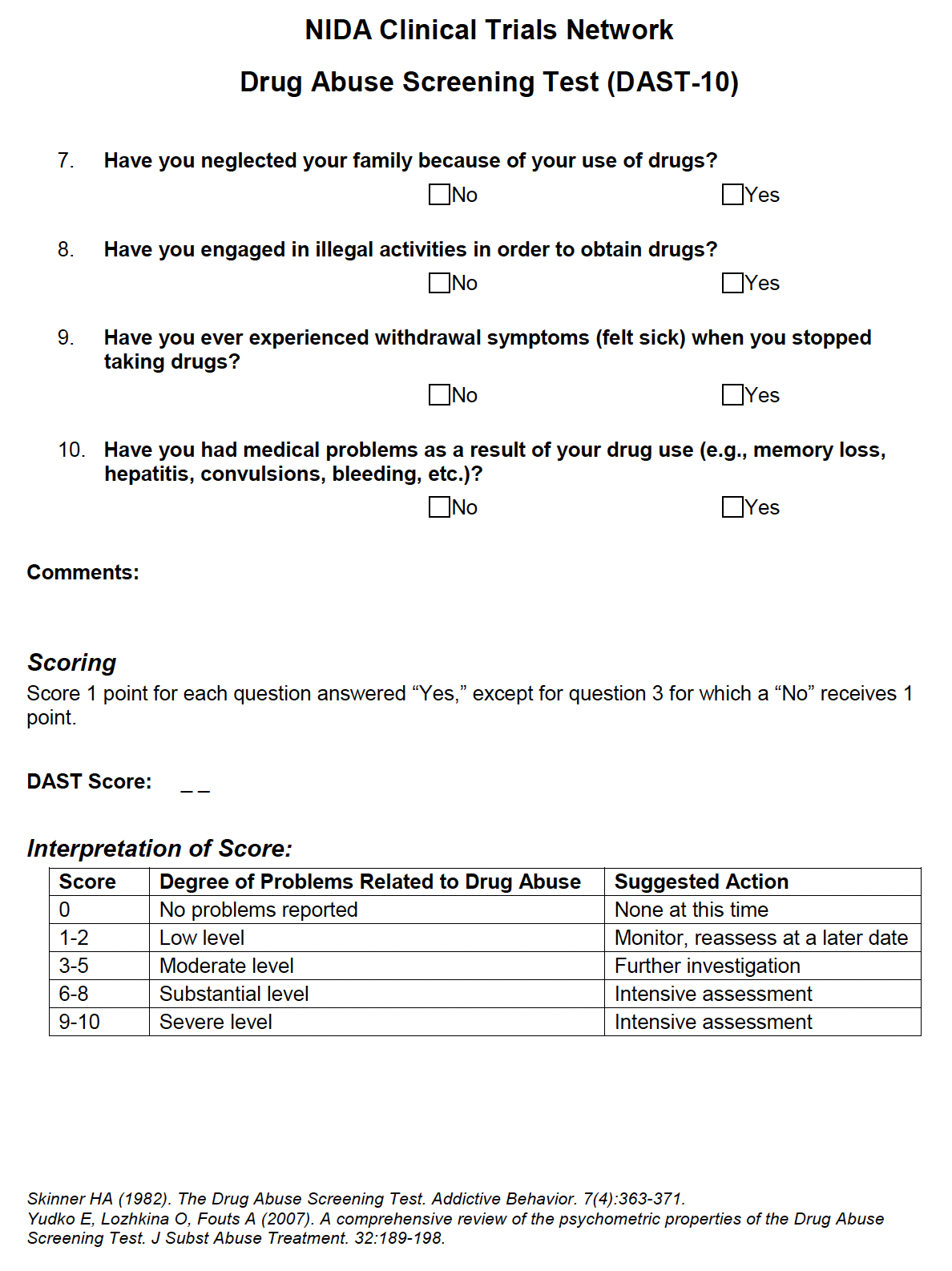
**

Image retrieved from <https://www.sdsuduip.com/forms/> on 9/1/2023. Permission to use granted with proper attribution to author B. F. Skinner. See copyright and reference below.

*© Copyright 1982 by the test author Dr. Harvey Skinner, York University, Toronto, Canada and by the Centre for Addiction and Mental Health (CAMH), Toronto, Canada. No unauthorized copying, distribution or amendment without the written permission of Dr. Harvey Skinner and the Centre for Addiction and Mental Health.*

Skinner HA (1982). The Drug Abuse Screening Test. Addictive Behavior. 7(4):363-371. Yudko E, Lozhkina O, Fouts A (2007). A comprehensive review of the psychometric properties of the Drug Abuse Screening Test. J Subst Abuse Treatment. 32:189-198.
