# Supplementary material for: Implementing a Screening, Brief Intervention, and Referral to Treatment Curriculum for Medical Students on their Emergency Department Rotation
Source: MedEdPORTAL. 2026 Jan 13;22:11569. doi: 10.15766/mep_2374-8265.11569 (PMC12796009; doi:10.15766/mep_2374-8265.11569)
Supplement: Supplementary file 1 — Medical Student MI-SBIRT Curriculum.pptxAlcohol Use Disorder Identification Test.docxDrug Abuse Screening Test (DAST-10).docxSBIRT Algorithm.docxSP Case Descriptions.docxSP Case.docxStudent OSCE Instructions.docxSubstance Use Facts Sheet.docxSBIRT Brief Intervention Card.docxSample OSCE Schedule.xlsxPatient Follow-Up Guide.docxStudent SBIRT Patient Follow-Up Survey.docxMI-SBIRT Attitudes and Preparedness Survey.docxPre- and Postcurriculum Assessment.docxStudent-Administered SBIRT Form.docxPost-SBIRT Patient Feedback Form.docxOSCE Score Sheet.docxExceeds Criteria.docxStudent Workflow and Protocol.docx [file mep_2374-8265.11569-s001.zip › D. SBIRT Algorithm.docx]

**Appendix D: SBIRT Algorithm**

To be reviewed during the didactics portion and utilized during student OSCE and real patient encounters

| 1. **BUILD RAPPORT** | **Tell me about a typical day in your life. Where does your current [X] use fit in?** |
| --- | --- |
| **2) PROS AND CONS** | **Help me understand, through your eyes, the good things about using [X].**  **What are some of the not-so-good things about using [X]?**  **So, on the one hand [PROS], and on the other hand [CONS].**  **(Reflect change talk)** |
| **3) INFORMATION AND FEEDBACK**  **Elicit**      **Provide**            **Elicit** | **I have some information on low-risk guidelines for drinking and drug use, would you mind if I shared them with you?**    **We know that drinking…**   - **3 or more (F)/ 4 or more (M) drinks on one occasion** - **Or more than 7 (F)/ 14(M) drinks in a week**   **…and/or use of illicit drugs such as ___**  **…can put you at risk for social or legal problems, as well as illness and injury. It can also cause health problems like [insert medical information]**  **What are your thoughts about that?** |
| **4) READINESS RULER** | **On a scale from 1-10, with 1 being not at all ready and 10 being completely ready, how ready are you to change your [X] use?**    **Why did you pick a __ and not a __?” (Always start with the higher number))**    **What would it take for you to move from a ___ to a ___ (start with lower number)**    **What concerns do you have about ___?**    **Let’s suppose you were a 10 sometime in the future. How do you think your life might be different?** |
| **5) ACTION PLAN**  **(if appropriate)**    **Identify strengths and supports**    **Offer appropriate resources** | **What are some steps/options that will work for you to stay healthy and safe?**    **What supports do you have for making this change?**    **Tell me about a challenge you overcame in the past. How can you use those supports/resources to help you now?**  **I have some additional resources that people sometimes find helpful; would you like to hear about them?** |
