# Supplementary material for: Implementing a Screening, Brief Intervention, and Referral to Treatment Curriculum for Medical Students on their Emergency Department Rotation
Source: MedEdPORTAL. 2026 Jan 13;22:11569. doi: 10.15766/mep_2374-8265.11569 (PMC12796009; doi:10.15766/mep_2374-8265.11569)
Supplement: Supplementary file 1 — Medical Student MI-SBIRT Curriculum.pptxAlcohol Use Disorder Identification Test.docxDrug Abuse Screening Test (DAST-10).docxSBIRT Algorithm.docxSP Case Descriptions.docxSP Case.docxStudent OSCE Instructions.docxSubstance Use Facts Sheet.docxSBIRT Brief Intervention Card.docxSample OSCE Schedule.xlsxPatient Follow-Up Guide.docxStudent SBIRT Patient Follow-Up Survey.docxMI-SBIRT Attitudes and Preparedness Survey.docxPre- and Postcurriculum Assessment.docxStudent-Administered SBIRT Form.docxPost-SBIRT Patient Feedback Form.docxOSCE Score Sheet.docxExceeds Criteria.docxStudent Workflow and Protocol.docx [file mep_2374-8265.11569-s001.zip › E. SP Case Descriptions.docx]

**Appendix E: SP Case Descriptions + AUDIT/DAST Scores**

To be used to instruct / train standardized patient actors on their roles. AUDIT/DAST scores to be reviewed by standardized patients to help guide their responses to students; scores available to students to guide their SBIRT during the OSCE

**Jacob Montgomery**

(25 year old male, homeless vet – opioid use)

You are a 25-year-old veteran who has been homeless for the past 6 weeks after losing your job and the apartment you shared with a friend. You stayed with a cousin for a while, but she has kids and wouldn’t tolerate your drug use so asked you to leave. You were able to couch surf with friends for a little while, but have been on the street for the

last 2-3 weeks. You ended up in the ED after passing out due to heat exhaustion.

You were prescribed opioids due to a service-related injury prior to being discharged from the Army. The crack down from the VA in prescribing opioids made them hard to come by as time went on, so you began using heroin, which is readily available on the streets, and have since progressed to a heroin/fentanyl habit.

On the street, drug use is everywhere and most of your friends use drugs and smoke cigarettes as well. The temptations are many. But you also do not want to end up as an anonymous overdose statistic and really want to find a way to turn your life around. You feel the need to get back to work is a first critical step and recognize that you could use help from a drug program. But you can’t see how you could go to treatment and hold a job at the same time. This is not where you thought you would be at this early point in your adult life. There is no arguing how lousy and stuck you feel, and if quitting were to help you feel better you might consider it.

**Drug Abuse Screening Test: Jacob Montgomery**


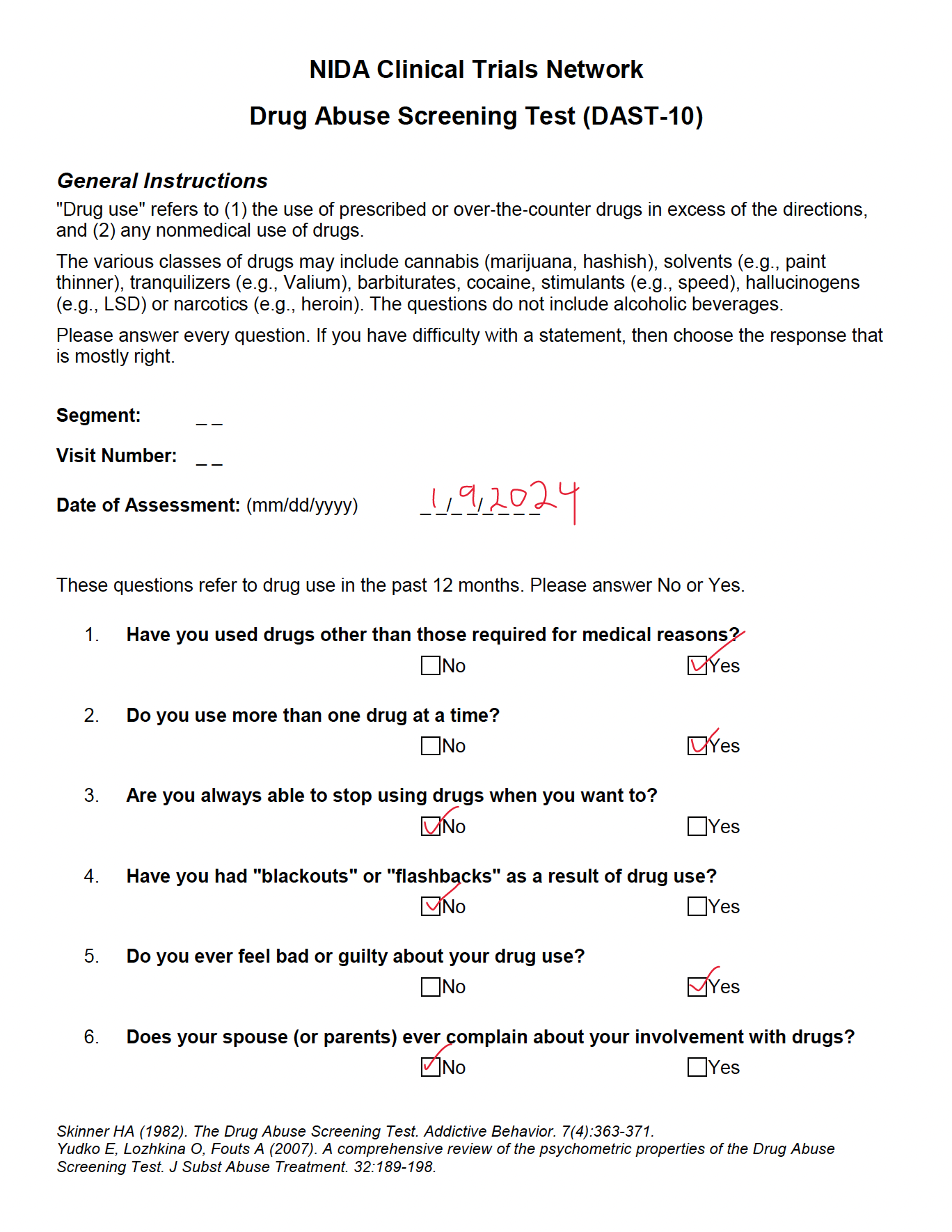


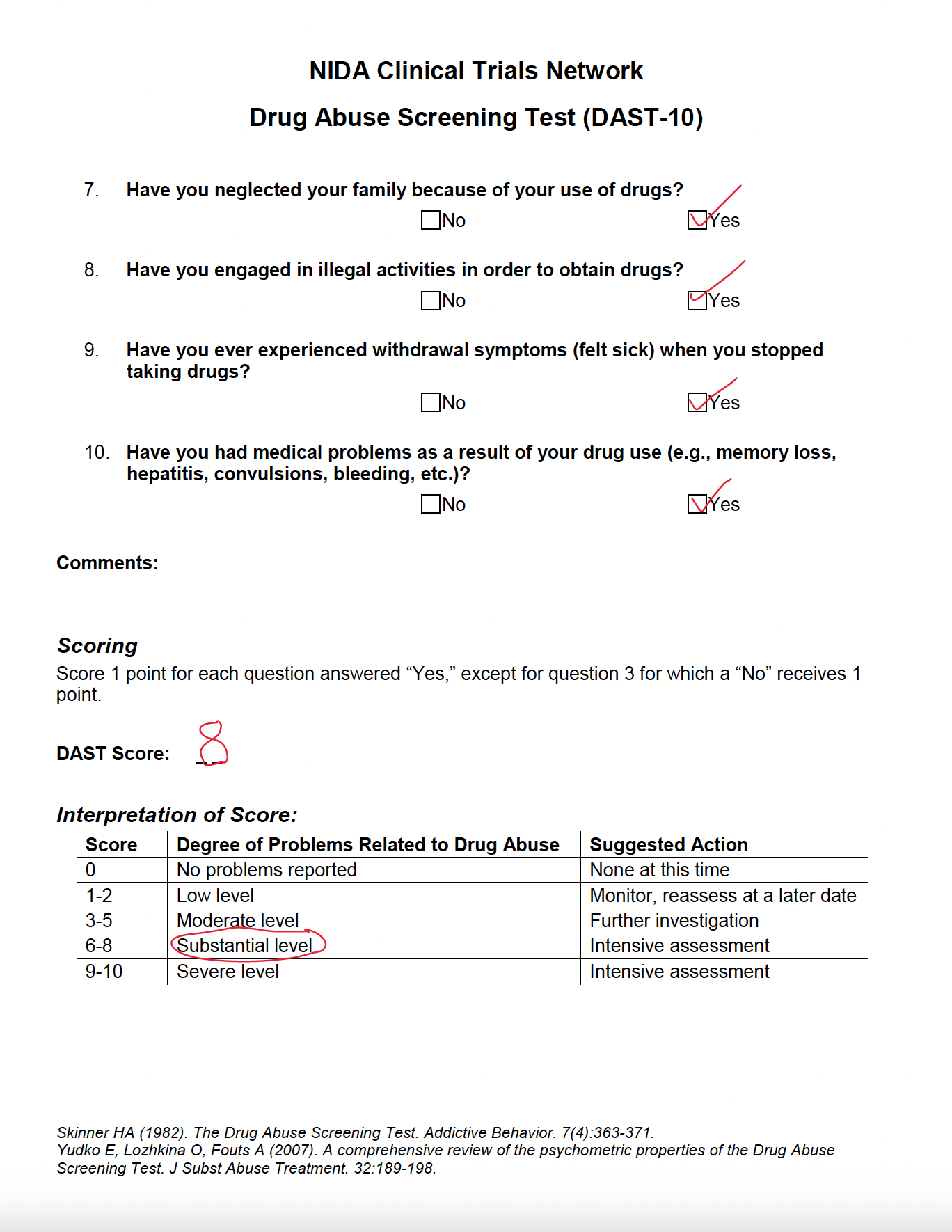


Image retrieved from <https://www.sdsuduip.com/forms/> on 9/1/2023. Permission to use granted with proper attribution to author B. F. Skinner. See copyright and reference below.

*© Copyright 1982 by the test author Dr. Harvey Skinner, York University, Toronto, Canada and by the Centre for Addiction and Mental Health (CAMH), Toronto, Canada. No unauthorized copying, distribution or amendment without the written permission of Dr. Harvey Skinner and the Centre for Addiction and Mental Health.*

Skinner HA (1982). The Drug Abuse Screening Test. Addictive Behavior. 7(4):363-371. Yudko E, Lozhkina O, Fouts A (2007). A comprehensive review of the psychometric properties of the Drug Abuse Screening Test. J Subst Abuse Treatment. 32:189-198.

**Jack Sanders**

29-year-old male, work from home – alcohol use

You present to the ED after falling while intoxicated. You tell the staff that you were drinking wine when this fall occurred, but that you don't drink much, you never miss work, and you are not really concerned about the amount you are drinking, You are concerned, however, that you may have broken your hip and won’t be able to work. Without income, you cannot pay your rent.

You live in a big city and work from home for a tech job since COVID. You have been drinking alcohol since your teens, but during COVID when your job moved from the office to "work from home," you began drinking about 1-2 bottles of wine throughout the day. It does not affect your job as you still complete all your duties, so you don't feel like you have a problem with alcohol.

You don't want to lose your job and want to heal from this injury so you can get back to your exercising that helped your mental health/stress from work.

**Alcohol Use Disorder Identification Test: Jack Sanders**


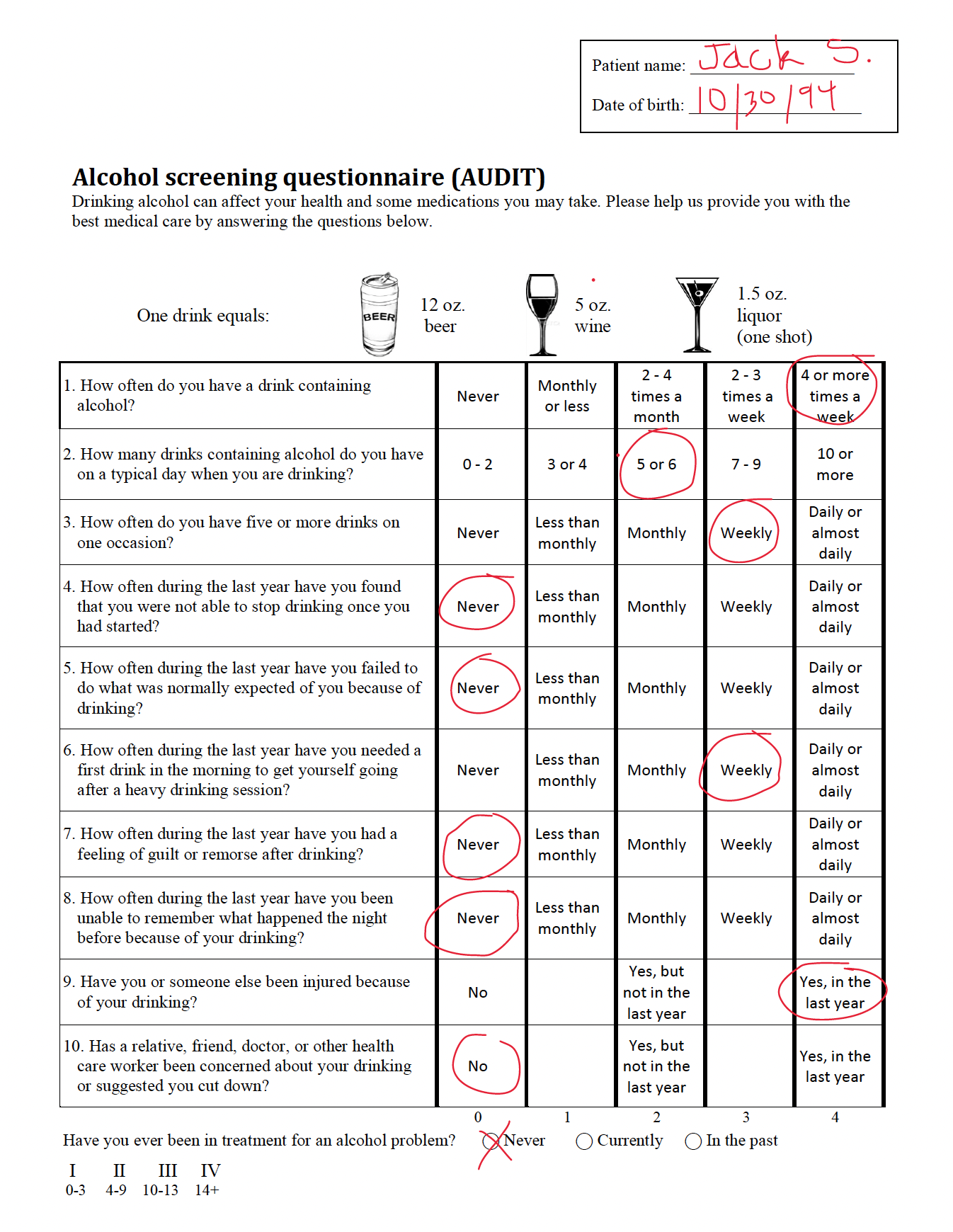


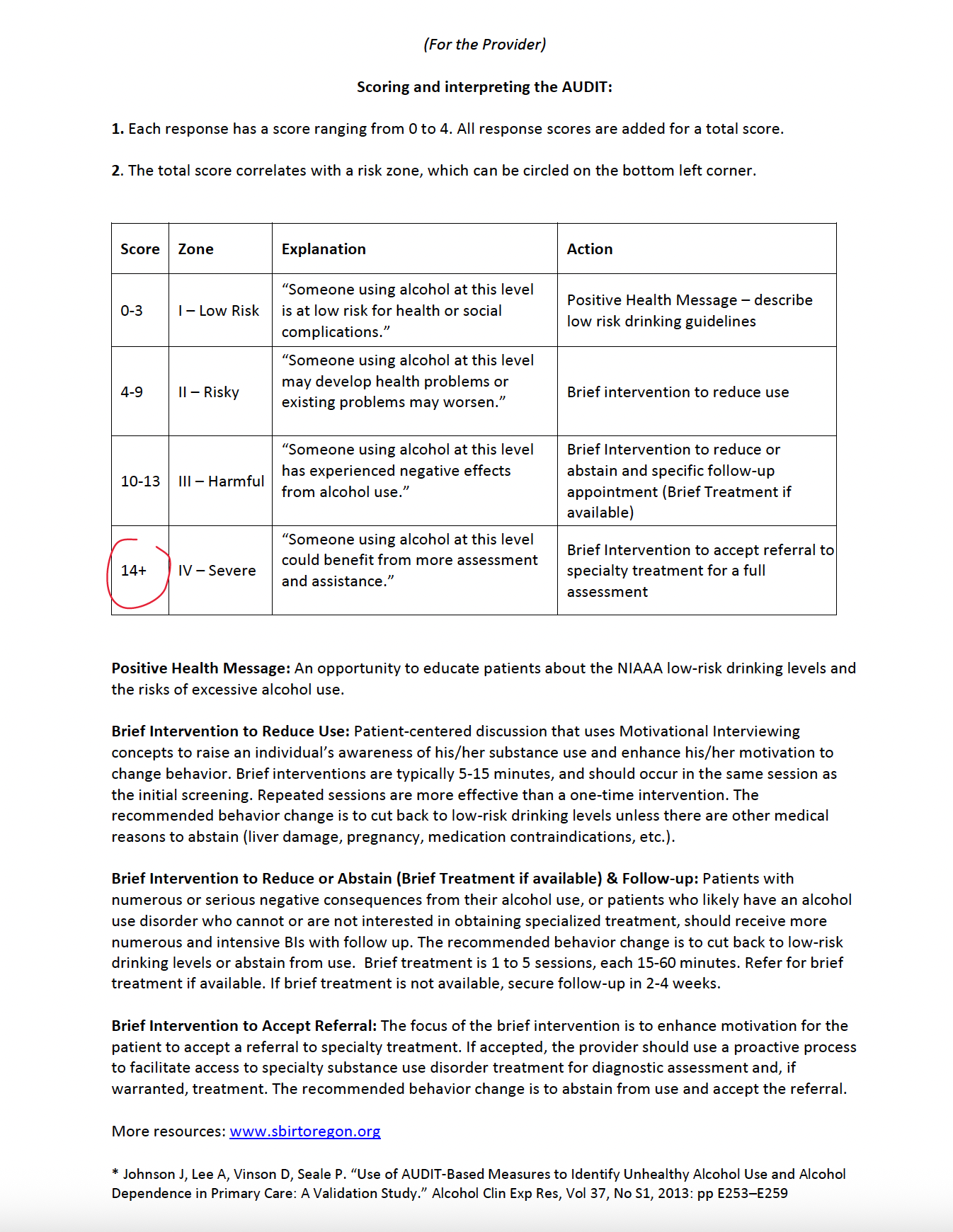


Image by The World Health Organization, retrieved from <https://auditscreen.org/> on 9/1/2023. Image is in the public domain.

**Olivia Harris**

(32 year old female, restaurant manager – alcohol and substance use)

You are a 32-year-old woman who presents to the ED with a severe kidney infection. You work long days as a restaurant manager at a local restaurant owned by family friends. Your main social supports are your coworkers and you often unwind after a long day of work drinking with them at the local bar next door and participating in some occasional drug use.

Over the last week, you have been experiencing more urinary frequency and pain with urination. These symptoms have rendered your long work hours unbearable. However, you feel a lot of pressure to not let down the owners of the restaurant – they have really been struggling to get the restaurant back on its feet since the pandemic and have made it clear how much they depend on you. Furthermore, because your working hours overlap with the normal hours of operation at the local doctor’s office, you haven’t been able to make an appointment for what you believe to be a urinary tract infection. You’ve been hoping that the symptoms will resolve on their own. Doctors, in general, have left a poor taste in your mouth after one experience in which you felt judged for your alcohol use. After confiding in a co-worker a few days ago about the pressure you are under, your symptoms, and your struggles to perform optimally, the coworker suggests more frequent happy hours with colleagues. This morning, you started feeling feverish with new onset back pain and made the decision to come to the ED where they diagnosed you with a kidney infection due to an untreated urinary tract infection.

**Alcohol Use Disorder Identification Test: Olivia Harris**

**
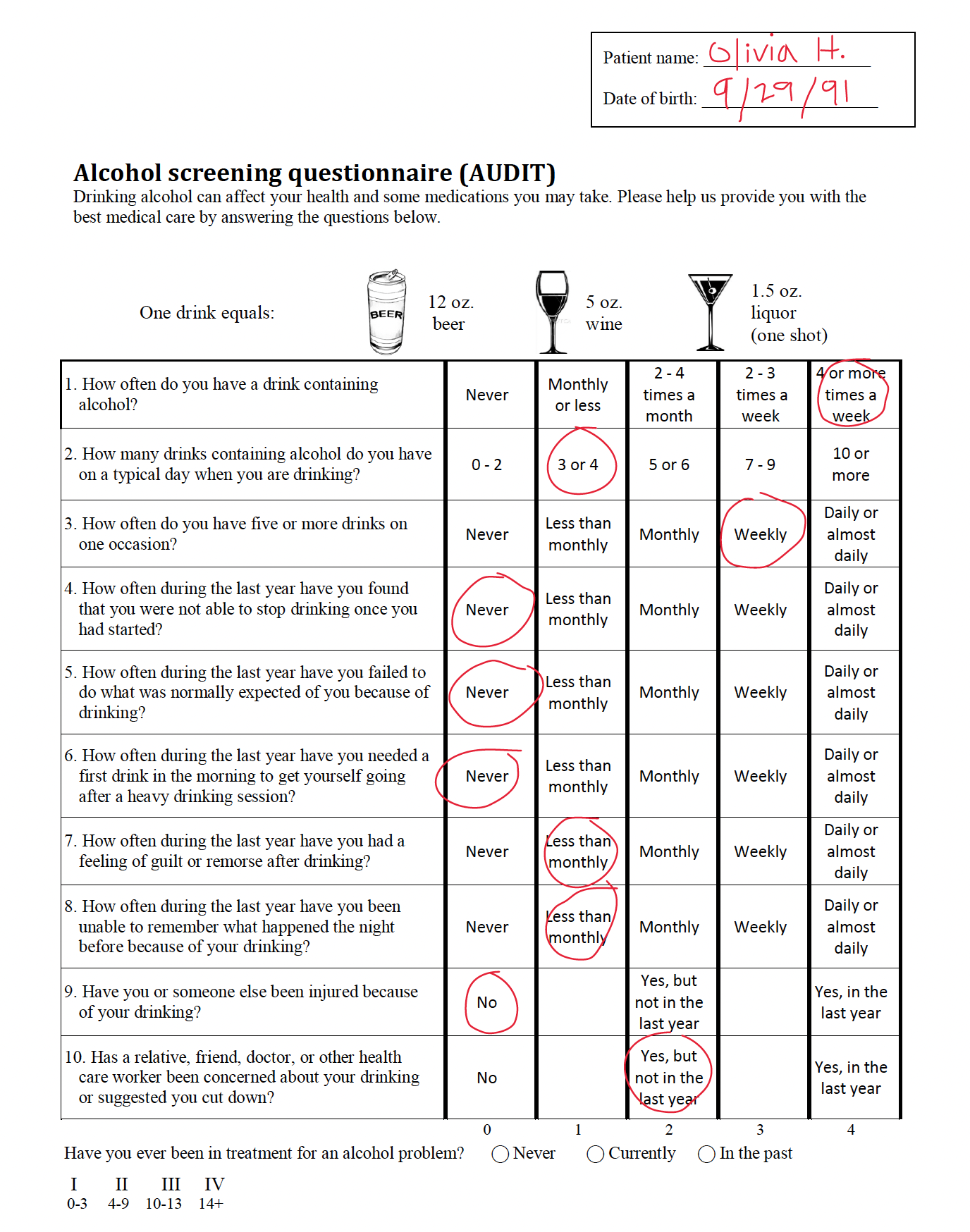
**

**
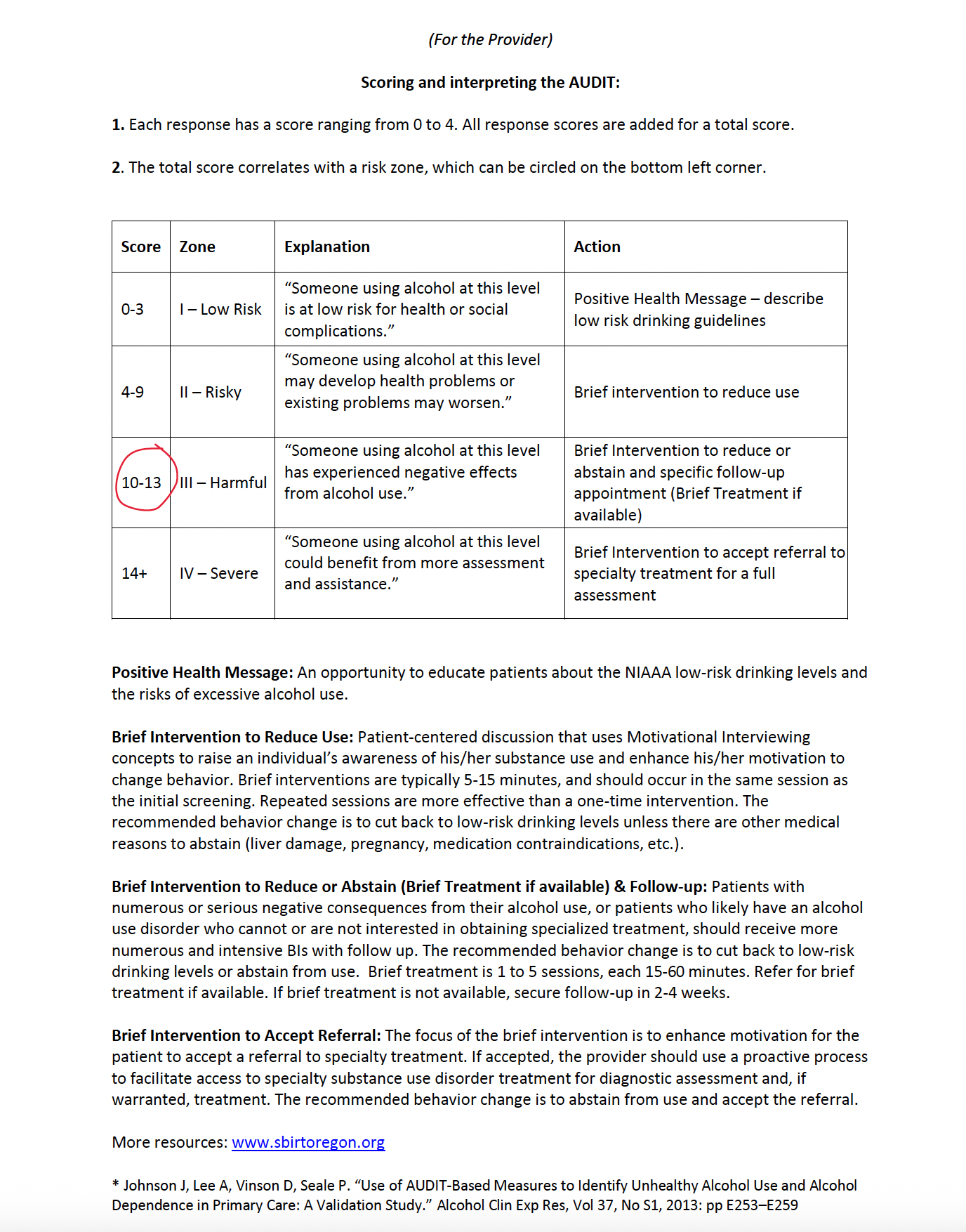
**

Image by The World Health Organization, retrieved from <https://auditscreen.org/> on 9/1/2023. Image is in the public domain.

**Drug Abuse Screening Test: Olivia Harris**

**
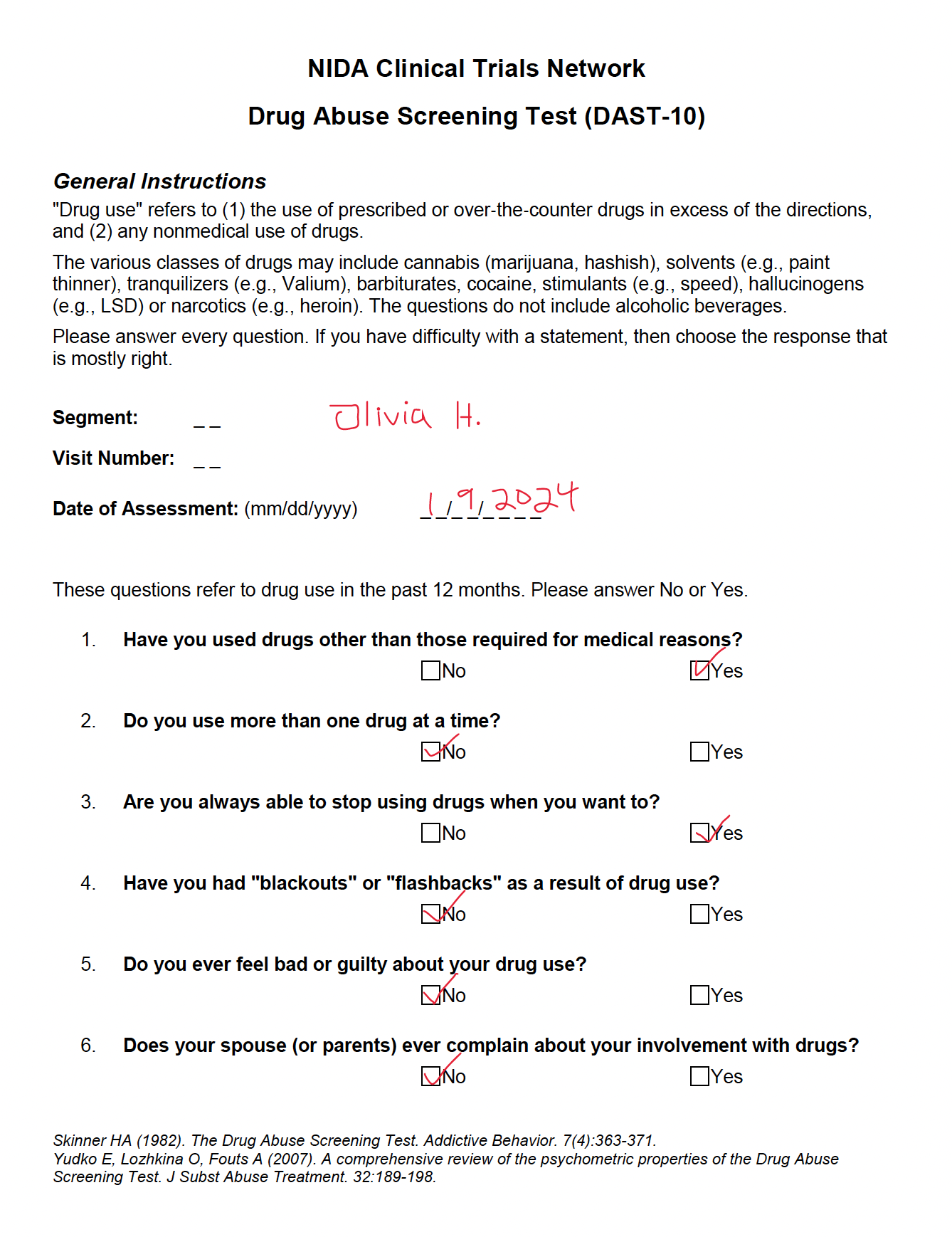
**

**
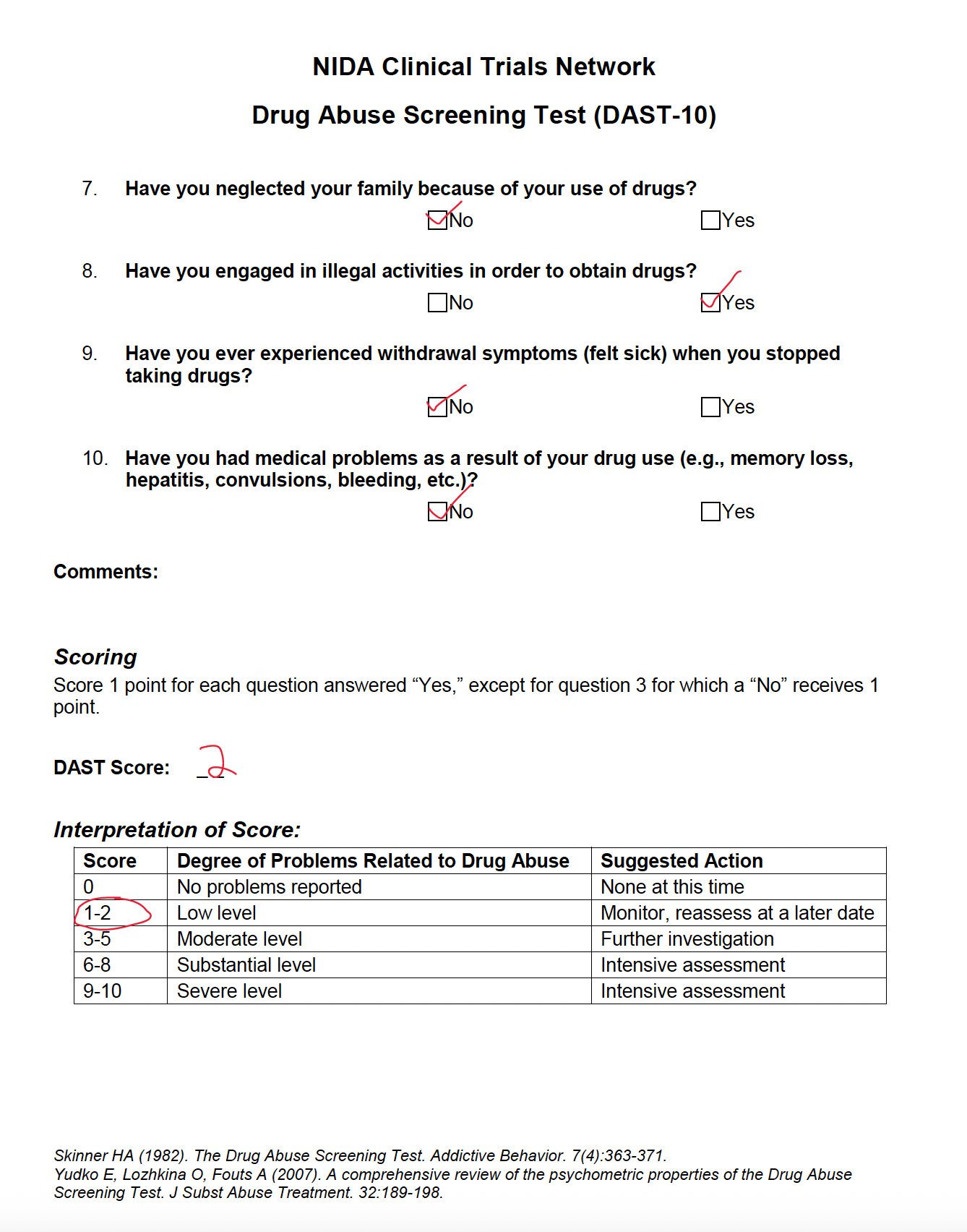
**

Image retrieved from <https://www.sdsuduip.com/forms/> on 9/1/2023. Permission to use granted with proper attribution to author B. F. Skinner. See copyright and reference below.

*© Copyright 1982 by the test author Dr. Harvey Skinner, York University, Toronto, Canada and by the Centre for Addiction and Mental Health (CAMH), Toronto, Canada. No unauthorized copying, distribution or amendment without the written permission of Dr. Harvey Skinner and the Centre for Addiction and Mental Health.*

Skinner HA (1982). The Drug Abuse Screening Test. Addictive Behavior. 7(4):363-371. Yudko E, Lozhkina O, Fouts A (2007). A comprehensive review of the psychometric properties of the Drug Abuse Screening Test. J Subst Abuse Treatment. 32:189-198.
