# Supplementary material for: Implementing a Screening, Brief Intervention, and Referral to Treatment Curriculum for Medical Students on their Emergency Department Rotation
Source: MedEdPORTAL. 2026 Jan 13;22:11569. doi: 10.15766/mep_2374-8265.11569 (PMC12796009; doi:10.15766/mep_2374-8265.11569)
Supplement: Supplementary file 1 — Medical Student MI-SBIRT Curriculum.pptxAlcohol Use Disorder Identification Test.docxDrug Abuse Screening Test (DAST-10).docxSBIRT Algorithm.docxSP Case Descriptions.docxSP Case.docxStudent OSCE Instructions.docxSubstance Use Facts Sheet.docxSBIRT Brief Intervention Card.docxSample OSCE Schedule.xlsxPatient Follow-Up Guide.docxStudent SBIRT Patient Follow-Up Survey.docxMI-SBIRT Attitudes and Preparedness Survey.docxPre- and Postcurriculum Assessment.docxStudent-Administered SBIRT Form.docxPost-SBIRT Patient Feedback Form.docxOSCE Score Sheet.docxExceeds Criteria.docxStudent Workflow and Protocol.docx [file mep_2374-8265.11569-s001.zip › F. SP Case.docx]

**Appendix F: MedEdPORTAL Standardized Patient Case Development Tool**

To be used to further introduce and train standardized patients in their roles ahead of the OSCE

Date: 9/1/2023

Primary Case Author: Samuel Burr, MD

Secondary Case Author: Dr. Mary Velasquez, PhD

Standardized Patient Educators: Dr. Mary Velasquez, PhD; Dr. Kirk Von Sternberg, PhD, Patrick Kennedy, MSSW

Name of Case: Jacob Montgomery; 25-Year-Old Male, Homeless Veteran, Opioid Use

Name of Educational and/or Assessment Activity: Objective Structured Clinical Exam

Patient Name: Jacob Montgomery

Chief Complaint: Syncope

Most Likely Diagnosis and Differential With Rationale From History and/or Physical Exam: Syncope

Challenge Question: He has indicated that he’d like to talk with someone about his substance use. Your attending has asked you to have this discussion with the patient.

Domains: Check all that apply

- Professionalism
- Communication and Interpersonal Skills


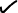


- Medical History
- Physical Exam
- Shared Decision-Making


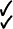


- Patient Education
- Clinical Reasoning
- Documentation
- Handoff
- Presentation
- Other: Motivational Interviewing


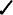


Type and Level of Learner: Second-year medical student

Case Objectives: Please list specific objectives for each of the domains you have checked above:

1. Review the patient’s Drug Abuse Screening Test (DAST) responses with the patient and discuss their score and associated risk category using the Screening, Brief Intervention, Referral to Treatment (SBIRT) Brief Intervention Card

2. Engage patient using motivational interviewing techniques to explore the patient’s ambivalence around substance use, elicit and provide information regarding their substance use, assess their readiness to change their behavior, and ask questions meant to increase their readiness

3. Based on the patient’s readiness to change, determine the appropriate next step(s) (eg, supporting autonomy/exploring negative consequences, offering social work consultation, developing action plan)

| SETTING: outpatient, in patient, ED, home, nursing home, rehab, group, etc. | Emergency Department |
| --- | --- |
| PATIENT PROFILE: Information about the “patient” that helps select an SP and helps the learner get an understanding of them as a person. SP will know more information about the patient than learner will ever ask but allows SP to portray a fully developed patient personality. If none of the items below are particulars for the case, please write “all may be used.” | |
| Age range | 25 years old |
| Religious/spiritual background | N/a |
| Sex (e.g., male, female, intersex, transwoman, transman) | Male |
| Sexual orientation (e.g., heterosexual, lesbian, gay, bisexual, pansexual, queer, asexual) | Heterosexual |
| Gender expression (e.g., man, woman, genderqueer) | Man |
| Race and ethnicity (e.g., to promote educational diversity, we use a diverse pool of SPs.) | N/a |
| Physical description (e.g., BMI, height range) | N/a |
| Physical limitations | N/a |
| Patient appearance (e.g., disheveled, hospital gown, business casual, casual) | Casual |
| Moulage + location (e.g., none, bruises, scars, body piercing, tattoos) | N/a |
| Affect (e.g., pleasant, cooperative) | Pleasant, cooperative; contemplative stage of change |
| Family group (e.g., who is family, who they live with) | Homeless after losing job and apartment shared with friend; also recently kicked out of cousin’s house due to use of drugs. |
| Education | N/a |
| Level of health literacy | N/a |
| Employment, if any - present and past, noting any current stresses | Recently discharged from Army with service-related injury for which opioids were prescribed |
| Home/homeless - type of dwelling, number of stories, owned or rented | Homeless |
| Financial situation - any current stresses | Critical, food and housing insecure |
| Insurance status (e.g., un/under/insured, public/private, HMO/PPO) | Uninsured |
| Habits (i.e., diet, exercise, caffeine, smoking, alcohol, drugs) | Use of prescription opioids which progressed to heroin/fentanyl |
| Activities (i.e., hobbies, sports, clubs, friends) | N/a |
| Typical day - what is the usual daily routine | On the street, drug use is everywhere and most of his friends use drugs and smoke cigarettes as well. The temptations are many. He does not want to end up as an anonymous overdose statistic and really wants to find a way to turn his life around. He is actively looking for work and recognizes that he could use help from a drug rehabilitation program. |

| CASE INFORMATION | |
| --- | --- |
| Chief Concern: What the patient will say when greeted by the student. The patient’s primary reason for seeking medical care often stated in their own words. | “I passed out.” |
| Additional Concerns: Other, if any, concerns the patient has today (i.e., symptoms, requests, expectations, etc.) that will become part of set agenda. | Heroin addiction, homelessness |
| THE PATIENT’S STORY: The SP will be asked to tell their symptom story and the personal and emotion impact for each of their concerns. You will want to write this in the patient’s voice. The symptom story should be able to answer this question: “Tell me more about [chief concern/additional concern], starting at the beginning and bringing me up to now.”  The personal context should be able to answer questions concerning the broader personal/psychosocial context of symptoms, especially the patient’s beliefs/attributions.  The emotional context should be able to ask how are you doing with this, how does this make you feel, how has this affected you emotionally? IMPACT: How has this affected your life? How has this been for your family? | Jacob is a 25-year-old veteran who has been homeless for the past 6 weeks after losing his job and the apartment he shared with a friend. He stayed with a cousin for a while, but she has kids and wouldn’t tolerate Jacob’s drug use so she asked him to leave. He was able to couch surf with friends for a little while, but has been on the street for the  last 2-3 weeks. He ended up in the ED after passing out due to heat exhaustion.  He was prescribed opioids due to a service-related injury prior to being discharged from the Army. The crack down from the VA in prescribing opioids made them hard to come by as time went on, so he began using heroin, which is readily available on the streets, and has since progressed to a heroin/fentanyl habit.  On the street, drug use is everywhere and most of his friends use drugs and smoke cigarettes as well. The temptations are many. But he also does not want to end up as an anonymous overdose statistic and really wants to find a way to turn his life around. He feels the need to get back to work is a first critical step and recognizes that he could use help from a drug program. But he can’t see how he could go to treatment and hold a job at the same time. This is not where he thought he would be at this early point in his adult life. There is no arguing how lousy and stuck he feels, and if quitting were to help him feel better he might consider it. |
| HISTORY OF PRESENT ILLNESS: Although some of the HPI will be given in the patient’s symptom story, the learners will expand the story during the direct question section. Below, describe the detailed history, usually about the chief concern, which the student must develop in order to make a useful assessment of the problem: | |
| Onset (when; gradual or sudden) | Day of admission |
| Setting (what was going on or where was patient when symptoms first noticed?) | Waiting for bus to arrive |
| Duration (how long) | One minute |
| Time relationships (frequency, constant or intermittent) | First time |
| Location | N/a |
| Radiation | N/a |
| Quality | N/a |
| Amount | N/a |
| Aggravated by what | Heat, dehydration |
| Relieved by what | Hydration, being out of the sun |
| Associated with what | Heat |
| Attitude (what does the patient think is the problem, and how do they feel about it) | Believes it to be due to heat exhaustion / not sure |
| Overall course | Improving |
| REVIEW OF SYSTEMS: Significant positives and negatives | |
|  |  |
|  |  |
|  |  |
|  |  |
|  |  |
| Past medical history | N/a |
| Medication allergies (name and reaction) | N/a |
| Environmental allergies (name and reaction) | N/a |
| Illnesses | N/a |
| Vaccinations | N/a |
| Surgeries | N/a |
| Accidents/injuries/trauma | N/a |
| Hospitalization | N/a |
|  | |
| Inclusive sexual and reproductive history | |
| Sexual practices  Sexual partners  Protection: Use of safer sex practices  Use of birth control if appropriate  Risk of intimate partner violence | N/a |
| OB/GYN history | Age of onset of menses  Age of menopause  Number of pregnancies  Number of live births  Number of miscarriages  Number of abortions |
| Medications | Prescription/dose/reason  Over the counter/dose/reason  Herbs/supplements/dose/reason  Other: |
| Immunizations | - Tetanus - Flu - Hepatitis - Pneumovax - HPV - Other |
| Tobacco products:   - Cigarettes  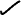  - Cigar - Pipe - Chew - E-cigarettes | - Never - Past - year started/year quit - Current   - Quantity   - # of years |
| Alcohol   - Beer - Wine - Liquor - Other | - Never - Past - year started/year quit - Current   - Quantity   - # of years |
| Drugs   - Weed - Cocaine  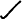  - Heroin - Meth - IV - Inhalants - Other | - Never - Past - year started/year quit - Current   - Quantity: daily   - # of years: 1 year |
| Diet (describe) | N/a |
| Exercise (describe) | N/a |
| List any other important social history or information important to this case | N/a |
| Family history | N/a |
| Mother, father, siblings, grandparents, and other significant findings | N/a |
|  |  |
| Physical Exam - List exam maneuvers expected for this case and any abnormal findings that SP will simulate. (tenderness, hyper-hypo reflex, rebound, weakness, etc.) N/a | |
| PHYSICAL EXAM FINDINGS | N/a |
| 1. Written in layperson’s terms | N/a |
| 1. General appearance - affect, appearance, position of patient at opening (i.e., sitting, lying down, holding abdomen, etc.) | N/a |
| 1. Vital signs | N/a |
| 1. Specific findings and affect | N/a |
| 1. Response to certain physical movements | N/a |
|  |  |
| DIAGNOSIS AND DIFFERENTIAL |  |
| Diagnosis with support from positive and negative history and PE findings | N/a |
| Differential with support from positive and negative history and PE findings | N/a |
|  |  |
| MANAGEMENT OR DIAGNOSTIC PLAN | N/a |
|  |  |
| PROFESSIONALISM ISSUES OR CHALLENGES | N/a |

Date: 9/1/2023

Primary Case Author: Samuel Burr, MD

Secondary Case Author: Dr. Mary Velasquez, PhD

Standardized Patient Educators: Dr. Mary Velasquez, PhD; Dr. Kirk Von Sternberg, PhD, Patrick Kennedy, MSSW

Name of Case: Jack Sanders, 29-Year-Old Male, Alcohol Misuse

Name of Educational and/or Assessment Activity: Objective Structured Clinical Exam

Patient Name: Jack Sanders

Chief Complaint: Fall from standing

Most Likely Diagnosis and Differential With Rationale From History and/or Physical Exam: Mechanical fall

Challenge Question: Your attending has asked that you have a conversation with Mr. Sanders about his alcohol consumption while the hip x-ray is being read by the radiologist.

Domains: Check all that apply

- Professionalism
- Communication and Interpersonal Skills


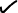


- Medical History
- Physical Exam
- Shared Decision-Making


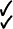


- Patient Education
- Clinical Reasoning
- Documentation
- Handoff
- Presentation
- Other: Motivational Interviewing


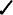


Type and Level of Learner: Second-year medical student

Case Objectives: Please list specific objectives for each of the domains you have checked above:

1. Review the patient’s Drug Abuse Screening Test (DAST) responses with the patient and discuss their score and associated risk category using the Screening, Brief Intervention, Referral to Treatment (SBIRT) Brief Intervention Card

2. Engage patient using motivational interviewing techniques to explore the patient’s ambivalence around substance use, elicit and provide information regarding their substance use, assess their readiness to change their behavior, and ask questions meant to increase their readiness

3. Based on the patient’s readiness to change, determine the appropriate next step(s) (eg, supporting autonomy/exploring negative consequences, offering social work consultation, developing action plan)

| SETTING: outpatient, in patient, ED, home, nursing home, rehab, group, etc. | Emergency Department |
| --- | --- |
| PATIENT PROFILE: Information about the “patient” that helps select an SP and helps the learner get an understanding of them as a person. SP will know more information about the patient than learner will ever ask but allows SP to portray a fully developed patient personality. If none of the items below are particulars for the case, please write “all may be used.” | |
| Age range | 29 years old |
| Religious/spiritual background | N/a |
| Sex (e.g., male, female, intersex, transwoman, transman) | Male |
| Sexual orientation (e.g., heterosexual, lesbian, gay, bisexual, pansexual, queer, asexual) | Heterosexual |
| Gender expression (e.g., man, woman, genderqueer) | Man |
| Race and ethnicity (e.g., to promote educational diversity, we use a diverse pool of SPs.) | N/a |
| Physical description (e.g., BMI, height range) | N/a |
| Physical limitations | N/a |
| Patient appearance (e.g., disheveled, hospital gown, business casual, casual) | Casual |
| Moulage + location (e.g., none, bruises, scars, body piercing, tattoos) | N/a |
| Affect (e.g., pleasant, cooperative) | Anxious, cooperative; pre-contemplative stage of change |
| Family group (e.g., who is family, who they live with) | Lives alone |
| Education | College |
| Level of health literacy | N/a |
| Employment, if any - present and past, noting any current stresses | Works in tech; works from home due to transition out of office during Covid-19 pandemic |
| Home/homeless - type of dwelling, number of stories, owned or rented | Apartment |
| Financial situation - any current stresses | Stable, though anxious about losing job due to productivity loss associated with injury |
| Insurance status (e.g., un/under/insured, public/private, HMO/PPO) | Insured |
| Habits (i.e., diet, exercise, caffeine, smoking, alcohol, drugs) | Alcohol use, 1-2 bottles of wine daily |
| Activities (i.e., hobbies, sports, clubs, friends) | N/a |
| Typical day - what is the usual daily routine | Lives in a big city, works from home in tech. Has been drinking alcohol since he was a teenager. It doesn’t affect his job – he’s able to complete his requisite tasks. |

| CASE INFORMATION | |
| --- | --- |
| Chief Concern: What the patient will say when greeted by the student. The patient’s primary reason for seeking medical care often stated in their own words. | “I fell at home.” |
| Additional Concerns: Other, if any, concerns the patient has today (i.e., symptoms, requests, expectations, etc.) that will become part of set agenda. | Anxious about losing job due to injury |
| THE PATIENT’S STORY: The SP will be asked to tell their symptom story and the personal and emotion impact for each of their concerns. You will want to write this in the patient’s voice. The symptom story should be able to answer this question: “Tell me more about [chief concern/additional concern], starting at the beginning and bringing me up to now.”  The personal context should be able to answer questions concerning the broader personal/psychosocial context of symptoms, especially the patient’s beliefs/attributions.  The emotional context should be able to ask how are you doing with this, how does this make you feel, how has this affected you emotionally? IMPACT: How has this affected your life? How has this been for your family? | Jack presents to the ED after falling while intoxicated. He tells the staff that he was drinking wine when this fall occurred, but that he doesn’t drink much, he never misses work, and he is not really concerned about the amount he is drinking, He is concerned, however, that he may have broken his hip and won’t be able to work. Without income, he can’t pay his rent.  Jack lives in a big city and works a tech job from home. He’s been working from home since COVID. He’s been drinking alcohol since his teens, but after transitioning to working from home during COVID, he began drinking about 1-2 bottles of wine throughout the day. He claims it doesn’t affect his job as he still completes all his duties, so he doesn’t feel like he has a problem with alcohol.  He doesn’t want to lose his job and wants to heal from this injury so he can get back to his exercising which helped his mental health/stress from work. |
| HISTORY OF PRESENT ILLNESS: Although some of the HPI will be given in the patient’s symptom story, the learners will expand the story during the direct question section. Below, describe the detailed history, usually about the chief concern, which the student must develop in order to make a useful assessment of the problem: | |
| Onset (when; gradual or sudden) | Day of admission |
| Setting (what was going on or where was patient when symptoms first noticed?) | At home, after significant alcohol consumption |
| Duration (how long) | A few hours prior to arrival |
| Time relationships (frequency, constant or intermittent) | First time |
| Location | Right hip |
| Radiation | N/a |
| Quality | Throbbing |
| Amount | 8/10 |
| Aggravated by what | Weight bearing |
| Relieved by what | Staying off of hip/sitting/lying down |
| Associated with what | N/a |
| Attitude (what does the patient think is the problem, and how do they feel about it) | Thinks his hip is broken |
| Overall course | Improving |
| REVIEW OF SYSTEMS: Significant positives and negatives | |
|  |  |
|  |  |
|  |  |
|  |  |
|  |  |
| Past medical history | N/a |
| Medication allergies (name and reaction) | N/a |
| Environmental allergies (name and reaction) | N/a |
| Illnesses | N/a |
| Vaccinations | N/a |
| Surgeries | N/a |
| Accidents/injuries/trauma | N/a |
| Hospitalization | N/a |
|  | |
| Inclusive sexual and reproductive history | |
| Sexual practices  Sexual partners  Protection: Use of safer sex practices  Use of birth control if appropriate  Risk of intimate partner violence | N/a |
| OB/GYN history | Age of onset of menses  Age of menopause  Number of pregnancies  Number of live births  Number of miscarriages  Number of abortions |
| Medications | Prescription/dose/reason  Over the counter/dose/reason  Herbs/supplements/dose/reason  Other: |
| Immunizations | - Tetanus - Flu - Hepatitis - Pneumovax - HPV - Other |
| Tobacco products:   - Cigarettes - Cigar - Pipe - Chew - E-cigarettes | - Never - Past - year started/year quit - Current   - Quantity   - # of years |
| Alcohol   - Beer - Wine  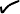  - Liquor - Other | - Never - Past - year started/year quit - Current   - Quantity: 2 bottles/day   - 0.5 years |
| Drugs   - Weed - Cocaine - Heroin - Meth - IV - Inhalants - Other | - Never - Past - year started/year quit - Current   - Quantity:   - # of years: |
| Diet (describe) | N/a |
| Exercise (describe) | N/a |
| List any other important social history or information important to this case | N/a |
| Family history | N/a |
| Mother, father, siblings, grandparents, and other significant findings | N/a |
|  |  |
| Physical Exam - List exam maneuvers expected for this case and any abnormal findings that SP will simulate. (tenderness, hyper-hypo reflex, rebound, weakness, etc.) N/a | |
| PHYSICAL EXAM FINDINGS | N/a |
| 1. Written in layperson’s terms | N/a |
| 1. General appearance - affect, appearance, position of patient at opening (i.e., sitting, lying down, holding abdomen, etc.) | N/a |
| 1. Vital signs | N/a |
| 1. Specific findings and affect | N/a |
| 1. Response to certain physical movements | N/a |
|  |  |
| DIAGNOSIS AND DIFFERENTIAL |  |
| Diagnosis with support from positive and negative history and PE findings | N/a |
| Differential with support from positive and negative history and PE findings | N/a |
|  |  |
| MANAGEMENT OR DIAGNOSTIC PLAN | N/a |
|  |  |
| PROFESSIONALISM ISSUES OR CHALLENGES | N/a |

Date: 9/1/2023

Primary Case Author: Samuel Burr, MD

Secondary Case Author: Dr. Mary Velasquez, PhD

Standardized Patient Educators: Dr. Mary Velasquez, PhD; Dr. Kirk Von Sternberg, PhD, Patrick Kennedy, MSSW

Name of Case: Olivia Harris, 32-Year-Old Female, Mixed Risky Alcohol and Drug Use

Name of Educational and/or Assessment Activity: Objective Structured Clinical Exam

Patient Name: Olivia Harris

Chief Complaint: Dysuria, back pain

Most Likely Diagnosis and Differential With Rationale From History and/or Physical Exam: Uncomplicated pyelonephritis

Challenge Question: During your social history, she mentioned she uses alcohol frequently with co-workers after work, though was hesitant to do so. Your attending has asked you to assess this further.

Domains: Check all that apply

- Professionalism
- Communication and Interpersonal Skills


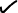


- Medical History
- Physical Exam
- Shared Decision-Making


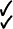


- Patient Education
- Clinical Reasoning
- Documentation
- Handoff
- Presentation
- Other: Motivational Interviewing


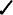


Type and Level of Learner: Second-year medical student

Case Objectives: Please list specific objectives for each of the domains you have checked above:

1. Review the patient’s Drug Abuse Screening Test (DAST) responses with the patient and discuss their score and associated risk category using the Screening, Brief Intervention, Referral to Treatment (SBIRT) Brief Intervention Card

2. Engage patient using motivational interviewing techniques to explore the patient’s ambivalence around substance use, elicit and provide information regarding their substance use, assess their readiness to change their behavior, and ask questions meant to increase their readiness

3. Based on the patient’s readiness to change, determine the appropriate next step(s) (eg, supporting autonomy/exploring negative consequences, offering social work consultation, developing action plan)

| SETTING: outpatient, in patient, ED, home, nursing home, rehab, group, etc. | Emergency Department |
| --- | --- |
| PATIENT PROFILE: Information about the “patient” that helps select an SP and helps the learner get an understanding of them as a person. SP will know more information about the patient than learner will ever ask but allows SP to portray a fully developed patient personality. If none of the items below are particulars for the case, please write “all may be used.” | |
| Age range | 32 years old |
| Religious/spiritual background | N/a |
| Sex (e.g., male, female, intersex, transwoman, transman) | Female |
| Sexual orientation (e.g., heterosexual, lesbian, gay, bisexual, pansexual, queer, asexual) | Heterosexual |
| Gender expression (e.g., man, woman, genderqueer) | Woman |
| Race and ethnicity (e.g., to promote educational diversity, we use a diverse pool of SPs.) | N/a |
| Physical description (e.g., BMI, height range) | N/a |
| Physical limitations | N/a |
| Patient appearance (e.g., disheveled, hospital gown, business casual, casual) | Casual |
| Moulage + location (e.g., none, bruises, scars, body piercing, tattoos) | N/a |
| Affect (e.g., pleasant, cooperative) | Anxious, cooperative; contemplative stage of change |
| Family group (e.g., who is family, who they live with) | Lives alone |
| Education | N/a |
| Level of health literacy | N/a |
| Employment, if any - present and past, noting any current stresses | Works as a restaurant manager, overworked with lots of pressure not to let the owners of the restaurant down resulting in few days off |
| Home/homeless - type of dwelling, number of stories, owned or rented | Apartment |
| Financial situation - any current stresses | Stable |
| Insurance status (e.g., un/under/insured, public/private, HMO/PPO) | Insured, though normal work hours overlap with hours of operation at local doctor’s office. |
| Habits (i.e., diet, exercise, caffeine, smoking, alcohol, drugs) | Alcohol use, 3-4 drinks daily at local bar + occasional marijuana / cocaine use |
| Activities (i.e., hobbies, sports, clubs, friends) | N/a |
| Typical day - what is the usual daily routine | Works at a restaurant. Upon restaurant close, patient will drink and with coworkers at bar that is next door to the restaurant. Some nights, she’ll engage in drug use. |

| CASE INFORMATION | |
| --- | --- |
| Chief Concern: What the patient will say when greeted by the student. The patient’s primary reason for seeking medical care often stated in their own words. | “It’s been burning when I urinate for the past week and now I have a fever and back pain.” |
| Additional Concerns: Other, if any, concerns the patient has today (i.e., symptoms, requests, expectations, etc.) that will become part of set agenda. | Feels overworked, is struggling to find ways to decompress after work besides drinking with coworkers. |
| THE PATIENT’S STORY: The SP will be asked to tell their symptom story and the personal and emotion impact for each of their concerns. You will want to write this in the patient’s voice. The symptom story should be able to answer this question: “Tell me more about [chief concern/additional concern], starting at the beginning and bringing me up to now.”  The personal context should be able to answer questions concerning the broader personal/psychosocial context of symptoms, especially the patient’s beliefs/attributions.  The emotional context should be able to ask how are you doing with this, how does this make you feel, how has this affected you emotionally? IMPACT: How has this affected your life? How has this been for your family? | Olivia is a 32-year-old woman who presents to the ED with a severe kidney infection. She works long days as a restaurant manager at a local restaurant owned by family friends. Her main social supports are her coworkers and she often unwinds after a long day of work drinking with them at the local bar next door. On some of these outings, she’ll engage in drug use with her co-workers.  Over the last week, she has been experiencing more urinary frequency and pain with urination. These symptoms have rendered her long work hours unbearable. However, she feels a lot of pressure to not let down the owners of the restaurant – they have really been struggling to get the restaurant back on its feet since the pandemic and have made it clear how much they depend on her. Furthermore, because her working hours overlap with the normal hours of operation at the local doctor’s office, she hasn’t been able to make an appointment for what she believes to be a urinary tract infection. She’d been hoping that the symptoms would resolve on their own. Doctors, in general, have left a poor taste in her mouth after one experience in which she felt judged for her alcohol use. After confiding in a co-worker a few days ago about the pressure she’s under, her symptoms, and her struggles to perform optimally, her coworker suggested more frequent happy hours with colleagues. This morning, she started feeling feverish with new onset back pain and made the decision to come to the ED where she was diagnosed with a kidney infection due to an untreated urinary tract infection. |
| HISTORY OF PRESENT ILLNESS: Although some of the HPI will be given in the patient’s symptom story, the learners will expand the story during the direct question section. Below, describe the detailed history, usually about the chief concern, which the student must develop in order to make a useful assessment of the problem: | |
| Onset (when; gradual or sudden) | Gradual |
| Setting (what was going on or where was patient when symptoms first noticed?) | At work |
| Duration (how long) | One week |
| Time relationships (frequency, constant or intermittent) | Constant |
| Location | Bladder/right flank |
| Radiation | N/a |
| Quality | Burning/throbbing |
| Amount | 4-8/10 |
| Aggravated by what | Urinating |
| Relieved by what | Fluid restriction |
| Associated with what | Fevers |
| Attitude (what does the patient think is the problem, and how do they feel about it) | Thinks it’s a worsening urinary tract infection |
| Overall course | Improving |
| REVIEW OF SYSTEMS: Significant positives and negatives | |
|  |  |
|  |  |
|  |  |
|  |  |
|  |  |
| Past medical history | N/a |
| Medication allergies (name and reaction) | N/a |
| Environmental allergies (name and reaction) | N/a |
| Illnesses | N/a |
| Vaccinations | N/a |
| Surgeries | N/a |
| Accidents/injuries/trauma | N/a |
| Hospitalization | N/a |
|  | |
| Inclusive sexual and reproductive history | |
| Sexual practices  Sexual partners  Protection: Use of safer sex practices  Use of birth control if appropriate  Risk of intimate partner violence | N/a |
| OB/GYN history | Age of onset of menses  Age of menopause  Number of pregnancies  Number of live births  Number of miscarriages  Number of abortions |
| Medications | Prescription/dose/reason  Over the counter/dose/reason  Herbs/supplements/dose/reason  Other: |
| Immunizations | - Tetanus - Flu - Hepatitis - Pneumovax - HPV - Other |
| Tobacco products:   - Cigarettes - Cigar - Pipe - Chew - E-cigarettes | - Never - Past - year started/year quit - Current   - Quantity   - # of years |
| Alcohol   - Beer  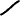  - Wine - Liquor  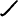  - Other | - Never - Past - year started/year quit - Current   - Quantity: Equivalent of 3-4 standard drinks/day   - 5 years |
| Drugs   - Weed  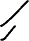  - Cocaine - Heroin - Meth - IV - Inhalants - Other | - Never - Past - year started/year quit - Current   - Quantity: Once a week   - # of years: 5 years |
| Diet (describe) | N/a |
| Exercise (describe) | N/a |
| List any other important social history or information important to this case | N/a |
| Family history | N/a |
| Mother, father, siblings, grandparents, and other significant findings | N/a |
|  |  |
| Physical Exam - List exam maneuvers expected for this case and any abnormal findings that SP will simulate. (tenderness, hyper-hypo reflex, rebound, weakness, etc.) N/a | |
| PHYSICAL EXAM FINDINGS | N/a |
| 1. Written in layperson’s terms | N/a |
| 1. General appearance - affect, appearance, position of patient at opening (i.e., sitting, lying down, holding abdomen, etc.) | N/a |
| 1. Vital signs | N/a |
| 1. Specific findings and affect | N/a |
| 1. Response to certain physical movements | N/a |
|  |  |
| DIAGNOSIS AND DIFFERENTIAL |  |
| Diagnosis with support from positive and negative history and PE findings | N/a |
| Differential with support from positive and negative history and PE findings | N/a |
|  |  |
| MANAGEMENT OR DIAGNOSTIC PLAN | N/a |
|  |  |
| PROFESSIONALISM ISSUES OR CHALLENGES | N/a |
