# Supplementary material for: Implementing a Screening, Brief Intervention, and Referral to Treatment Curriculum for Medical Students on their Emergency Department Rotation
Source: MedEdPORTAL. 2026 Jan 13;22:11569. doi: 10.15766/mep_2374-8265.11569 (PMC12796009; doi:10.15766/mep_2374-8265.11569)
Supplement: Supplementary file 1 — Medical Student MI-SBIRT Curriculum.pptxAlcohol Use Disorder Identification Test.docxDrug Abuse Screening Test (DAST-10).docxSBIRT Algorithm.docxSP Case Descriptions.docxSP Case.docxStudent OSCE Instructions.docxSubstance Use Facts Sheet.docxSBIRT Brief Intervention Card.docxSample OSCE Schedule.xlsxPatient Follow-Up Guide.docxStudent SBIRT Patient Follow-Up Survey.docxMI-SBIRT Attitudes and Preparedness Survey.docxPre- and Postcurriculum Assessment.docxStudent-Administered SBIRT Form.docxPost-SBIRT Patient Feedback Form.docxOSCE Score Sheet.docxExceeds Criteria.docxStudent Workflow and Protocol.docx [file mep_2374-8265.11569-s001.zip › G. Student OSCE Instructions.docx]

**Appendix G: Student OSCE Instructions**

To be used to introduce students to the standardized patient they’ll encounter immediately prior to the OSCE encounter itself. Students have one minute to review this information and their patient’s AUDIT/DAST scores prior to their encounter start time.

**Jacob Montgomery**

(25-year-old male, homeless vet – opioid use)

**DAST score:** 8 (substantial use)

**Student Instructions:** Mr. Montgomery is a 25-year-old veteran experiencing homelessness who was brought to the ED by EMS after a brief syncopal episode while waiting at a bus stop. His exam was remarkable for hyperthermia, mild hypotension, tachycardia, and signs of dehydration – concerning for heat exhaustion. He has responded well to cold IV crystalloids and rapid cooling measures. He has indicated that he’d like to talk with someone about his substance use. Your attending has asked you to have this discussion with the patient. Please complete the following steps with the patient within the time allotted:

1. Review the patient’s DAST responses with the patient and discuss their score and associated risk category using the SBIRT Brief Intervention Card
2. Engage patient using motivational interviewing techniques to explore the patient’s ambivalence around substance use, elicit and provide information regarding their substance use, assess their readiness to change their behavior, and ask questions meant to increase their readiness
3. Based on the patient’s readiness to change, determine the appropriate next step(s) (eg, supporting autonomy/exploring negative consequences, offer social work consultation, develop action plan)

**Jack Sanders**

(29-year-old male, alcohol misuse)

**AUDIT score:** 16 (high risk for developing alcohol use disorder)

**Student Instructions:** Mr. Sanders is a 29-year-old male who’s presented to the ED after a fall from standing on his right hip while intoxicated. Since his arrival, he’s undergone radiologic work-up and has received ibuprofen for pain control which he states is controlling the pain as long as he doesn’t move around too much. Your attending has asked that you have a conversation with Mr. Sanders about his alcohol consumption while the hip XR is read by the radiologist. Please complete the following steps with the patient within the time allotted:

1. Review the patient’s AUDIT responses with the patient and discuss their score and associated risk category using the SBIRT Brief Intervention Card
2. Engage patient using motivational techniques to explore the patient’s ambivalence around substance use, elicit and provide information regarding their substance use, assess their readiness to change their behavior, and ask questions meant to increase their readiness
3. Based on the patient’s readiness to change, determine the appropriate next step(s) (eg, supporting autonomy/exploring negative consequences, offer social work consultation, develop action plan)

**Olivia Harris**

(32-year-old female, mixed risky alcohol and drug use)

**AUDIT score:** 12 (harmful use)

**DAST score:** 2 (risky use)

**Student instructions:** Olivia Harris is a 32-year-old woman who presents to the ED complaining of fever and back pain following a week of dysuria and urinary frequency, suspicious for pyelonephritis. She is pending a urinalysis, blood and urine cultures, and has since been started on empiric oral ciprofloxacin. During your social history, she mentioned she uses alcohol frequently with co-workers after work, though was hesitant to do so. Your attending has asked you to assess this further. Please complete the following steps with the patient within the time allotted:

1. Review the patient’s AUDIT/DAST responses with the patient and discuss their score and associated risk category using the SBIRT Brief Intervention Card
2. Engage patient using motivational techniques to explore the patient’s ambivalence around substance use, elicit and provide information regarding their substance use, assess their readiness to change their behavior, and ask questions meant to increase their readiness
3. Based on the patient’s readiness to change, determine the appropriate next step(s) (eg, supporting autonomy/exploring negative consequences, offer social work consultation, develop action plan)
