# Supplementary material for: Implementing a Screening, Brief Intervention, and Referral to Treatment Curriculum for Medical Students on their Emergency Department Rotation
Source: MedEdPORTAL. 2026 Jan 13;22:11569. doi: 10.15766/mep_2374-8265.11569 (PMC12796009; doi:10.15766/mep_2374-8265.11569)
Supplement: Supplementary file 1 — Medical Student MI-SBIRT Curriculum.pptxAlcohol Use Disorder Identification Test.docxDrug Abuse Screening Test (DAST-10).docxSBIRT Algorithm.docxSP Case Descriptions.docxSP Case.docxStudent OSCE Instructions.docxSubstance Use Facts Sheet.docxSBIRT Brief Intervention Card.docxSample OSCE Schedule.xlsxPatient Follow-Up Guide.docxStudent SBIRT Patient Follow-Up Survey.docxMI-SBIRT Attitudes and Preparedness Survey.docxPre- and Postcurriculum Assessment.docxStudent-Administered SBIRT Form.docxPost-SBIRT Patient Feedback Form.docxOSCE Score Sheet.docxExceeds Criteria.docxStudent Workflow and Protocol.docx [file mep_2374-8265.11569-s001.zip › H. Substance Use Facts Sheet.docx]

**Appendix H: Substance Use Facts Sheet**

To be available for student use during their OSCE and real patient encounters during the “elicit-provide-elicit” component of their brief intervention, as directed in the SBIRT Algorithm

Substance Use Facts Sheet

## For use during “Elicit-Provide-Elicit”

1. **Alcohol**^1^
   1. Medical consequences:
      1. (immediate) Raises blood pressure & cholesterol, can cause arrythmias
      2. (months to years) Can cause GI problems such as ulcers, reflux, pancreatitis
      3. (years) Can lead to long-term consequences such as liver disease
   2. Psychological/social consequences:
      1. Can lead to dependence
      2. Can worsen depression and anxiety
      3. Can lead to life-threatening withdrawals
      4. Can harm relationships
   3. Benefits of cutting back/quitting:
      1. Improved immunity
      2. Lowers cancer risk
      3. Lowers risk of heart disease
      4. Lowers stress level, improves self-confidence
      5. Better sleep
      6. Cognitive improvements
2. **Marijuana**^2^
   1. Medical consequences
      1. Can cause palpitations
      2. Impaired physical performance (reduced coordination, slower reactions)
   2. Psychological consequences
      1. Can lead to dependence
      2. Linked to worsening depression, anxiety, suicide planning, and psychotic episodes (rare)
   3. Benefits of cutting back/quitting
      1. Increased energy/motivation
      2. Improved focus
      3. Better memory
      4. Positive impact on mood, decreased anxiety
3. **Cocaine/methamphetamine**^3,4^
   1. Medical consequences
      1. (Immediate) Raises blood pressure & heart rate
      2. (Immediate) Increased risk of MI, seizures, strokes
      3. (Immediate) Risk of infectious disease contraction with IVDU
      4. (Chronic) Bowel ischemia, parkinsonian symptoms, inflamed cardiac myocardium
   2. Psychological consequences
      1. Dependency
      2. Irritability, panic attacks
      3. Paranoia, full-blown psychosis
      4. Significant harm to relationships
   3. Benefits of cutting back/quitting
      1. Neuronal recovery (regaining cognitive function)
      2. Reduced risk of cardiovascular complications, psychiatric complications
4. **Opiates**^5^
   1. Medical consequences
      1. Respiratory depression (deadly)
      2. Constipation, vomiting
      3. Flu-like sx with withdrawal
      4. Risk of infectious disease contraction with IVDU
      5. Seizures, muscle spasms
   2. Psychological consequences
      1. Dependency
      2. Mood swings, irritability
      3. Delusions, paranoia
      4. Significant harm to relationships
   3. Benefits of cutting back/quitting
      1. Reduced risk of overdose death
      2. Reduced risk of infectious disease contraction
      3. Reduced risk of seizures
      4. Improved mood, decreased anxiety/depression
5. **Benzodiazepines**^6^
   1. Medical consequences
      1. (Chronic) Cognitive impairment
      2. (Chronic) Increased risk of Alzheimer’s/dementia
      3. (Use-dependent) Life-threatening withdrawal (seizures)
   2. Psychological consequences
      1. Dependency
      2. Overdose risk (respiratory depression)
      3. Impaired sleep (rebound insomnia)
      4. Worsened anxiety/depression
   3. Benefits of cutting back/quitting
      1. Reduced risk of long-term cognitive impairment/ADRD
      2. Reduced risk of overdose/withdrawal related death
      3. Improved sleep
6. **K2 (Spice)**^7^
   1. Medical consequences
      1. (Short-term) Hypertension, increased heart rate, myocardial ischemia
      2. (Long-term) Seizures, renal impairment
   2. Psychological consequences
      1. Dependency
      2. Psychotic symptoms
      3. Depression, suicidal ideation
   3. Benefits of cutting back
      1. Inverse of the above

References

1. The Physical and Mental Health Benefits of Quitting Alcohol. Verywell Mind. Accessed November 24, 2023. https://www.verywellmind.com/what-are-the-benefits-of-alcohol-recovery-67761

2. Know the Risks of Marijuana. March 25, 2019. Accessed November 24, 2023. https://www.samhsa.gov/marijuana

3. Abuse NI on D. What are the long-term effects of cocaine use? | National Institute on Drug Abuse (NIDA). --. Accessed November 24, 2023. https://nida.nih.gov/publications/research-reports/cocaine/what-are-long-term-effects-cocaine-use

4. What Happens to the Brain During Recovery from Cocaine Use Disorder? Recovery Research Institute. December 5, 2017. Accessed November 24, 2023. https://www.recoveryanswers.org/research-post/brain-recovery-cocaine-use-disorder/

5. Opiate Abuse & Addiction Effects, Signs & Symptoms. Mount Regis Center. Accessed November 24, 2023. https://www.mtregis.com/opiates/effects-signs-symptoms/

6. Johnson B, Streltzer J. Risks Associated with Long-Term Benzodiazepine Use. *Am Fam Physician*. 2013;88(4):224-225.

7. K2 Spice Effects | Short-Term, Long-Term, & Side Effects. DrugAbuse.com. Accessed November 24, 2023. https://drugabuse.com/drugs/spice/effects-use/
