# Supplementary material for: Implementing a Screening, Brief Intervention, and Referral to Treatment Curriculum for Medical Students on their Emergency Department Rotation
Source: MedEdPORTAL. 2026 Jan 13;22:11569. doi: 10.15766/mep_2374-8265.11569 (PMC12796009; doi:10.15766/mep_2374-8265.11569)
Supplement: Supplementary file 1 — Medical Student MI-SBIRT Curriculum.pptxAlcohol Use Disorder Identification Test.docxDrug Abuse Screening Test (DAST-10).docxSBIRT Algorithm.docxSP Case Descriptions.docxSP Case.docxStudent OSCE Instructions.docxSubstance Use Facts Sheet.docxSBIRT Brief Intervention Card.docxSample OSCE Schedule.xlsxPatient Follow-Up Guide.docxStudent SBIRT Patient Follow-Up Survey.docxMI-SBIRT Attitudes and Preparedness Survey.docxPre- and Postcurriculum Assessment.docxStudent-Administered SBIRT Form.docxPost-SBIRT Patient Feedback Form.docxOSCE Score Sheet.docxExceeds Criteria.docxStudent Workflow and Protocol.docx [file mep_2374-8265.11569-s001.zip › K. Patient Follow-Up Guide.docx]

**Appendix K: Medical Student Phone Follow-up Guide/Script**

To be used in conjunction with the follow-up survey by project team to guide follow-up calls with patients to whom SBIRT was administered

Medical Student Phone Follow-up Guide/Script

# Please adhere to the framework below when following up with your patient.

## 2 & 4 Week Follow Up

1. Introduce self
2. Confirm who you’re speaking with
3. Explain reason for call: check-in
   1. Example: “Hi [patient name], we last spoke [two or four] weeks ago while you were in the emergency room. We talked about [SBIRT administration].
4. Ask how they’ve been doing, call back things they’d mentioned during the brief intervention. Has anything changed in their life? Have they implemented any changes? Note their responses in the Student SBIRT Patient Follow Up Survey.
5. Ask if they were able to access the resource they were referred to, if applicable. If they haven’t been able to follow-up with the resource, why not? Record their response and note it in the Student SBIRT Patient Follow Up Survey.
   1. Example: “we’re very interested in learning more about patient’s experiences following discharge in following up with the resources they were referred to. You mentioned you haven’t had a chance to follow up with [resource], would you be open to sharing some of the barriers you encountered, if any? Barriers could include something as simple as ‘it’s not a priority for me at this time.’”
6. If they have been able to follow-up with [resource], was it helpful? Why or why not? Begin by clarifying what their goal/hope was regarding the outcome of following up with this resource. Record their response and note it in the Student SBIRT Patient Follow Up Survey.
   1. Example: “I’m hearing you were able to follow up with [resource]. We’re very interested in learning whether these resources are helpful for the patients we refer them to, to help inform our referral process. Did you find that [resource] was helpful in taking positive steps toward accomplishing [their goal]?”
7. Note questions and concerns regarding accessing the resource to which they were referred, answer them if you have the capacity to. If unable to answer/satisfy their questions/concerns/requests, record the patient’s questions/concerns/requests in your notes, and send an encrypted email to [curriculum administrator, email URL] identifying your patient as simply, “my patient” (in lieu of any other identifier or PHI) detailing their questions/concerns for the curriculum team to review and address with the patient.
8. In the event that the patient has questions or concerns about their medical care or care coordination, utilize the below script, record the patient’s questions or requests in your notes, and send an encrypted email to [curriculum administrator, email URL] identifying your patient as simply, “my patient” (in lieu of any other identifier or PHI) detailing their question/concerns for the curriculum team to review and address with the patient:
   1. Example: “Thank you for asking this question, I’m not able to answer it and/or provide the appropriate medical guidance, but I’m happy to send a note to your clinical care team to review and check back in with you on this. What I’m entering is [xyz question/need/request], does that sound correct?”
   2. Please note whether the patient requested medical advice in the Student SBIRT Patient Follow Up Survey. If they did, please also note what advice they were seeking in the Student SBIRT Patient Follow Up Survey.
9. Summarize/clarify what was discussed during the call, thank the patient for their time, and end the call
