# Supplementary material for: Implementing a Screening, Brief Intervention, and Referral to Treatment Curriculum for Medical Students on their Emergency Department Rotation
Source: MedEdPORTAL. 2026 Jan 13;22:11569. doi: 10.15766/mep_2374-8265.11569 (PMC12796009; doi:10.15766/mep_2374-8265.11569)
Supplement: Supplementary file 1 — Medical Student MI-SBIRT Curriculum.pptxAlcohol Use Disorder Identification Test.docxDrug Abuse Screening Test (DAST-10).docxSBIRT Algorithm.docxSP Case Descriptions.docxSP Case.docxStudent OSCE Instructions.docxSubstance Use Facts Sheet.docxSBIRT Brief Intervention Card.docxSample OSCE Schedule.xlsxPatient Follow-Up Guide.docxStudent SBIRT Patient Follow-Up Survey.docxMI-SBIRT Attitudes and Preparedness Survey.docxPre- and Postcurriculum Assessment.docxStudent-Administered SBIRT Form.docxPost-SBIRT Patient Feedback Form.docxOSCE Score Sheet.docxExceeds Criteria.docxStudent Workflow and Protocol.docx [file mep_2374-8265.11569-s001.zip › L. Student SBIRT Patient Follow Up Survey.docx]

**Appendix L: Student SBIRT Patient Follow Up survey**

To be administered to patients during follow-up calls in conjunction with the follow-up guide / script to evaluate SBIRT impact on patient outcomes as a proxy measure for student proficiency in using SBIRT

Student SBIRT Patient Follow Up Survey

# Please complete the survey below

Date of SBIRT administration with patient:

Follow up call date:

Days since SBIRT administration (should be 14+ days)

Was a follow up call conducted? Yes No

Could the patient be reached via their method of preferred communication?

Yes No

If yes, please tell us about how this patient is doing and about any of the changes the patient has made since the SBIRT administration, if any.

What resource was the patient informed of or referred to?

Did the patient engage with the referred/informed resource?

Yes No

If yes, was the resource the patient engaged with helpful?

If no, what barriers did the patient experience precluding their engagement with the referred/informed resource, if any? Why didn’t they engage with the referred/informed resource?

Did the patient request medical advice?

Yes No

If yes, what medical advice was the patient requesting? (To be communicated with curriculum staff).

What is the best way for the curriculum team to contact the patient to follow up on their medical concerns? Please provide that information below.
