# Supplementary material for: Implementing a Screening, Brief Intervention, and Referral to Treatment Curriculum for Medical Students on their Emergency Department Rotation
Source: MedEdPORTAL. 2026 Jan 13;22:11569. doi: 10.15766/mep_2374-8265.11569 (PMC12796009; doi:10.15766/mep_2374-8265.11569)
Supplement: Supplementary file 1 — Medical Student MI-SBIRT Curriculum.pptxAlcohol Use Disorder Identification Test.docxDrug Abuse Screening Test (DAST-10).docxSBIRT Algorithm.docxSP Case Descriptions.docxSP Case.docxStudent OSCE Instructions.docxSubstance Use Facts Sheet.docxSBIRT Brief Intervention Card.docxSample OSCE Schedule.xlsxPatient Follow-Up Guide.docxStudent SBIRT Patient Follow-Up Survey.docxMI-SBIRT Attitudes and Preparedness Survey.docxPre- and Postcurriculum Assessment.docxStudent-Administered SBIRT Form.docxPost-SBIRT Patient Feedback Form.docxOSCE Score Sheet.docxExceeds Criteria.docxStudent Workflow and Protocol.docx [file mep_2374-8265.11569-s001.zip › M. MI-SBIRT Attitudes and Preparedness Survey.docx]

**Appendix M: MI/SBIRT Attitudes and Preparedness Pre-/Post-Curriculum Survey**

To be administered immediately prior to the didactics portion of the curriculum and at the end of the ED rotation

MI/SBIRT Attitudes and Preparedness Pre-/Post-Survey

# Use the scale below to rate your degree of agreement or disagreement with each of the following items.

Not sure but probably agree

Not sure but probably disagree

Strongly agree

Agree

Disagree

Strongly disagree

|  |  |  |  |  |  |
| --- | --- | --- | --- | --- | --- |
|  |  |  |  |  |  |
|  |  |  |  |  |  |
|  |  |  |  |  |  |
|  |  |  |  |  |  |
|  |  |  |  |  |  |
|  |  |  |  |  |  |
|  |  |  |  |  |  |
|  |  |  |  |  |  |

Motivational interviewing (MI) has a role in healthcare

Screening, brief intervention, referral to treatment (SBIRT) is efficient

SBIRT is satisfying to conduct

I feel comfortable conducting SBIRT

I feel prepared to discuss concerns regarding substance use disorder using MI techniques with patients

I feel prepared to discuss behavior change/modification using MI techniques with patients

SBIRT/MI is more effective than patient instructions in modifying behavior

I plan on using brief intervention/motivational interviewing techniques with patients in the future

SBIRT/MI training should be incorporated in medical student education
