# Supplementary material for: Implementing a Screening, Brief Intervention, and Referral to Treatment Curriculum for Medical Students on their Emergency Department Rotation
Source: MedEdPORTAL. 2026 Jan 13;22:11569. doi: 10.15766/mep_2374-8265.11569 (PMC12796009; doi:10.15766/mep_2374-8265.11569)
Supplement: Supplementary file 1 — Medical Student MI-SBIRT Curriculum.pptxAlcohol Use Disorder Identification Test.docxDrug Abuse Screening Test (DAST-10).docxSBIRT Algorithm.docxSP Case Descriptions.docxSP Case.docxStudent OSCE Instructions.docxSubstance Use Facts Sheet.docxSBIRT Brief Intervention Card.docxSample OSCE Schedule.xlsxPatient Follow-Up Guide.docxStudent SBIRT Patient Follow-Up Survey.docxMI-SBIRT Attitudes and Preparedness Survey.docxPre- and Postcurriculum Assessment.docxStudent-Administered SBIRT Form.docxPost-SBIRT Patient Feedback Form.docxOSCE Score Sheet.docxExceeds Criteria.docxStudent Workflow and Protocol.docx [file mep_2374-8265.11569-s001.zip › N. Pre- and Postcurriculum Assessment.docx]

**Appendix N: SBIRT Pre-/Post-Curriculum Assessment, Final Iteration**

To be administered immediately prior to the didactics portion of the curriculum and at the end of the ED rotation

Screening, Brief Intervention, and Referral to Treatment (SBIRT) Assessment Quiz

# Multiple Choice Questions

Please select the best response for each of the following questions.

1. Arturo is a 16-year-old adolescent presenting for his sports physical. During the encounter, he’s asked about substance use. He’s reassured that what he tells the pediatrician will be kept confidential unless it brings up serious safety concerns. Arturo shares that he’s recently been to a house party where alcohol was served and drank 4 or 5 drinks then spent the night.

Arturo’s drinking would be considered:

1. Non-excessive, as an excessive drinking level for Arturo is >5 drinks per occasion
2. Excessive, as an excessive drinking level for Arturo is >4 drinks per occasion
3. Excessive, as an excessive drinking level for Arturo is >3 drinks per occasion
4. Excessive, as an excessive drinking level for Arturo is >0 drinks per occasion
5. Jordyn is a 28-year-old woman who presents to her PCP complaining of “heartburn.” She endorses coughing at night and states it’s necessary for her to sleep with two pillows under her head to mitigate the “burning sensation.” She denies the usual triggers except for alcohol. She endorses drinking 6-8 shots a night when she goes out with her friends on weekends.

Based on her reported history, which screening tool would be most appropriate to investigate Jordyn’s alcohol consumption further?

A. ASSIST

B. AUDIT

C. SACS

D. DAST

E. CRAFFT

1. Shreya is a 27-year-old woman who is establishing care with a new PCP. During her social history, before she’s asked about her use of alcohol, nicotine, or other substances, her PCP states, “as part of routine health services, I ask all of my patients about behaviors that could impact their health, including the use of alcohol, nicotine, and other substances.”

Which “setting the stage” technique is the PCP using during this encounter?

1. Confidentiality
2. Normalizing
3. Addressing stigma
4. Beneficence
5. Jack is a 31-year-old man with hypertension who is establishing care with Dr. Rangwalla. Dr. Rangwalla asks Jack about his use of substances. Jack indicates he “enjoys a beer or two when watching the Dodgers play over the weekend.” If it’s not baseball season, he almost never consumes alcohol. He denies nicotine and other substances.

Based on this brief screen, what should Dr. Rangwalla’s next step be?

1. Reinforce healthy choices and leave the door open for further questions from Jack regarding his own use or a loved one’s use
2. Express concern, connect Jack’s alcohol use to his hypertension, and seek Jack’s perspective on the matter
3. Share the parameters of excessive drinking and ask Jack if he has any questions or thoughts regarding those parameters
4. Refer Jack to community resources that support individuals with a desire to reduce or eliminate their alcohol use
5. Malik is a 23-year-old man presenting to his university’s student health services for constipation. During the social history, Malik hesitantly reveals that he’s been using cocaine on a nightly basis for several months, and that he occasionally uses “percs” to “bring him back down.” He’s concerned about his substance use and its potential contribution to his constipation.

What stage of change is Malik most likely occupying?

1. Pre-contemplation
2. Contemplation
3. Preparation
4. Action
5. After going over the pros and cons of Joshua’s smoking during a follow-up visit concerning Joshua’s abdominal aortic aneurysm, Joshua’s vascular surgeon reflects, “on the one hand, smoking has been a stress reliever for you, but on the other hand, you’re worried about the impact your smoking is having on your aneurysm. So, where does that leave you?”

Which brief intervention technique is the surgeon exercising with Joshua?

A. Expressing empathy

B. Developing discrepancy

C. Rolling with resistance

D. Support self-efficacy

1. After listening to Xian’s account regarding her increasing use of Benadryl, including how she fights the urge to sleep to feel “loopy,” her therapist replies, “first, I want to thank you for sharing that. It takes a lot of courage and self-awareness to discuss these things. When you first experienced the ‘loopy sensation,’ what thoughts or emotions came up for you?”

Which two OARS motivational interviewing skills did the therapist just use? (circle two answers)

1. Open-ended questions
2. Affirmations
3. Reflective listening
4. Summarizing
5. Shannan is a 24-year-old non-binary person who presents to the ED with a soft tissue infection in their shoulder which arose shortly after they engaged in “skin popping” with heroin. They explain that skin popping allows them to preserve their veins. After establishing rapport, the physician asks, “could you share with me some of the things you know about skin popping or heroin use in general?”

Which brief intervention technique is the physician initiating?

1. Exploring ambivalence
2. Elicit-provide-elicit
3. Assessing readiness
4. Building an action plan
5. Which motivational interviewing congruent statement could you make to support Charles, a 68-year-old COPD patient, in his efforts to quit smoking and maintain his quit status, considering his recent hospitalization for an exacerbation, the fact that nicotine is no longer “relaxing” for him, his desire to enjoy his retirement and upcoming grandchild, and his past success with nicotine replacement therapy?
6. “Smoking is not relaxing anymore, and you are worried that, if you don’t quit smoking, you won’t get to enjoy your retirement or see your grandchildren grow up. You are ready to quit.
7. “You’ve been successful quitting for a full year before. I’m confident you can do this.”
8. “What do you think would help you quit and stay quit this time?”
9. All of the above
10. Davis is a 29-year-old woman with sleep apnea who presents to her PCP for a routine physical. She hasn’t been using her CPAP recently because it makes falling asleep difficult. Davis is asked what her motivation level is to explore options for safer sleep, including the resumption of her CPAP use. Davis indicates her motivation level is a 4 out of 10.

What is the most appropriate response from the physician given Davis’s motivation level?

1. What’s happened as a result of not sleeping well at night that’s concerned you?
2. Why choose a 4 and not a 3?
3. What would have to happen for you to go from a 4 to a 5?
4. What steps would be necessary to take to resume safe sleeping practices moving forward?

Key

1. D
2. B
3. B
4. A
5. B
6. B
7. A & B
8. B
9. D
10. B
