# Supplementary material for: Implementing a Screening, Brief Intervention, and Referral to Treatment Curriculum for Medical Students on their Emergency Department Rotation
Source: MedEdPORTAL. 2026 Jan 13;22:11569. doi: 10.15766/mep_2374-8265.11569 (PMC12796009; doi:10.15766/mep_2374-8265.11569)
Supplement: Supplementary file 1 — Medical Student MI-SBIRT Curriculum.pptxAlcohol Use Disorder Identification Test.docxDrug Abuse Screening Test (DAST-10).docxSBIRT Algorithm.docxSP Case Descriptions.docxSP Case.docxStudent OSCE Instructions.docxSubstance Use Facts Sheet.docxSBIRT Brief Intervention Card.docxSample OSCE Schedule.xlsxPatient Follow-Up Guide.docxStudent SBIRT Patient Follow-Up Survey.docxMI-SBIRT Attitudes and Preparedness Survey.docxPre- and Postcurriculum Assessment.docxStudent-Administered SBIRT Form.docxPost-SBIRT Patient Feedback Form.docxOSCE Score Sheet.docxExceeds Criteria.docxStudent Workflow and Protocol.docx [file mep_2374-8265.11569-s001.zip › O. Student-Administered SBIRT Form.docx]

**Appendix O: Student-Administered SBIRT Form**

To be provided to students immediately following their reported completion of SBIRT with a real patient in the ED so they may document their narrative experience

Student Administered SBIRT Form

## Please complete the survey below

Student Name:

Student EID:

Student’s Current Clerkship Block:

Student’s Current Clerkship Sub-Block:

Student Email:

## Please fill out the following fields to help us further evaluate the curriculum

Date of SBIRT administration:

Patient’s name (first name, last name):

Patient’s Medical Record Number (MRN):

Patient’s AUDIT score:

Patient’s DAST score:

Brief intervention performed? Yes No

If yes, patient reported motivation/readiness to change (1-10):

If yes, was a hand-off to social work performed? Yes No

If yes, was the patient referred to a community resource or was information provided regarding available community resources? Yes No

If yes, which resource was the patient referred to or made aware of?

Is the patient amenable to a two week &/or four week follow up call from you or the project team?

Yes, amenable to both a two week & four week follow-up call from you or the project team

Only amenable to a two week follow-up call from you or the project team

Only amenable to a four week follow-up call from you or the project team

No, not amenable to a two week nor four week follow-up call from you or the project team

Please note the patient’s preferred mode of contact and their contact information

If you used any of the following techniques during your conversation, please also indicate as much in the field below: building rapport, open-ended questions, affirmations, reflective listening, exploring ambivalence, elicit-provide-elicit, assessing readiness / readiness ruler.

Optional: you may also use this field to write comments or notes concerning the patient’s responses and/or reactions during or to the brief intervention. These notes may include anything you feel is pertinent or worth noting. Please remember to rely on these notes during your follow-up call with the patient, if they’re amenable to a follow-up call.
