# Supplementary material for: Implementing a Screening, Brief Intervention, and Referral to Treatment Curriculum for Medical Students on their Emergency Department Rotation
Source: MedEdPORTAL. 2026 Jan 13;22:11569. doi: 10.15766/mep_2374-8265.11569 (PMC12796009; doi:10.15766/mep_2374-8265.11569)
Supplement: Supplementary file 1 — Medical Student MI-SBIRT Curriculum.pptxAlcohol Use Disorder Identification Test.docxDrug Abuse Screening Test (DAST-10).docxSBIRT Algorithm.docxSP Case Descriptions.docxSP Case.docxStudent OSCE Instructions.docxSubstance Use Facts Sheet.docxSBIRT Brief Intervention Card.docxSample OSCE Schedule.xlsxPatient Follow-Up Guide.docxStudent SBIRT Patient Follow-Up Survey.docxMI-SBIRT Attitudes and Preparedness Survey.docxPre- and Postcurriculum Assessment.docxStudent-Administered SBIRT Form.docxPost-SBIRT Patient Feedback Form.docxOSCE Score Sheet.docxExceeds Criteria.docxStudent Workflow and Protocol.docx [file mep_2374-8265.11569-s001.zip › P. Post-SBIRT Patient Feedback Form.docx]

**Appendix P: Post-SBIRT Patient Feedback Form**

To be administered by clinical or project staff immediately following student administered SBIRT to a real patient to evaluate patient experience as a proxy measure of student proficiency in administering SBIRT

Post-SBIRT Patient Feedback Form

Patient Name:

Patient MRN:

Date completed:

# Please use the scale below to rate your degree of agreement or disagreement with each of the following items

**During your interaction with the medical student… (please put an “x” in each box that corresponds with your answer to each statement below)**

|  |  |  |  |  |  |  |
| --- | --- | --- | --- | --- | --- | --- |
|  |  |  |  |  |  |  |
|  |  |  |  |  |  |  |

The medical student worked together with you to come up with an action plan (if applicable)

Not sure but probably disagree

Disagree

Not sure but probably agree

N/a

Strongly disagree

Agree

Strongly agree

The medical student was curious about and respectful of your perspective

The medical student was prioritizing your values and your needs
