# Supplementary material for: Implementing a Screening, Brief Intervention, and Referral to Treatment Curriculum for Medical Students on their Emergency Department Rotation
Source: MedEdPORTAL. 2026 Jan 13;22:11569. doi: 10.15766/mep_2374-8265.11569 (PMC12796009; doi:10.15766/mep_2374-8265.11569)
Supplement: Supplementary file 1 — Medical Student MI-SBIRT Curriculum.pptxAlcohol Use Disorder Identification Test.docxDrug Abuse Screening Test (DAST-10).docxSBIRT Algorithm.docxSP Case Descriptions.docxSP Case.docxStudent OSCE Instructions.docxSubstance Use Facts Sheet.docxSBIRT Brief Intervention Card.docxSample OSCE Schedule.xlsxPatient Follow-Up Guide.docxStudent SBIRT Patient Follow-Up Survey.docxMI-SBIRT Attitudes and Preparedness Survey.docxPre- and Postcurriculum Assessment.docxStudent-Administered SBIRT Form.docxPost-SBIRT Patient Feedback Form.docxOSCE Score Sheet.docxExceeds Criteria.docxStudent Workflow and Protocol.docx [file mep_2374-8265.11569-s001.zip › Q. OSCE Score Sheet.docx]

**Appendix Q: OSCE Score Sheet**

To be used by MINT coaches to score student OSCE encounters and guide real-time feedback sessions; scores are not to be shared with students

SBIRT

Coding Form

Coder/coach:_______________ Date of Session:_________________

Med Student:_______________

Interview # (circle one): 1 2 3

GLOBAL RATINGS

MI Spirit (autonomy, collaboration, evocation) 1 2 3 4 5 6 7

Low High

Empathy 1 2 3 4 5 6 7

Low High

BEHAVIOR COUNTS

Giving information (tally): _________________________________________________________

MI adherent (tally): _______________________________________________________________

(permission, affirm, support, choice)

MI non-adherent (tally): __________________________________________________________

(advising, confronting, persuading, teaching)

Question

Open (tally): ______________________________________________________________

Closed (tally): _____________________________________________________________

Total Questions (number): _____________

Reflection

Simple (tally): _____________________________________________________________

Complex (tally): ___________________________________________________________

Total Reflections (number): ______________

NOTES:

**How to score:**

**MI adherent:**

- Place a single tally per incident of student requested permission to discuss substance use, to offer feedback, to offer patient specific education regarding the standardized patient’s (SP) substance use.
- Place a single tally per incident of student provided affirmations in response to SP accounts during their conversation.
- Place a single tally per incident of the student’s offering of choices guiding the SP’s decision-making during their conversation.
- Place a single tally per incident of student offered unconditional support / nonjudgment of SP decision-making regarding their substance use, reinforcing respect for SP autonomy.

**MI non-adherent:**

- Place a single tally per incident of student offered unsolicited advice concerning the SP’s substance use.
- Place a single tally per incident of student confrontation regarding SP substance use, including use of the words “you need/have to,” or the unsolicited suggestion that continued use would result in negative consequences.
- Place a single tally per incident of student attempted persuasion to induce behavior change in the SP, eg quid pro quo or offering of rewards.
- Place a single tally per incident of student attempted teaching or provision of education around substances and substance use without permission.

**Calculating composite score:**

Global ratings have a 1:1 score ratio, eg a global rating of 7 for MI Spirit corresponds to 7 points.

Each Giving Information tally is worth 1 point.

Each MI adherent tally is worth 1 point.

Each MI non-adherent tally is worth -0.5 points.

Each Open-Ended Question asked is worth 1 point.

Each Closed Ended Question asked is worth -0.5 points.

Each Reflection, simple or complex, is worth 1 point.

A composite score is equal to the sum of all points.
