# Supplementary material for: Implementing a Screening, Brief Intervention, and Referral to Treatment Curriculum for Medical Students on their Emergency Department Rotation
Source: MedEdPORTAL. 2026 Jan 13;22:11569. doi: 10.15766/mep_2374-8265.11569 (PMC12796009; doi:10.15766/mep_2374-8265.11569)
Supplement: Supplementary file 1 — Medical Student MI-SBIRT Curriculum.pptxAlcohol Use Disorder Identification Test.docxDrug Abuse Screening Test (DAST-10).docxSBIRT Algorithm.docxSP Case Descriptions.docxSP Case.docxStudent OSCE Instructions.docxSubstance Use Facts Sheet.docxSBIRT Brief Intervention Card.docxSample OSCE Schedule.xlsxPatient Follow-Up Guide.docxStudent SBIRT Patient Follow-Up Survey.docxMI-SBIRT Attitudes and Preparedness Survey.docxPre- and Postcurriculum Assessment.docxStudent-Administered SBIRT Form.docxPost-SBIRT Patient Feedback Form.docxOSCE Score Sheet.docxExceeds Criteria.docxStudent Workflow and Protocol.docx [file mep_2374-8265.11569-s001.zip › R. Exceeds Criteria.docx]

**Appendix R: Exceeds Criteria**

To be reviewed during the didactic and made available to students during the curriculum administration so they’re aware of the specific actions they must take in the context of our curriculum to qualify for “exceeds expectations” in the Health Equity and Interprofessional Care categories of their clinical evaluation

**SBIRT ADMINISTRATION “EXCEEDS” CRITERIA**

**Categories available to “Exceed Expectations” as part of 10/15 needed to honor your Emergency Medicine Clerkship:**

- Health Equity
- Interprofessional Care

**SBIRT Completion Criteria**

1. Note patient name.
2. Identify risky use via AUDIT / DAST or provide history details which otherwise indicate / imply high AUDIT / DAST scores.
   1. Alternatively, may identify opportunity to counsel re: diet / exercise / medication adherence, etc.
3. Engage in brief intervention and note **at least 2 techniques** that were used during the interaction, eg “exploring ambivalence”; “elicit-provide-elicit”; “assessing readiness / readiness ruler.” **One of the techniques used must be “assessing readiness / readiness ruler.”**
4. Inform social work of your work so that they may administer the post-SBIRT survey with the patient and / or follow up with them to provide resources.*
5. Complete Student Administered SBIRT Form and note completion of the above.

*Note, if SW does not administer the survey, or if patient refuses to see SW even to just complete the survey, this will not count against the students’ completed SBIRT. If social work is not available, please notify a member of the curriculum team.
