# Supplementary material for: Implementing a Screening, Brief Intervention, and Referral to Treatment Curriculum for Medical Students on their Emergency Department Rotation
Source: MedEdPORTAL. 2026 Jan 13;22:11569. doi: 10.15766/mep_2374-8265.11569 (PMC12796009; doi:10.15766/mep_2374-8265.11569)
Supplement: Supplementary file 1 — Medical Student MI-SBIRT Curriculum.pptxAlcohol Use Disorder Identification Test.docxDrug Abuse Screening Test (DAST-10).docxSBIRT Algorithm.docxSP Case Descriptions.docxSP Case.docxStudent OSCE Instructions.docxSubstance Use Facts Sheet.docxSBIRT Brief Intervention Card.docxSample OSCE Schedule.xlsxPatient Follow-Up Guide.docxStudent SBIRT Patient Follow-Up Survey.docxMI-SBIRT Attitudes and Preparedness Survey.docxPre- and Postcurriculum Assessment.docxStudent-Administered SBIRT Form.docxPost-SBIRT Patient Feedback Form.docxOSCE Score Sheet.docxExceeds Criteria.docxStudent Workflow and Protocol.docx [file mep_2374-8265.11569-s001.zip › S. Student Workflow and Protocol.docx]

**Appendix S: Student Workflow & Protocol**

To be reviewed during the didactic and provided to students to guide their actions throughout the curriculum

Med Student MI/SBIRT Project

# Medical Student Workflow & Protocol

1. Complete MI/SBIRT Attitudes and Preparedness Pre-Survey and SBIRT Pre-Assessment
2. Participate in SBIRT & SW/CM hand-off training with Dr. Velasquez during ED clerkship orientation.
3. Participate in simulated practice via formative OSCE
4. **During ED shift, identify patient**
5. Engage with patient, take history and physical as per usual. During history, **perform prescreen via social history** (eg substances hx from HEADDSS framework).
6. **Present patient to attending**, write note, etc.
7. If patient reported substance use during social history, or if there’s strong suspicion of substance use disorder based on history/physical, return to patient room to **perform SBIRT.**
   1. Time permitting, go through AUDIT and / or DAST and record score
   2. Based on score (4 or greater on AUDIT, 1 or greater on DAST) proceed with brief intervention
   3. If patient is interested in exploring available resources, offer a Social Work consult
   4. Ask patient if they’d like a follow up call at 4 weeks
8. **Consult ED SW/CM regarding patient if indicated**. Provide MRN and brief background as to why patient is hospitalized. Give reason for consult including DAST & AUDIT scores (if possible), patient’s motivation score (1-10), and insurance status. Be sure to follow up with SW regarding referral (where was patient referred/what resource was the patient informed of?).
9. **Regardless of whether SW consult indicated, please call and ask SW to administer post-SBIRT survey to patient** (identify yourself as medical student participating in SBIRT curriculum)
10. **Complete RedCap Student-Administered SBIRT survey**.
11. Debrief with OSCE Coach
12. Complete “SBIRT Post-Assessment” quiz on REDCap
13. Complete SBIRT Attitudes and Preparedness Post-Survey on REDCap
