# Supplementary material for: Implementing a Screening, Brief Intervention, and Referral to Treatment Curriculum for Medical Students on their Emergency Department Rotation
Source: MedEdPORTAL. 2026 Jan 13;22:11569. doi: 10.15766/mep_2374-8265.11569 (PMC12796009; doi:10.15766/mep_2374-8265.11569)
Supplement: Supplementary file 1 — Medical Student MI-SBIRT Curriculum.pptxAlcohol Use Disorder Identification Test.docxDrug Abuse Screening Test (DAST-10).docxSBIRT Algorithm.docxSP Case Descriptions.docxSP Case.docxStudent OSCE Instructions.docxSubstance Use Facts Sheet.docxSBIRT Brief Intervention Card.docxSample OSCE Schedule.xlsxPatient Follow-Up Guide.docxStudent SBIRT Patient Follow-Up Survey.docxMI-SBIRT Attitudes and Preparedness Survey.docxPre- and Postcurriculum Assessment.docxStudent-Administered SBIRT Form.docxPost-SBIRT Patient Feedback Form.docxOSCE Score Sheet.docxExceeds Criteria.docxStudent Workflow and Protocol.docx [file mep_2374-8265.11569-s001.zip › I. SBIRT Brief Intervention Card.docx]

**Appendix I: SBIRT Brief Intervention Card**

To be reviewed during the didactic portion and used to discuss AUDIT/DAST scores with patients ahead of transition to SBIRT brief intervention


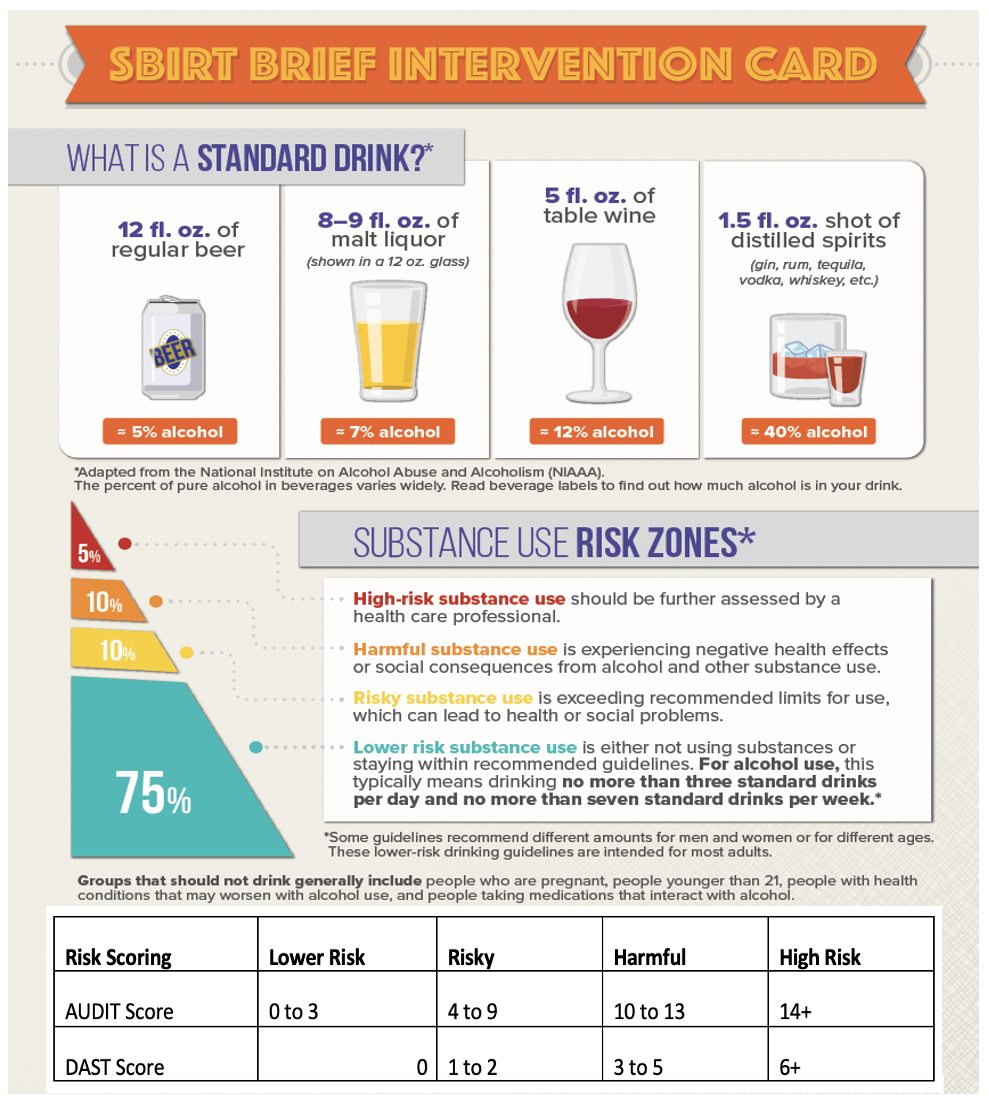


***Risk:** meaning, in our research, we’ve found that those with scores in these ranges have varying *likelihoods* of developing substance use disorder/becoming addicted to a substance. It is not guaranteed, everyone is different.

Adapted from the New York State Office of Addiction Services and Supports, “SBIRT Brief Intervention Card”

Original image by New York State Office of Addiction Services and Supports, retrieved from <https://oasas.ny.gov/> on 9/5/2023. **Image is in the public domain**.

**New York State Office of Addiction Services and Supports (OASAS).** SBIRT: Practitioner Reference Card. New York State OASAS; 2023. Accessed June 22, 2025. <https://oasas.ny.gov/system/files/documents/2023/04/oasas-1-practitioner_card.pdf>. **Image is in the public domain**.
