# Supplementary material for: Implementing a Screening, Brief Intervention, and Referral to Treatment Curriculum for Medical Students on their Emergency Department Rotation
Source: MedEdPORTAL. 2026 Jan 13;22:11569. doi: 10.15766/mep_2374-8265.11569 (PMC12796009; doi:10.15766/mep_2374-8265.11569)
Supplement: Supplementary file 1 — Medical Student MI-SBIRT Curriculum.pptxAlcohol Use Disorder Identification Test.docxDrug Abuse Screening Test (DAST-10).docxSBIRT Algorithm.docxSP Case Descriptions.docxSP Case.docxStudent OSCE Instructions.docxSubstance Use Facts Sheet.docxSBIRT Brief Intervention Card.docxSample OSCE Schedule.xlsxPatient Follow-Up Guide.docxStudent SBIRT Patient Follow-Up Survey.docxMI-SBIRT Attitudes and Preparedness Survey.docxPre- and Postcurriculum Assessment.docxStudent-Administered SBIRT Form.docxPost-SBIRT Patient Feedback Form.docxOSCE Score Sheet.docxExceeds Criteria.docxStudent Workflow and Protocol.docx [file mep_2374-8265.11569-s001.zip › _Educational Summary Report_11569.pdf]

MedEdPORTAL® | The AAMC Journal of Teaching and Learning Resources

Original Publication

Open Access

Implementing a Screening, Brief Intervention, and Referral to Treatment Curriculum for Medical Students on their Emergency Department Rotation

Samuel Burr, MD, MSHCT*, Samantha Shulhan, Bridget Fitzgerald, MS, Uma Jacobs, Alanna Boulton, MSHS, Drew Coyne, MD, Lloyd Berg, PhD, Kirk Von Sternberg, PhD, John Weems, MD, Jacki Hecht, RN, MSN, Patrick Kennedy, MSSW, Mary M. Velasquez, PhD

*Corresponding author: sburr@jpshealth.org

Abstract

Introduction: The screening, brief intervention, and referral to treatment (SBIRT) approach is an evidence-based tool that combines standardized screening for unhealthy or risky alcohol and drug use with principles of motivational interviewing to promote behavior change and connect patients with the appropriate treatment and recovery support services. There is an increased demand for health care students and providers to be trained in SBIRT. We developed a curriculum to improve medical students' attitudes toward and proficiency in administering SBIRT. **Methods:** The curriculum was deployed as part of the emergency department clerkship of an undergraduate medical education program at an urban, safety net academic medical center. The content and structure, developed with input from medicine, nursing, and social work educators, consists of a 1-hour didactic session, three rounds of formative OSCE encounters, and one SBIRT delivery in the emergency department. Students were evaluated on their attitudes, sense of preparedness, and practical understanding of SBIRT. **Results:** Fifty-six medical students participated in the curriculum. There were significant differences between students' pre- and postcurriculum attitudes and preparedness scores ($p < .001$) and knowledge scores ($p = .002$), and in OSCE scores between the first and third standardized patient encounter ($p = .03$). **Discussion:** This curriculum significantly impacted medical students' attitudes and knowledge regarding SBIRT and motivational interviewing techniques. Widespread implementation of similar curricula could equip future physicians with the skills to implement evidence-based substance use screening and intervention into their practice.

Keywords
SBIRT, Motivational Interviewing, OSCE, Interprofessional Education, Substance Use Disorders, Trauma-Informed Care, Emergency Medicine, Clinical Skills Assessment/OSCEs, Clinical/Procedural Skills Training, Simulation, Standardized Patient

Educational Objectives

By the end of this activity, learners will be able to:

1. Describe principles of motivational interviewing and screening, brief intervention, and referral to treatment (SBIRT), including risk stratification, normalization, addressing stigma, excessive drinking parameters, stages of change, open-ended questions, affirmations, reflections, and motivation-enhancing techniques.
2. Demonstrate improved attitudes and preparedness in medical students to administer SBIRT in clinical settings.
3. Practice administering motivational interviewing and SBIRT with standardized patients.
4. Apply techniques of motivational interviewing and SBIRT with a patient during their emergency medicine rotation.

Citation:
Burr S, Shulhan S, Fitzgerald B, et al. Implementing a screening, brief intervention, and referral to treatment curriculum for medical students on their emergency department rotation. *MedEdPORTAL*. 2026;22:11569. https://doi.org/10.15766/mep_2374-8265.11569

Introduction

Screening, brief intervention, and referral to treatment (SBIRT) is an efficient and evidence-based approach grounded in motivational interviewing (MI) principles for implementing patient screening, providing educational materials, raising self-awareness, and enhancing motivation to engage in behavior change.[1] There is substantial research demonstrating the effectiveness of SBIRT in reducing risky alcohol consumption, and accumulating evidence supporting the effectiveness of SBIRT in reducing risky drug use.[2-5] The Centers for Medicare and Medicaid Services recognizes SBIRT as a recommended approach to systematically screen people who may not otherwise

Copyright © 2026 Burr et al. This is an open-access publication distributed under the terms of the Creative Commons Attribution license.

seek substance use help and offer treatment services that reduce health costs, drug and alcohol use severity, and the number of patients who go without specialized treatment.[6] In the United States, there is a national push to develop curricula to train health care professionals, including medical residents and physicians, to become proficient in administering SBIRT.[7] This has resulted in curriculum innovation, including SBIRT or MI training implementation into health professions education.[8-14]

OSCE and role-playing activities have previously been incorporated into successful approaches to health professions SBIRT and MI teaching.[9-11,13,14] Student performance in tobacco cessation counseling and SBIRT was observed to be favorable following OSCE activities, standardized patient (SP) instruction, and participation in role-playing versus didactic or module-based approaches alone.[15] Role-playing is an approach supported by the American Heart Association, given its effectiveness in preparing medical students for lifestyle counseling when combined with traditional didactic approaches.[16] More generally, the OSCE has become the standard assessment method in medical education due to its effectiveness in evaluating key clinical skills through direct observation and in preparing medical students for clinical rotations.[17]

Our institution introduces MI techniques to first-year medical students (MS1s) by providing a 3-hour interactive lecture. While helpful, it is limited and does not cover SBIRT. To bridge these gaps, we developed an expanded SBIRT and MI curriculum for second-year medical students (MS2s) during their emergency department (ED) clerkship rotation. This curriculum aims to boost student readiness to administer SBIRT through a program of advanced training, practice with SPs involving real-time feedback, and encouragement to administer SBIRT on a patient in the ED.

Methods

We adopted the lecture materials and OSCE format materials developed by senior faculty in the Department of Family Medicine at Baylor College of Medicine and the Health Behavior Research and Training Institute at the University of Texas at Austin (UT Austin). We negotiated the retained and added components to this source material with faculty from the Steve Hicks School of Social Work (SHSSW) at UT Austin. We offered the curriculum to all MS2s during their 4-week emergency medicine clerkship, which took place in 53- and 27-bed EDs in urban, safety net academic medical centers in Texas. Participation was voluntary; however, it was incentivized through indications on final clinical evaluations.

Curriculum Activities

This three-part curriculum consisted of a 1-hour didactic session, a formative OSCE, and one SBIRT session delivered to students during their service in the ED. The didactic portion consisted of a presentation (Appendix A) led by an interprofessional panel, consisting of a third- or fourth-year medical student and a behavioral scientist, nurse, or psychologist. The upper-level medical student discussed validated screening tools, including the Alcohol Use Disorder Identification Test (AUDIT; Appendix B) and Drug Abuse Screening Test (DAST) (Appendix C), how to address stigma associated with substance use, and how to provide feedback to patients, including safe drinking level guidelines as defined by the United States Preventative Task Force. The behavioral scientist, nurse, and psychologist are experienced trainers belonging to the Motivational Interviewing Network of Trainers (MINT).[18] They, in turn, trained the students in MI techniques to employ during SBIRT interviews, such as the stages of change, MI techniques outlined in the OARS Model (open-ended questions, affirmations, reflections, summarization), and the Readiness Ruler for promoting readiness to change talk. Finally, we provided an SBIRT algorithm (Appendix D) to guide our students through a typical interview for use during OSCEs or the delivered SBIRT administration during their ED rotation.

OSCEs took place in simulation centers on the Dell Seton Medical Center (Dell Med) and UT School of Nursing campuses and consisted of three 12-minute interviews with SPs. The SPs were volunteer graduate students from health profession schools at UT Austin who had previously completed advanced coursework in behavioral health and MI. Each SP was given one of three available character descriptions with associated AUDIT/DAST scores (Appendices E and F) and OSCE logistics via email. They were instructed to improvise their responses to students' SBIRT administration based on their character descriptions and to demonstrate willingness to change. We developed each OSCE case and character description collaboratively between students from Dell Med and faculty from the SHSSW. Cases included SPs of various ages and mock medical conditions that commonly present to the ED. Each case included risky levels of drug or alcohol use. We instructed students to review the SP's AUDIT or DAST score, discuss their risk category, administer a brief intervention utilizing MI techniques, and determine the appropriate next steps based on the SP's readiness to change during each case (Appendix G). During their encounters, we also encouraged students to use informational materials including the SBIRT algorithm (Appendix D), a Substance Use Fact Sheet (Appendix H), and a Brief Intervention Card developed by the Office of Addiction Services and Supports (Appendix I). We

Copyright © 2026 Burr et al. This is an open-access publication distributed under the terms of the Creative Commons Attribution license.

monitored students' performances virtually and our MI coaches scored them according to their use of OARS MI techniques during each OSCE. Virtual observation was made possible via live-streaming audio and video captured in simulated hospital rooms to laptops monitored by our coaches. Following each encounter, 10 minutes of individualized formative feedback was provided by trainers from MINT. We typically ran two simulations at once, each followed by feedback, requiring three to four actors (one for each case), and two MI coaches. A sample schedule is provided in Appendix J.

SBIRT Delivery

After completing the didactic and OSCE portions of the curriculum, we asked students to identify an appropriate patient presenting to the ED at either Dell Med or the Seton Medical Center in Austin, Texas, with whom to administer the SBIRT intervention. Patients who were actively intoxicated, in distress from withdrawing from a substance, combative, hemodynamically unstable, experiencing altered mental status, or unable to talk due to oxygen requirements were ineligible for SBIRT intervention. Student-patient interactions were supervised by ED residents and attending physicians. Upon completion of SBIRT administration with at least one patient, students coordinated with the patient and ED social workers to determine appropriate referral or provision of information regarding available resources according to the patient's insurance status.

We attempted follow-up calls to patients who received the SBIRT intervention during their ED encounter, at 2 weeks and 4 weeks after discharge, if they consented to follow-up contact. We planned to ask patients both open- and closed-ended questions about any behavior changes, whether they followed up with the referred resources, and any barriers they encountered. These follow-up calls were performed by either a member of the project team or the student who had administered the SBIRT. We provided a script to students to facilitate follow-up call conversations and to provide them with appropriate responses to patient queries around medical care or care coordination (Appendix K). These questions were to be noted and emailed to the project team, including an attending physician, to review and address with the patient separately. In addition, patient responses to follow-up calls were to be separately recorded in a Student SBIRT Patient Follow Up Survey (Appendix L). If patients changed their behavior or engaged with the resources to which they were referred, this was considered an additional indicator of student SBIRT administration proficiency.

Patients were free to refuse SBIRT administration from students. Regardless of patient participation in students' SBIRT delivery, patients received standard of care for their chief presenting symptoms and any additional medical concerns noted during evaluation. The UT Austin Institutional Review Board deemed this activity non-human subjects research.

Learner Assessment

We conducted learner assessments across Levels 1 through 3 of Kirkpatrick's learning evaluation model.[19] At Level 1, we assessed student attitudes and preparedness in using MI techniques and administering SBIRT, through self-reported pre- and postcurriculum surveys (Appendix M). Level 2 focused on evaluating students' knowledge acquisition from pre- to postcurriculum, using multiple-choice assessments covering lecture topics (Appendix N). For Level 3, we measured student behavioral changes by assessing score trends across three OSCEs, the completion of a Student Administered SBIRT Form (Appendix O) after the student had administered a simulated SBIRT interview, and patient-reported outcomes via the Post-SBIRT Patient Feedback Form (Appendix P). We used all assessments as curriculum evaluation methods. The scores on all evaluation methods did not impact clerkship grades.

We developed pre- and postcurriculum attitudes and preparedness surveys (Appendix M) in collaboration with faculty members of the Department of Internal Medicine at Dell Med. These surveys, consisting of nine items, assessed students' attitudes toward substance use disorder and sense of preparedness around MI and SBIRT administration. We scored each item on a 6-point Likert scale (scores ranging from 1 to 6; 1 = *strongly disagree*, 6 = *strongly agree*) based on the extent to which the student agreed with each statement. We scored surveys by summing the scores for all items; these summed scores ranged from 9 points to 54 points, with higher scores indicating increased preparedness and more positive attitudes.

We developed the pre- and postcurriculum knowledge assessments (Appendix N) using an iterative approach in collaboration with faculty members at the SHSSW and Dell Med. We designed them to assess students' knowledge of didactic topics, including defining severity of substance use disorder, stages of change, and MI techniques.

We evaluated the OSCE cases with a score sheet derived from the MINTs' resident physician training (Appendix Q), which is an adaptation of the Motivational Interviewing Treatment Integrity Coding System 4.[20] MINT coaches graded students based on their students' exhibition of MI spirit (e.g., partnership, acceptance, and compassion), empathy during the SP encounter, and the number of times they utilized MI techniques and provided

Copyright © 2026 Burr et al. This is an open-access publication distributed under the terms of the Creative Commons Attribution license.

open-ended feedback. We analyzed student performance by comparing the score change from round one to round three. In between sessions, MINT trainers offered formative feedback to improve students' comfort and skill with interviewing patients.

After a student administered an SBIRT interview with a patient in the ED, they completed the Student Administered SBIRT Form (Appendix O), which documented AUDIT/DAST scores, MI techniques used, referrals to social work, and a narrative summary of the interview. In addition, students referred unit case management staff to administer the Post-SBIRT Patient Feedback Form (Appendix P). We developed this survey in collaboration with Dell Med faculty members. The survey contained three items relating to the students' endorsement of MI spirit, as defined by MINT during the SBIRT administration from the patient's perspective.[14] We scored the patient survey responses on a 6-point Likert scale based on greater agreement with each survey item (1 = *strongly disagree*, 6 = *strongly agree*). The maximum achievable score on the patient survey was 18. To increase student engagement with SBIRT delivery in the ED, students who completed this portion of the curriculum were able to receive credit on their final clinical evaluation (Appendix R). To that end, given that multiple steps were required to proceed through our curriculum and claim credit per our incentive, we provided students with a comprehensive workflow document (Appendix S).

Statistical Analysis

We used the Wilcoxon signed-rank test as a nonparametric statistical test for comparing pre- and postcurriculum evaluation results, as well as OSCE scores. Given the small sample size throughout each evaluation, we used nonparametric testing for statistical analysis, with the significance level set at $p < .05$. We generated all analyses using Microsoft Excel.

Results

A total of 56 MS2s participated in the curriculum, with 20 administering formal SBIRTs in the ED.

We excluded from the survey analysis students who did not complete both the precurriculum and postcurriculum evaluations (knowledge assessment and other surveys). Of the 56 students, 41 MS2s completed both the pre- and postcurriculum surveys on attitudes and preparedness. Among these respondents, the median total score of students' attitudes and preparedness increased by 16% ($SD = 9\%$).

Due to question length and difficulty, we revised the knowledge assessment twice, with the third version leading to more consistent results. Students who completed the first two versions of the knowledge assessment ($n = 22$) or who did not complete both rounds of the third version ($n = 10$) were excluded. Of the remaining 24 students, the median precurriculum knowledge assessment score was 67%, while the median postcurriculum knowledge assessment score was 78%. Student's median total knowledge assessment score increased by 11% ($SD = 15\%$).

Of the 56 students who participated in the SBIRT curriculum, 37 (66%) completed the OSCE portion. Students' median OSCE score increased by 19% ($SD = 35\%$) from the first to the third SP encounter.

In comparing results of the pre- and postcurriculum assessments using the Wilcoxon signed-rank test (Table), we found that there was a statistically significant difference in median scores between the pre- and postcurriculum attitudes and preparedness survey ($p < .001$) and knowledge assessment ($p = .002$), and a statistically significant increase in median scores between the first and third round of OSCE scores ($p = .03$).

Of the 20 students who administered an SBIRT interview to a patient, 12 (60%) provided a narrative of their SBIRT interview, including MI techniques used. Techniques used by students included assessing readiness/Readiness Ruler, open-ended questions, affirmations, elicit-provide-elicit, building rapport, reflective listening, and exploring ambivalence. Twenty-four percent of students utilized assessing readiness and/or the

Table. Changes in Learner Assessment Scores Before and After Participation in the SBIRT and MI Curriculum

Evaluation Measure	*N* (Paired Differences)	Precurriculum *Mdn* Score	Postcurriculum *Mdn* Score	*W* Statistic	*Z* Score	p^a	Effect Size
Attitudes and preparedness survey[b]	41	38.0	48.0	3.0	−5.54	<.001	0.87
Knowledge assessment[c]	20	0.7	0.8	23.0	−3.06	.002	0.68
OSCE score[d]	35	20.5	22.5	182.5	−2.17	.03	0.37

Abbreviations: MI, motivational interviewing; SBIRT, screening, brief intervention, and referral to treatment.
[a]Pre- to postcurriculum comparison by Wilcoxon signed-rank test.
[b]Attitudes and preparedness survey scored out of a maximum of 54 points.
[c]Knowledge assessment scored out of a maximum of 1.0 points.
[d]OSCE scored out of unlimited number of points or counts.

Copyright © 2026 Burr et al. This is an open-access publication distributed under the terms of the Creative Commons Attribution license.

Readiness Ruler, 17% utilized reflective listening, open-ended questions, and building rapport, 11% utilized affirmations, 7% of students utilized exploring ambivalence and elicit-provide-elicit techniques (Figure).

Of the 20 patients who underwent SBIRT administration, six requested follow-up phone calls. Three of those six provided a phone number with which to contact them, but we could not reach them via phone at their preferred follow-up time.

Discussion

Through the development and implementation of this novel integrated medical student SBIRT curriculum, we have demonstrated an increase in knowledge and proficiency in SBIRT administration and MI techniques. Collectively, our attitudes and preparedness survey, knowledge assessment, and OSCE data indicate that formal MI and SBIRT training in clerkship medical education of MS2s can significantly impact the students' proficiency and comfort with SBIRT and MI. There was attrition demonstrated in students' pursuit of SBIRT opportunities in the ED, and therefore our ability to claim student proficiency based on their narrative experiences and patient outcome data is limited. It is questionable whether the ED is the best milieu for students to leverage their SBIRT training, especially given the preexisting cognitive and logistical demands of emergency medicine for MS2s and our curriculum's designation as optional. The attitudes and preparedness survey results, however, suggest that exposing medical students to MI and SBIRT techniques early in their careers may improve their comfort level with addressing substance use with patients. When equipped with SBIRT, and after gaining proficiency with the SBIRT simulation, learners are more likely to engage in these sensitive discussions than before their training. This could have positive implications for patient outcomes.

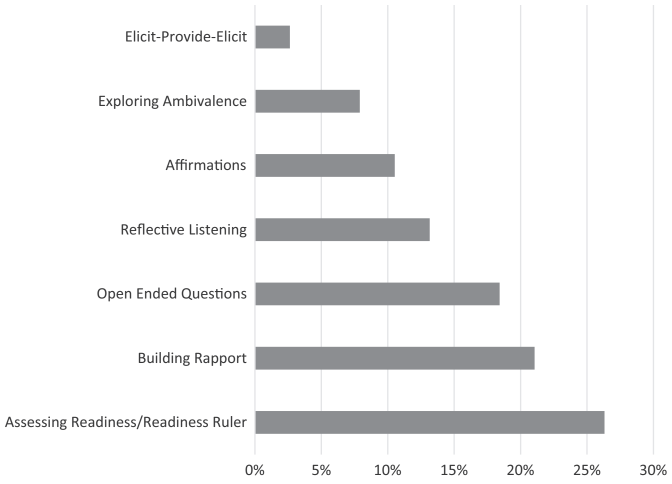

Figure. Motivational interviewing (MI) techniques used during the screening, brief intervention, and referral to treatment (SBIRT) curriculum. Twenty students administered SBIRT to a patient, and 12 (60%) provided a narrative of their SBIRT interview, including MI techniques used.

Challenges and Limitations

Educators considering replication of our curriculum should consider the following challenges, including their associated iterations and suggestions for overcoming them. Challenges included revising knowledge assessments, addressing large standard deviations in OSCE score changes, encouraging SBIRT administration in the ED, incentivizing general participation in a noncompulsory curriculum, and acquiring patient follow-up data.

Our pre- and postcurriculum knowledge assessments underwent two revisions due to question difficulty and length. The original assessment questions required students to recall information they would not need to memorize for SBIRT administration. The revised assessment posed questions with responses that reflected students' practical understanding. These revisions halved the student assessment scores used in our data analysis, which limits the results' statistical power to claim definitive proficiency. We are reassured that student declarative understanding is corroborated by OSCE score improvements over time, which is supported by a larger sample size. Replications of this curriculum should evaluate whether there is a correlational relationship between OSCE and assessment scores.

The OSCE score changes between first and third SP encounters exhibited large standard deviations. Possible explanations include unclear student case instructions, SP acting variability, and interrater reliability for MI spirit and empathy scores—which did not have a defined rubric—and differences in rater ability to correctly tally MI technique "counts." Solutions include eliciting feedback from low-performing students on how to improve our instructions, standardizing actor performances through more robust training or protocols, creating a rubric for scoring ambiguous items during the OSCE, and recording simulations to improve accuracy in tallying "counts."

Although not formally measured, general participation in the curriculum was limited by the curriculum's optional nature, requiring different strategies to encourage student engagement. An implied solution for replicating similar curricula would be to make it compulsory or available via elective opportunities. However, we adapted a few strategies to increase participation. We informed ED attending physicians of our pilot program to

Copyright © 2026 Burr et al. This is an open-access publication distributed under the terms of the Creative Commons Attribution license.

increase their awareness of it to facilitate student participation. We incentivized students to perform SBIRT in the ED by offering to boost their clerkship evaluations according to a standardized rubric (Appendix R). Despite these efforts, a challenge that persisted was that students cited difficulty identifying patients who would score high enough on AUDIT/DAST to warrant brief interventions but who were also not so intoxicated that their participation was excluded. To help with this patient identification barrier, we recruited ED social workers to refer appropriate patients to students, but this effort was challenged by ED social worker bandwidth.

It was difficult to obtain patient follow-up data from the patient satisfaction surveys and the follow-up phone calls. Patient satisfaction survey collection suffered due to coordination issues between students and ED social workers, and patients not answering follow-up phone calls. Consequently, this data did not yield meaningful results and was excluded from our analysis. Further, follow-up calls could have been confounded by bias when completed by administering students if patients decided to overreport SBIRT impact to protect students' feelings.

Given our challenges related to student participation in the ED and patient survey data collection coordination, this curriculum would be better administered as an elective opportunity. While this would limit the number of participants, student self-selection would reduce attrition. A focused elective in which students respond to inpatient or ED SBIRT consults would lessen cognitive load. Coordination of patient outcome data could be improved by reassigning data collection to clinical supervisors rather than social workers with competing duties.

This curriculum relies heavily on a strong partnership with the SHSSW, the Health Behavior Research and Training Institute, and its trainers from the MINT program. Further, this project's funding to pay MINT- certified MI coaches during our OSCE was contingent on trainees' use of SBIRT in inpatient settings. If grant funding stipulations for this work were more flexible, this curriculum could be replicated by other institutions anywhere.

Further Directions

Moving forward, given the experienced challenges, our aim is to integrate this vetted curriculum into a preexisting addiction medicine elective concurrently developed by internal medicine residents at Dell Med. There is expressed interest in incorporating our curriculum into this elective, which could be extended to MS2s and MS4s who have elective time available in their respective years. Formal integration would achieve access to allocation of instructional funds from the Department of Internal Medicine to supplant the grant funding the curriculum relies on.

Appendices

A. Medical Student MI-SBIRT Curriculum.pptx
B. Alcohol Use Disorder Identification Test.docx
C. Drug Abuse Screening Test (DAST-10).docx
D. SBIRT Algorithm.docx
E. SP Case Descriptions.docx
F. SP Case.docx
G. Student OSCE Instructions.docx
H. Substance Use Facts Sheet.docx
I. SBIRT Brief Intervention Card.docx
J. Sample OSCE Schedule.xlsx
K. Patient Follow-Up Guide.docx
L. Student SBIRT Patient Follow-Up Survey.docx
M. MI-SBIRT Attitudes and Preparedness Survey.docx
N. Pre- and Postcurriculum Assessment.docx
O. Student-Administered SBIRT Form.docx
P. Post-SBIRT Patient Feedback Form.docx
Q. OSCE Score Sheet.docx
R. Exceeds Criteria.docx
S. Student Workflow and Protocol.docx

All appendices are peer reviewed as integral parts of the Original Publication.

Samuel Burr, MD, MSHCT: First-Year Resident Physician, Department of Family Medicine, John Peter Smith Health Network, ORCID: https://orcid.org/0009-0009-7667-4726

Samantha Shulhan: Medical Student, University of Texas at Austin Dell Medical School

Bridget Fitzgerald, MS: Medical Student, University of Texas at Austin Dell Medical School

Uma Jacobs: Medical Student, University of Texas at Austin Dell Medical School

Alanna Boulton, MSHS: Director of Operations for Mental Health and Substance Use, Central Health

Drew Coyne, MD: Clinical Assistant Professor, Department of Surgery and Perioperative Care, University of Texas at Austin Dell Medical School

Lloyd Berg, PhD: Chief, Division of Psychology, Department of Psychiatry and Behavioral Sciences, University of Texas at Austin Dell Medical School

Copyright © 2026 Burr et al. This is an open-access publication distributed under the terms of the Creative Commons Attribution license.

Kirk Von Sternberg, PhD: Associate Director, Health Behavior Research and Training Institute, Steve Hicks School of Social Work, University of Texas at Austin

John Weems, MD: Associate Director of Addiction Medicine, CommunityCare Health Centers

Jacki Hecht, RN, MSN: Project Coordinator, Johnson-Turpin Center for Gerontological Nursing, University of Texas School of Nursing

Patrick Kennedy, MSSW: Graduate Research Assistant, Steve Hicks School of Social Work, University of Texas at Austin

Mary M. Velasquez, PhD: Director, Health Behavior Research and Training Institute, Steve Hicks School of Social Work, University of Texas at Austin

Acknowledgments
We would like to acknowledge the following people for their contributions: Elizabeth M. O'Gara, Former Director, Standardized Patient Program for Education and Assessment for the Health Professions, University of Texas at Austin Dell Medical School; Nickole Hines-Staples, Director, Standardized Patient Program for Education & Assessment for the Health Professions, University of Texas at Austin Dell Medical School; Nicole Manley, Director, Simulation and Skills Center (SSC), School of Nursing, University of Texas at Austin; Susan Salas, Project Manager, Health Behavior Research and Training Institute, Steve Hicks School of Social Work, University of Texas at Austin; and Diana Ling, Health Communication Professional, Health Behavior Research and Training Institute, Steve Hicks School of Social Work, University of Texas at Austin.

Disclosures
None to report.

Funding/Support
None to report.

Prior Presentations
Jacobs U, Shulhan S, Burr S, et al. Developing a screening, brief intervention, and referral to treatment curriculum for medical students. Presented at: Interprofessional Health Showcase; April 2025; Austin, TX.

Shulhan S, Jacobs U, Burr S, et al. Piloting a screening, brief intervention, and referral to treatment curriculum for medical students. Presented at: Texas College of Emergency Physicians ConnecTX Annual Meeting; April 2025; Austin, TX.

Burr S, Shulhan S, Jacobs U, et al. Piloting a screening, brief intervention, and referral to treatment curriculum for medical students during emergency department rotation. Presented at: Association for Multidisciplinary Education and Research in Substance Use and Addiction Conference; October 2024; Chicago, IL.

Burr S, Shulhan S, Jacobs U, et al. Piloting a screening, brief intervention, and referral to treatment curriculum for medical students during emergency department rotation. Presented at: Interprofessional Education Showcase; April 2024; Austin, TX.

Ethical Approval
The University of Texas at Austin Institutional Review Board reviewed this project.

References

1. Abuse Substance, Administration Mental Health Services. *Screening, Brief Intervention and Referral to Treatment (SBIRT) in Behavioral Healthcare*. SAMHSA; 2011. *Accessed* November 14, 2025. https://www.samhsa.gov/sites/default/files/sbirtwhitepaper_0.pdf
2. Levy SJL, Williams JF; Committee on Substance Use and Prevention, et al. Substance use screening, brief intervention, and referral to treatment. *Pediatrics*. 2016;138(1):e20161211. https://doi.org/10.1542/peds.2016-1211
3. US Preventive Services Task Force; Curry SJ, Krist AH, et al. Screening and behavioral counseling interventions to reduce unhealthy alcohol use in adolescents and adults: US Preventive Services Task Force recommendation statement. *JAMA*. 2018; 320(18):1899-1909. https://doi.org/10.1001/jama.2018.16789
4. US Preventive Services Task Force; Krist AH, Davidson KW, et al. Screening for unhealthy drug use: US Preventive Services Task Force recommendation statement. *JAMA*. 2020;323(22): 2301-2309. https://doi.org/10.1001/jama.2020.8020
5. Patnode CD, Perdue LA, Rushkin M, O'Connor EA. *Screening for Unhealthy Drug Use in Primary Care in Adolescents and Adults, Including Pregnant Persons: Updated Systematic Review for the U.S. Preventive Services Task Force*. Agency for Healthcare Research and Quality (US); 2020. Accessed November 14, 2025. http://www.ncbi.nlm.nih.gov/books/NBK558174/
6. Centers for Medicare and Medicaid Services. *Screening, Brief Intervention & Referral to Treatment (SBIRT) Services*. CMS; 2025. Accessed November 14, 2025. https://www.cms.gov/outreach-and-education/medicare-learning-network-mln/mlnproducts/downloads/sbirt_factsheet_icn904084.pdf
7. Screening, Brief Intervention, and Referral to Treatment (SBIRT) grantees. SAMHSA. Accessed November 14, 2025. https://www.samhsa.gov/substance-use/treatment/sbirt/grantees
8. Balbi AM, Gak AE, Kim ES, et al. Brief motivational interviewing for substance use by medical students is effective in the emergency department. *J Emerg Med*. 2019;57(1):114-117. https://doi.org/10.1016/j.jemermed.2019.03.032
9. Clauser J, Richardson BB, Odom-Maryon T, et al. Standardized patient simulation using SBIRT (Screening, Brief Intervention, and Referral for Treatment) as a tool for interprofessional learning. *MedEdPORTAL*. 2020;16:10955. https://doi.org/10.15766/mep_2374-8265.10955
10. Wamsley M, Treit K, Satterfield J, et al. Three standardized patient cases to measure Screening, Brief Intervention and Referral to Treatment (SBIRT) skills in primary care residents. *MedEdPORTAL*. 2011;7:9005. https://doi.org/10.15766/mep_2374-8265.9005

Copyright © 2026 Burr et al. This is an open-access publication distributed under the terms of the Creative Commons Attribution license.

11. Soskin P, Duong D. Social worker as SBIRT instructor to emergency medicine residents. *MedEdPORTAL*. 2014;10:9840. https://doi.org/10.15766/mep_2374-8265.9840

12. Acquavita SP, Richardson GB, Smith R, et al. Outcomes of an interprofessional SBIRT training program: knowledge attainment and perceived competence for practice. *Subst Abuse*. 2021;42(4):935-943. https://doi.org/10.1080/08897077.2021.1900982

13. Edwards EJ, Arora B, Green P, Bannatyne AJ, Nielson T. Teaching brief motivational interviewing to medical students using a pedagogical framework. *Patient Educ Couns*. 2022;105(7): 2315-2319. https://doi.org/10.1016/j.pec.2022.01.012

14. Gainey S, Muzzy W, Dooley M, et al. Outcomes and lessons learned from an interprofessional student training program in Screening, Brief Intervention, and Referral to Treatment (SBIRT) at an academic health sciences center. *Nurse Educ Today*. 2022;111:105323. https://doi.org/10.1016/j.nedt.2022.105323

15. Spangler JG, George G, Foley KL, Crandall SJ. Tobacco intervention training: current efforts and gaps in US medical schools. *JAMA*. 2002,288(9):1102-1109. https://doi.org/10.1001/jama.288.9.1102

16. Hivert MF, Arena R, Forman DE, et al. Medical training to achieve competency in lifestyle counseling: an essential foundation for prevention and treatment of cardiovascular diseases and other chronic medical conditions: A scientific statement from the American Heart Association. *Circulation*. 2016; 134(15):e308-e327. https://doi.org/10.1161/CIR.0000000000000442

17. Zayyan M. Objective structured clinical examination: the assessment of choice. *Oman Med J*. 2011;26(4):219-222. https://doi.org/10.5001/omj.2011.55

18. Understanding motivational interviewing. Motivational Interviewing. Accessed November 14, 2025. https://motivationalinterviewing.org/understanding-motivational-interviewing

19. Kirkpatrick JD, Kirkpatrick WK. *Kirkpatrick's Four Levels of Training Evaluation*. Association for Talent Development Press; 2016.

20. Moyers TB, Rowell LN, Manuel JK, Ernst D, Houck JM. The Motivational Interviewing Treatment Integrity Code (MITI 4): rationale, preliminary reliability and validity. *J Subst Abuse Treat.* 2016;65:36-42. https://doi.org/10.1016/j.jsat.2016.01.001

Received: August 15, 2025
Accepted: November 21, 2025
Published: January 13, 2026

Copyright © 2026 Burr et al. This is an open-access publication distributed under the terms of the Creative Commons Attribution license.
